# N-terminal protein-macrocycles enabled by conjugate addition/ring expansion cascade reactions

**DOI:** 10.1039/d5sc08044d

**Published:** 2025-12-16

**Authors:** Owen R. Hughes, Afzaal Tufail, Esme Hutton, Joe Nabarro, Rachel Howarth, Nicholas D. Yates, Adrian C. Whitwood, Craig N. Robson, Nathalie Signoret, Martin A. Fascione, Christopher D. Spicer, William P. Unsworth

**Affiliations:** a Department of Chemistry, University of York York YO10 5DD UK martin.fascione@york.ac.uk chris.spicer@york.ac.uk william.unsworth@york.ac.uk; b York Biomedical Research Institute, University of York York YO10 5DD UK; c Northern Institute for Cancer Research, Newcastle University Newcastle Upon Tyne NE2 4HH UK; d Hull York Medical School, University of York York YO10 5DD UK

## Abstract

The development of methods for chemical modification of proteins has enabled transformative advancements in the fields of chemical biology, cell biology and biomedicine. Herein we demonstrate that simple, easy-to-prepare acryloyl imides can be used as reagents for N-terminal protein bioconjugation that exploit conjugate addition/ring expansion (CARE) cascade reactions to afford novel protein-macrocycle conjugates. The CARE approach advantageously proceeds with high N-terminal selectivity, affords the formation of stable bioconjugates driven by irreversible ring expansion, and facilitates access to modified proteins appended with complex, functionalized medium sized rings. The utility of this late stage protein macrocycle diversification is showcased in nanobody-based imaging of cancer tissue and modulation of a chemokine–receptor interaction, uniquely decoupling GPCR endocytosis from phosphorylation. As such the CARE bioconjugation represents a powerful and versatile platform with broad potential application in the construction of tools for dissecting biological mechanisms, and biologics with new therapeutic modalities.

## Abstract 2

The development of methods for chemical modification of proteins has enabled transformative advancements in the fields of chemical biology, cell biology and biomedicine.

## Graphical abstract

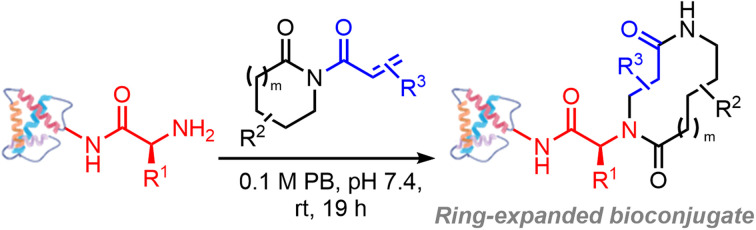

## Introduction

Protein bioconjugation has revolutionized modern day biomedical and biotechnological research. Through the union of small molecules and large macromolecules, chemical protein modification can deliver bioconjugates with altered, or novel, structure and function, greatly expanding applications beyond what can be achieved through the 20 canonical amino acids.^1^ Macrocycles are often considered the bridge between small molecules and proteins, combining the functionality and synthetic accessibility of the former with the 3D-shape diversity of the latter, leading to wide ranging applications in mechanistic biology and drug discovery.^2^ However methods for construction of covalent macrocycle-protein bioconjugates are rare,^2*c*,*d*^ in part due to the steric challenges presented in bringing the two partners together. Novel strategies providing access to this on-protein chemical space would therefore be of value.

In this context, protein N-termini represent attractive targets for chemical modification. These uniquely reactive sites are chemically and sterically accessible in up to 20% of the eukaryotic proteome, including many secreted proteins.^3^ Reactions targeting N-termini can be broadly split into two categories: (i) specific reactions, relying on the participation of the proximal α-amide of the protein backbone; and (ii) selective reactions that exploit the lower p*K*_a_ of the N-terminal ammonium (6.0–8.0) relative to the ε-ammonium of lysine side-chains (∼10.5). This difference in p*K*_a_ leads to α-amines exhibiting increased nucleophilicity at near-neutral pH,^3^ allowing reaction with electrophilic small molecules probes like Michael acceptors. However, while much progress has been made in this area in recent years,^4^ leading methods for N-terminal protein modification based on aza-Michael reactions for example, often suffer from low selectivity and/or conjugate instability, due in part to the reversibility in the key aza-Michael step.^5^

**Scheme 1:**
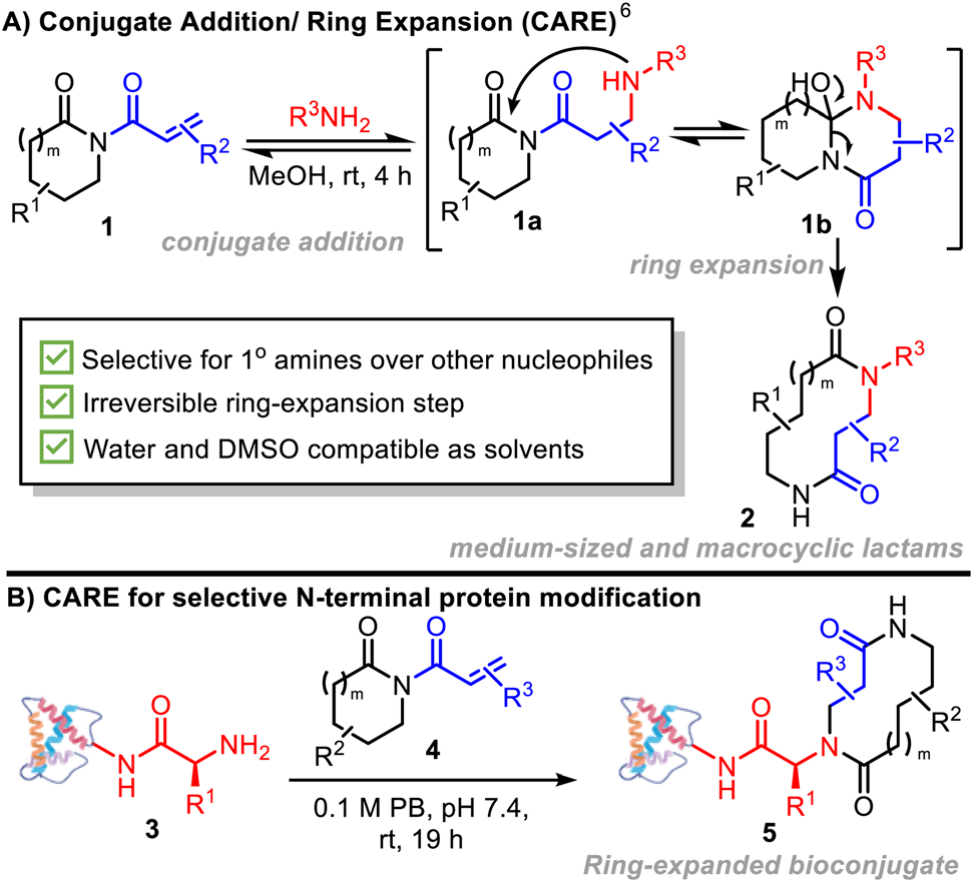
(A) CARE cascades of acryloyl imides with primary amines (previous work); (B) this work: N-terminal protein-macrocycles enabled by conjugate addition/ring expansion cascade reactions.

Herein, we introduce a new method for N-terminal macrocyclic bioconjugation using the Conjugate Addition/Ring Expansion (CARE) cascade strategy (Scheme 1A).^6–8^ Our CARE approach starts with a reversible conjugate addition reaction of a primary amine with an acryloyl imide (1 → 1a) – at this stage, akin to established amine bioconjugation methods that make use of Michael acceptors. However, a key design feature of our acryloyl imide probes is that following conjugate addition, the resulting intermediate 1a is primed to undergo cyclisation (1a → 1b) and irreversible ring expansion (1b → 2) *via* a cascade reaction, to form a macrocycle or medium-sized ring.^6^ This feature addresses one of the major limitations of established N-terminal targeting approaches, where reversibility can lead to lower conversions, necessitating more forcing conditions, and an increase in off-target labelling, particularly at lysine.^9^

The successful realization of the CARE strategy for N-terminal protein bioconjugation using easy-to-prepare acryloyl imides 4 is described (Scheme 1B). Selectivity for N-terminal modification over lysine side chains, the formation of stable bioconjugates driven by an irreversible ring expansion step, and the ability to generate proteins modified by macrocycles or medium-sized rings of increasing complexity are key advantages. The ability of the CARE approach to generate functional and medicinally-relevant constructs is demonstrated in two forms – firstly in the labelling of a prostate specific membrane antigen-targeting nanobody for selective imaging of cancerous tissue; and secondly, in the preparation of modified analogues of the key human chemokine CCL5 which modulate cell surface receptor activation and processing in a macrocycle-dependent manner.

## Results and discussion

**Fig. 1:**
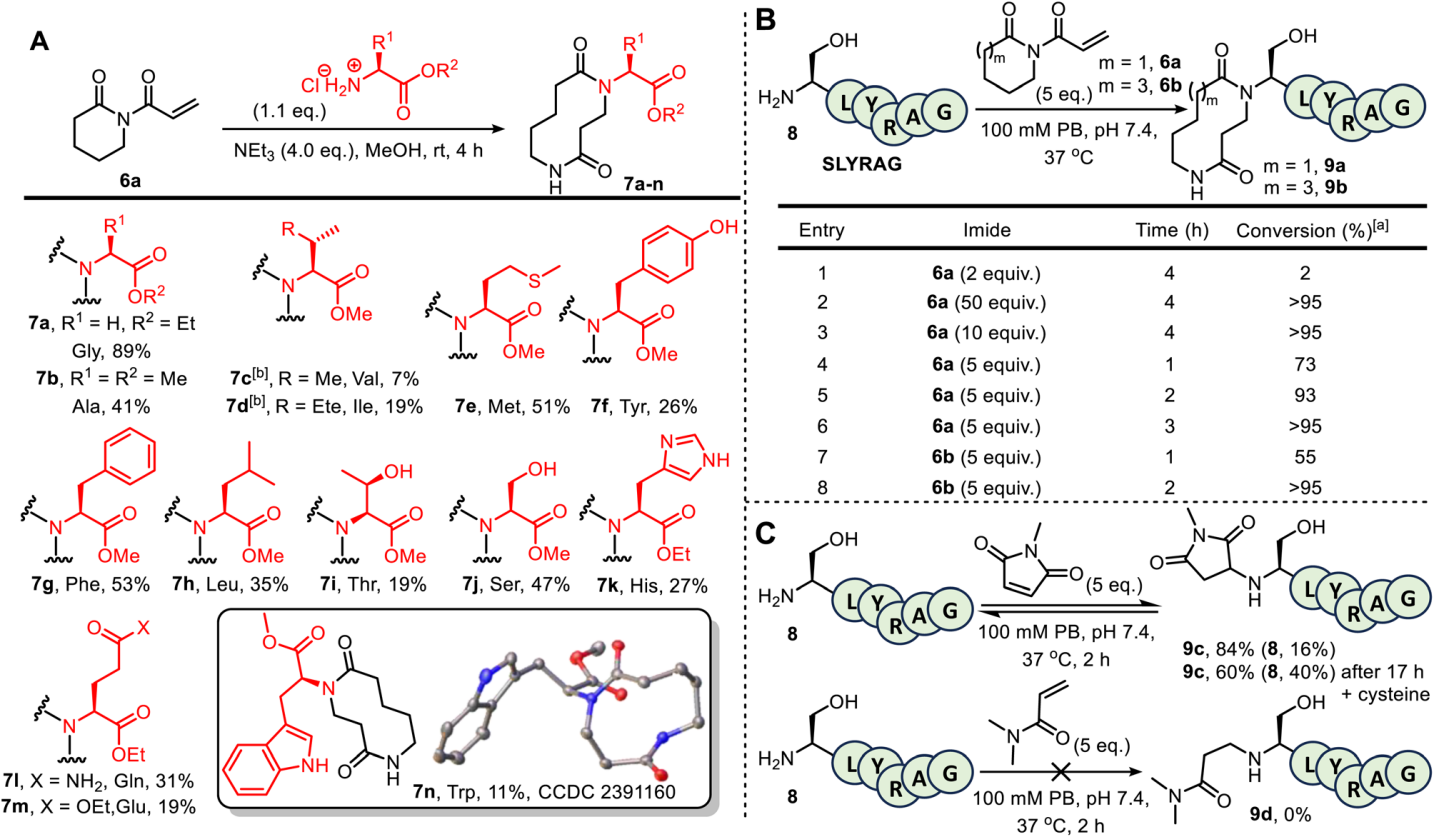
(A) Amino ester hydrochloride salts used as substrates in CARE with 6a: [a] Amino acid hydrochloride salt (1.1 eq.), MeOH (≈0.5 M), triethylamine (4.0 eq.) and imide 6a (1.0 eq.) were stirred for 4 h at room temperature, before concentration *in vacuo* and purification by FCC; [b] reaction performed in DCM (≈0.5 M) instead of MeOH; (B) CARE of imides 6a and 6b with SLYRAG 8. (C) Other Michael acceptors for the modification of SLYRAG 8 (for MS data see Fig. S14–16).

Before embarking on bioconjugation studies, we first tested whether our published small molecule CARE method is compatible with amines derived from selected proteinogenic amino acids. Thus, 6-membered ring acryloyl imide 6a was reacted with a variety of amino ester hydrochloride salts, under the conditions summarised in Fig. 1A. This approach afforded the expected 10-membered ring bis-lactams 7a–n, all obtained using the standard CARE protocol in methanol. In the case of tryptophan-derived product 7n, X-ray crystallographic data was obtained to confirm that the expected ring-expanded product formed.^10^ Although the isolated yields for these reactions were mixed (7–89%), no competing reactions on the amino acid side chains were observed in all but one of the reactions tested. A notable exception was when the CARE reaction was attempted using cysteine methyl ester hydrochloride, in which side products arising from competing thio-Michael reactions were observed, as expected (see SI Section 2, page S22). Although this is limiting when proteins contain free surface cysteines, the presence of such residues is rare, as most cysteine thiol groups are exist as stable disulfide bridges.^11^ Cognizant that the conditions for protein bioconjugation would differ from those used on small molecules, these test reactions were unoptimized, with reduced nucleophilicity for amino acids bearing large or branched side chains due to steric crowding (most pronounced for the formation of 7c) thought to be the major reason for lower conversions in some examples.^6*a*^ Lysine and arginine derivatives are notable absentees in this amino ester screen; however, lysine- and arginine-containing 6-mer peptides feature later in the manuscript (see Fig. 1B and SI Section 5), confirming their compatibility. The failure of imide 6a to react with dimethylguanidine sulphate suggests indicates likely compatibility with arginine (see SI Section 2, page S23).

Attention next turned to assessing the viability of the CARE method on a short peptide, using the 6-mer SLYRAG 8.^12^ Initially, SLYRAG 8 was reacted at 37 °C in pH 7.4 phosphate buffer with the same acryloyl imide 6a used in the small molecule studies. Conversion to 9a was determined by LC-MS (Fig. 1B). Only 2% conversion of SLYRAG 8 into its N-terminally modified derivative 9a was observed when 2 equivalents of imide 6a were used (entry 1), but increasing to 10 or 50 equivalents of 6a (entries 2 and 3) enabled full conversion into 9a after 4 h. Good or full conversion could also be achieved using fewer equivalents of 6a (entries 4–6); *e.g*. when SLYRAG 8 was reacted with 5 equivalents of 6a, 73% conversion into 9a was observed within 1 h, and full conversion after 3 h. Similar experiments were also performed using 5 equivalents of the 8-membered ring acryloyl imide 6b, and this reagent was fully converted into 12-membered ring modified SLYRAG derivative 9b within 2 h (entries 7 and 8). To test conjugate stability, 9b was incubated at 37 °C for 19 h in the presence of 10 equiv. of cysteine. If the CARE conjugation was reversible, degradation of the product over time would be expected, with thiol trapping of the Michael acceptor. However, such degradation was not observed, demonstrating that the conjugate is stable and that CARE is irreversible under these conditions (see SI and Fig. S17).

To compare 6a and 6b with an established Michael acceptor used for protein bioconjugation, the reaction of SLYRAG 8 with 5 equivalents of *N*-methylmaleimide initially led to an 84% conversion to modified peptide 9c over 2 h with 16% starting material 8 remaining (Fig. 1C). Furthermore, after incubation with cysteine for 19 h, a 24% reduction in product 9c and increase in starting material 8 to 40% was observed (see SI and Fig. S17), indicative of reversibility of the bioconjugate formation, and/or instability of the conjugate. However, unequivocal characterisation of ring expansion conjugate stability and the kinetics of any breakdown will require more detailed study, notably exploring the potential impact of protein sequence and structure, pH changes, and the stability in complex biological milieu such as serum, which has previously been achieved using FRET-based reporter assays.^13^ Of additional note, the reaction of SLYRAG with *N*,*N*-dimethylacrylamide failed to generate any labelled peptide 9d, demonstrating the beneficial increased reactivity of the acryloyl imides 6a and 6b relative to acrylamide electrophiles.

**Fig. 2:**
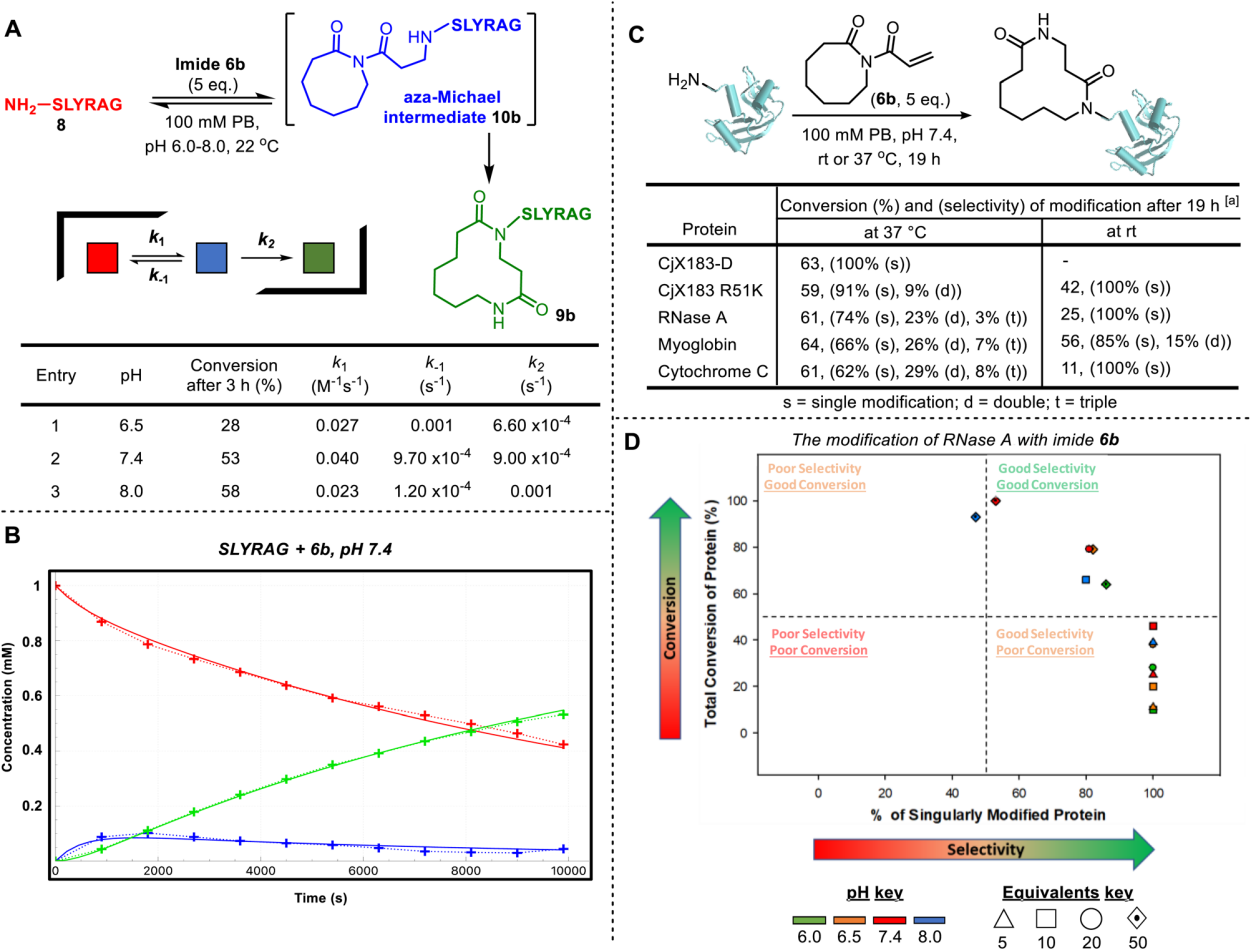
CARE cascades of acryloyl imides with peptides and proteins. (A) CARE of imide 6b with SLYRAG 8. Reactions were run at 1 mM under second-order conditions at pH 6.5, 7.4, 8.0. Reaction progress was monitored by LC-MS (see SI Section SI5). Rate constants were obtained from a deterministic algorithm whose numerical solver computes changes to molecular species concentration and assigns values for each reaction based on a ‘trial-and-error’ approach. Full details, including errors of fitting, are provided in the SI and Fig. SI9; (B) a plot of molecular species concentration against time for entry 2 (for similar plots for entries 1 and 3, see SI and Fig. SI9); (C) protein bioconjugation with CARE using imide 6b: ^[a]^ 6.7 µL of protein (150 µM stock in 0.1 M PB, pH 7.4), 4 µL of PB (0.5 M stock, pH 7.4) and 7.3 µL of HPLC-grade water is added to 2 µL of imide 6b (50 µM stock in DMSO) and incubated at the desired temperature, 1000 rpm for ∼19 h before an aliquot is taken and analysed by LC-MS (for full experimental and LC-MS details, see SI, Section SI8); (D) screening conditions for RNase A bioconjugation with imide 6b. Reactions were run at 50 µM of RNase A at pH 6.0, 6.5, 7.4 and 8.0 with 5, 10, 20 or 50 equivalents of imide 6b (stock adjusted accordingly in DMSO) and incubated at room temperature, 1000 rpm for ∼19 h before an aliquot was taken and analysed by LC-MS (for full experimental and LC-MS details, see SI, Section SI8; for tabulated data that make up the plot, see SI, Section S19).

By investigating LC-MS data for the reaction of 6b with SLYRAG 8 in more detail, we identified an additional peak with the same mass as the product, which appeared at early time points and decreased in intensity over time. We attributed this peak to the intermediate aza-Michael product 10b, which subsequently undergoes ring-expansion to generate the amide in 9b (Fig. 2A). Using the concentrations of the starting peptide 8, intermediate 10b, and final product 9b over time, we fitted conversions to a two-step kinetic model and extracted rate constants for the aza-Michael (*k*_1_), retro-Michael (*k*_−1_), and ring-expansion steps (*k*_2_) at pHs 6.5, 7.4 and 8.0 (Fig. 2A table and Fig. 2B for a plot at pH 7.4 – for further details see SI Section SI5). This analysis highlights the importance of ring expansion in driving efficient peptide modification – in the absence of this irreversible step, a reaction driven purely by the equilibrium between Michael and retro-Michael reactions would only be expected to reach a conversion of ∼20% (equilibrium constant *K*_1_ = 41 M^−1^). The bioconjugation of SLYRAG 8 with 6b was found to be slower at pH 6.5, due to both reduced aza-Michael addition and accelerated retro-aza-Michael reaction rates, as would be expected with increased amine protonation at reduced pH. Though conversions at pH 7.4 and 8.0 were comparable, we chose to perform all further experiments at pH 7.4 to maximise N-terminal selectivity.

In addition to SLYRAG 8, CARE reactions of 6a with other 6-mer peptides with different N-terminal amino acids were also tested (see SI and Fig. S20). Successful conversion into the ring-expanded product was seen in all cases, with overall conversion governed by a combination of factors: while a decreased α-amine p*K*_a_ led to an increased rate of conjugate addition (*e.g. k*_1_ = 0.1 M^−1^ s^−1^ for MLYRAG, *vs.* 0.04 M^−1^ s^−1^ for SLYRAG 8), this did not correlate with an overall increase in conversion due to a slowed rate of ring expansion, which appeared to be influenced by the size of the N-terminal side-chain (*e.g. k*_2_ = 1 × 10^−4^ s^−1^ for WLYRAG, *vs.* 9 × 10^−4^ s^−1^ for SLYRAG 8), in line with our observations on small molecules.^6*a*^ We also investigated the reaction of the *ε*-amine of an internal lysine residue using a peptide bearing an N-terminal acetate cap (see SI, Section S20). In this case, negligible conversion was observed over 3 hours. Both a reduced rate of Michael addition and accelerated retro-Michael reaction were observed, leading to a greater than 2-order of magnitude decrease in *K*_1_ relative to the modification of an N-terminal α-amine (0.12 M^−1^*vs.* 41 M^−1^ for SLYRAG 8). This is presumably due to increased amine protonation as a result of the higher p*K*_a_ of ε-amines, in line with the observations of reduced N-terminal labelling at slightly acidic pH described above. Cumulatively, these results on model peptides indicated a strong preference for N-terminal over lysine modification, with selectivity expected to be reduced with increasing pH.

Next, we translated the CARE strategy to proteins, where achieving selectivity was expected to be more challenging. A panel of model proteins, RNase A, myoglobin, CjX183-D, and cytochrome C, all with accessible *N*-termini,^5^ were each incubated with acryloyl imide 6b (5 equiv.) at pH 7.4. Reactions were analysed after 19 h by intact protein LC-MS, and in all cases successful protein modification was observed (Fig. 2C). For RNase A, myoglobin, and cytochrome C, reactions at 37 °C led to a modest degree of double and triple modification, presumably due to off-target modification at lysine residues. However, this could be avoided/minimized by performing the reactions at room temperature, leading to much better selectivity for single modification. CjX183-D is an interesting model substrate, since the wild-type protein contains no naturally occurring lysine residues, and as a result single-site modification was observed even at 37 °C. Introduction of a single lysine residue in the mutant protein CjX183 R51K led to the appearance of a small amount (9%) of double modification at 37 °C, reinforcing the hypothesis that ε-amine modification is the predominant competing reaction.

We subsequently investigated the modification of RNase A in more detail (Fig. 2D). As would be expected based on our observations on peptides and the role of α- and ε-ammonium p*K*_a_s, increasing the equivalents of 6b and increasing pH both led to higher conversion, but with a selectivity trade-off. Importantly though, conversions of ∼50% to the singly modified protein could be achieved at both pH 6 and pH 7.4, through careful control of reaction conditions.

**Fig. 3:**
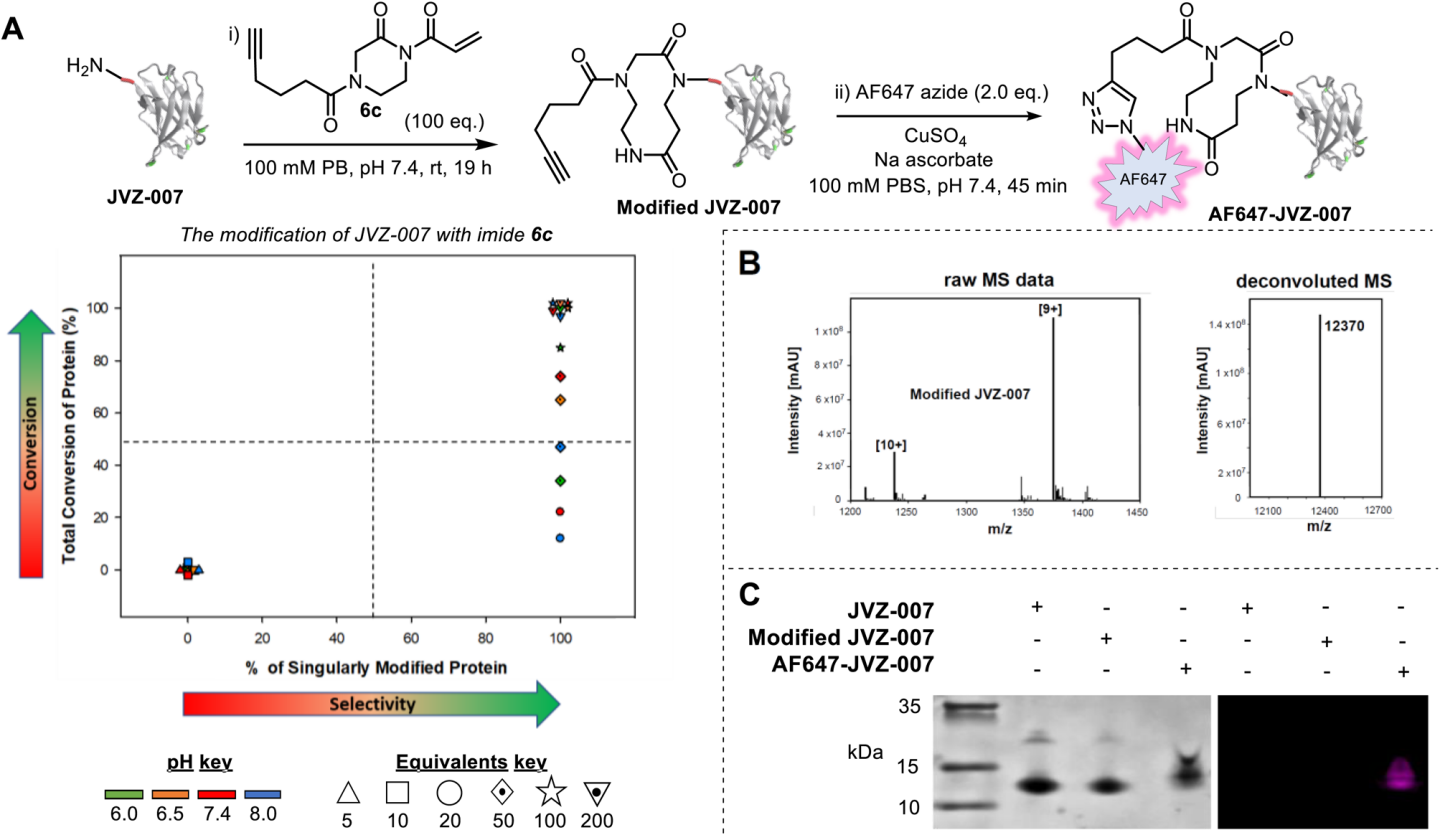
Bioconjugation of JVZ-007 with imide 6c. (A) Modification of JVZ-007 with imide 6c and fluorescent tagging. (i) JVZ-007 (169 µM stock in PBS; 50 µM final) and imide 6c (100 eq.) in phosphate buffer (0.5 M stock; 0.1 M final) were shaken in an incubator at room temperature overnight (∼19 h). (ii) Modified JVZ-007 (40 µM stock in 100 mM PBS, pH 7.4; 20 µM final), sodium ascorbate (1.25 mg mL^−1^), CuSO_4_ (4 mM) and AF-647 (5 mM stock in DMSO; 40 µM final (2.0 eq.)) in a 500 µL Eppendorf tube protected from light (with foil) were rotated on a fan rotator for 45–60 min. The crude reaction mixtures were purified by dialysis. For the conditions screen, reactions were run at 50 µM of protein at pH 6.0, 6.5, 7.4 and 8.0 with 5, 10, 20, 50, 100 or 200 equivalents of imide 6c: 6.7 µL of JVZ-007 (∼150 µM stock in 0.1 M PB, pH 7.4), 4 µL of PB (0.5 M stock, pH adjusted accordingly) and 7.3 µL of HPLC-grade water was added to 2 µL of imide 6c (stock adjusted accordingly in DMSO) and incubated at room temperature, 1000 rpm for ∼19 h before an aliquot was taken and analysed by LC-MS. For tabulated data that make up the plot, see SI, Section S19; (B) left: raw ESI-MS for modified JVZ-007 showing [10+] and [9+] charge adducts; Right: charge deconvoluted ESI-MS showing >95% single modification. Expected mass for singularly modified JVZ-007 = 12 370 Da; (C) analysis of JVZ-007, Modified JVZ-007, and AF647-JVZ-007 by a TSDS-PAGE gel and visualisation by initial fluorescent imaging (right) followed by Coomassie staining (left).

To expand upon these results and demonstrate the utility of our approach, we next synthesized acryloyl imide 6c bearing a pendant alkyne (Fig. 3A, see SI, Section SI2 and SI3 for preparative details). The alkyne group provides a useful intermediate for reagent diversification, as well as a handle for post-bioconjugation functionalisation *via* copper-catalysed azide-alkyne cycloaddition (CuAAC). The conjugation of acryloyl imide 6c was first tested on RNase A, a protein already shown to be compatible with CARE, and interestingly, selectivity for N-terminal modification was greater than it was when using the simpler imide 6b (see SI and Fig. S24). Bioconjugation of 6c was then tested using a new protein, nanobody JVZ-007, and excellent conversions were achieved with imide 6c under several of the conditions tested (Fig. 3A–C). Protein digestion of modified JVZ-007 demonstrated that CARE had taken place at the *N*-terminus of the protein (see SI, Fig S23). Furthermore, the conjugate was found to be stable to overnight dialysis at 37 °C with no evidence of degradation back to the starting protein, indicative of successful, irreversible ring-expansion (see SI, Section SI9). The accessibility of the pendant alkyne on modified JVZ-007 for CuAAC and on-protein derivatisation was also demonstrated, *via* the attachment of an AlexaFluor 647 azide, resulting in fluorescent labelling of JVZ-007 (to form AF647-JVZ-007, Fig. 3A and C).

**Fig. 4:**
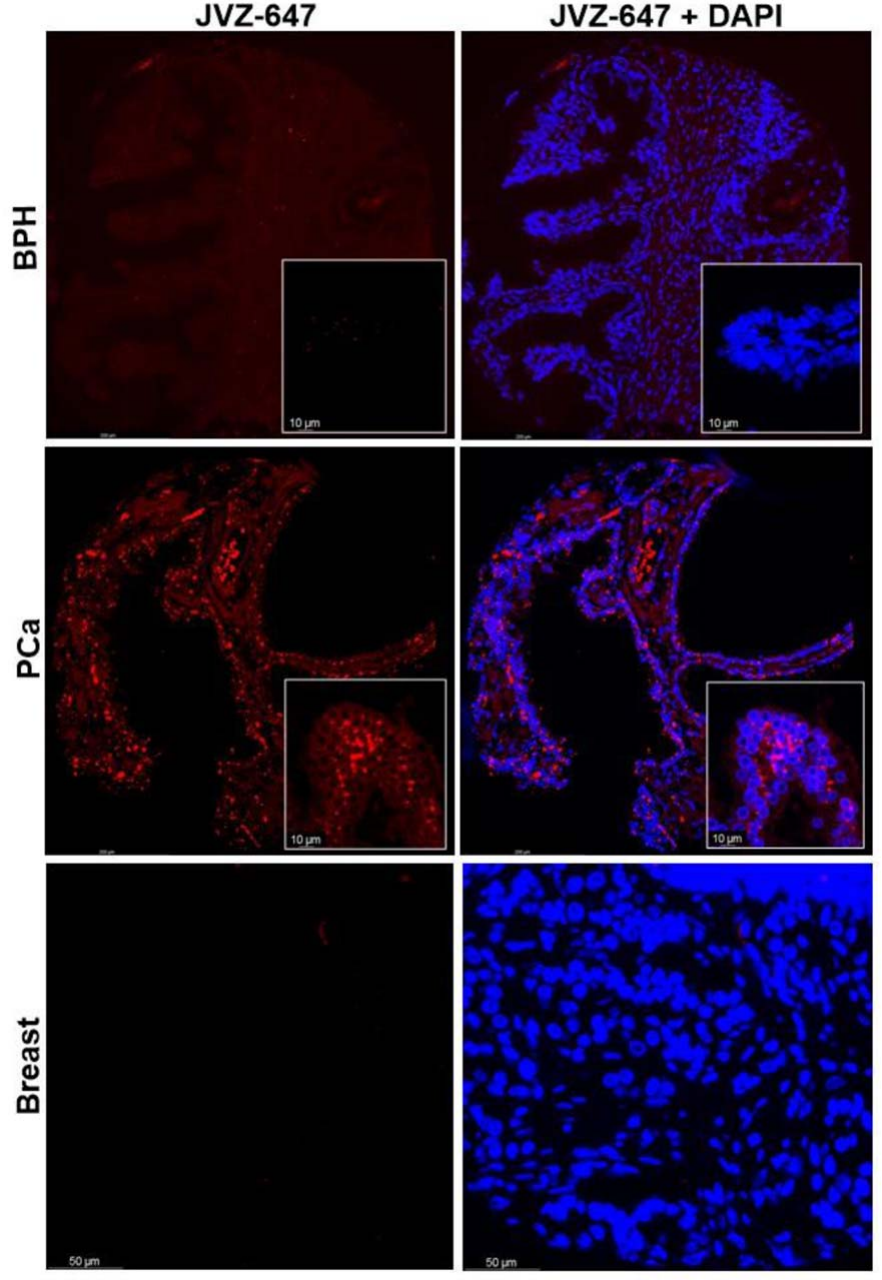
Immunofluorescent staining of BPH, PC_a_ and breast cancer tissue using the fluorescent nanobody AF647-JVZ-007 (left, red) and DAPI nuclear counterstain (right, blue).

JVZ-007 is an important target for bioconjugation as it selectively binds prostate-specific membrane antigen (PSMA) which is overexpressed on the surface of prostate cancer cells.^14,15^ With the fluorescent labelled protein conjugate in hand AF647-JVZ-007, this was used to successfully visualise PSMA on LNCaP prostate cancer cells, which endogenously express high levels of the antigen, initially *via* fluorescence microscopy, (See SI and Fig. S25).^15^ Subsequent immunofluorescent staining of tissue from prostate cancer (PC_a_) patients was compared with tissue from patients with non-cancerous benign prostate hyperplasia (BPH) (Fig. 4), and demonstrated the utility of the fluorescently modified nanobody for identifying PC_a_ tissue. PC_a_ cells have been established to present increased levels of PSMA on their surface relative to BPH tissue,^16^ with a lack of staining in a control breast cancer sample confirming specificity for PSMA. These results confirmed that the mild bioconjugation conditions used in the tandem CARE-CuAAC bioconjugation did not preclude the nanobody from still recognising its target receptor.

**Fig. 5:**
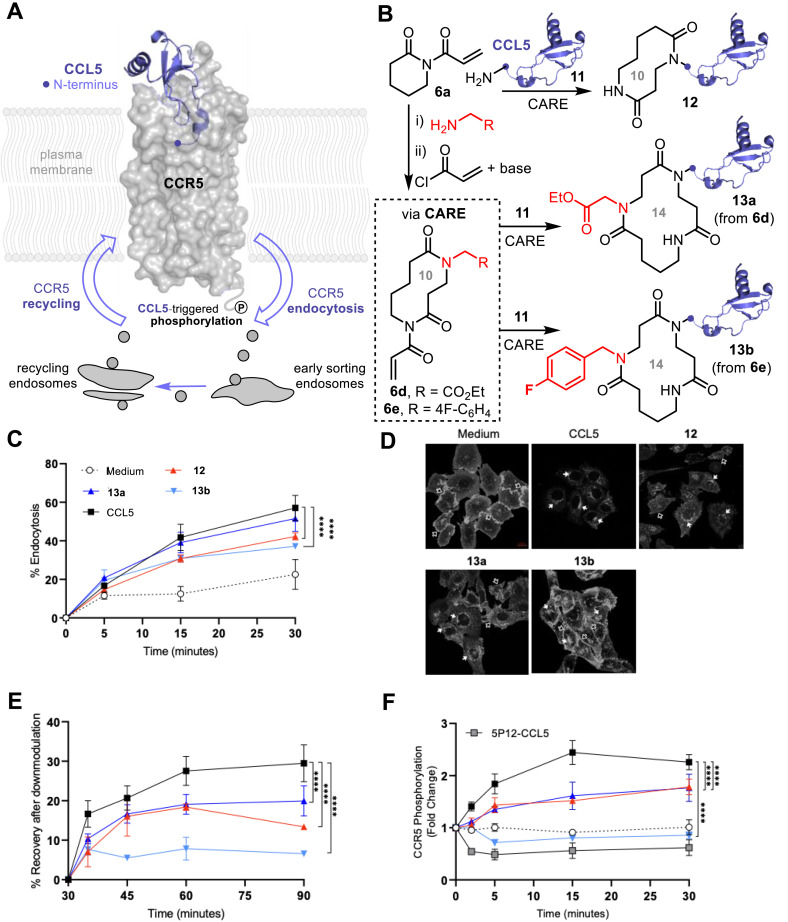
CARE bioconjugation in the exploration of CCL5–CCR5 binding and receptor recycling. (A) Wild type CCL5 N-terminal insertion and agonist binding into the extracellular CCR5 cavity induces receptor phosphorylation, followed by endocytosis and recycling of the activated receptor *via* early-sorting and recycling endosomes; (B) N-terminal CARE reactions of CCL5 11 to afford 10-membered ring-CCL5 conjugate 12, and 14-membered macrocycle conjugates 13a and 13b; (C) kinetics of CCR5 endocytosis upon treatment as indicated (mean of *n* = 3 experiments); (D) confocal images visualising changes in CCR5 distribution from the cell-surface to perinuclear recycling endosomes (open and filled arrows, respectively) after 30 min of treatment (scale bar = 10 µm); (E) kinetics of CCR5 recycling and cell-surface recovery following 30 min of treatment (mean of *n* = 3 experiments); (F) kinetics of CCR5 phosphorylation on Ser349 upon treatment, using 5P12-RANTES as a CCR5-derivative control not inducing phosphorylation (mean of *n* = 2 experiments). **** *P* ≤ 0.0001 two-way ANOVA with Dunnett secondary test.

Finally, we explored the utility of the CARE approach for accessing proteins modified with functionalized macrocycles of increasing size and structural diversity. To do this, we focused on the modification of the human CCL5 chemokine (also known as RANTES), which binds to the G-protein coupled chemokine receptor CCR5 (Fig. 5A). Upon CCL5-CCR5 binding, intracellular signaling is triggered leading to directed cell migration or chemotaxis.^17^ CCL5-bound CCR5 is rapidly desensitised through downmodulation, a process including CCR5 phosphorylation in the cytoplasmic tail (residue Ser349), endocytosis, and recycling with cell surface re-accumulation of de-activated receptors.^18^ CCR5 also serves as the principal co-receptor for the human immunodeficiency virus (HIV), and CCR5-binding “antagonists” are therefore highly desirable as *anti*–HIV therapies^19–22^ This includes modified versions of CCL5, like 5P12-RANTES, that have previously been shown to be potent inhibitors of HIV infection.^19^ As the N-terminal region of CCL5 forms a high affinity interaction by deep insertion into the extracellular CCR5 ligand pocket,^23^ we hypothesized that CARE modification of CCL5 with derivatised macrocycles of varying size and structure might therefore augment or alter the receptor activation and trafficking process. We initially performed N-terminal CARE ring expansions on CCL5 11 with underivatized imide 6a to afford CCL5 bearing a 10-membered macrocycle, 12, with full conversion (Fig. 5B). We also performed ‘off-protein’ CARE reactions on 6a using the method summarised earlier in Fig. 1C; the resulting 10-membered ring bis-lactams were subsequently acylated with acryloyl chloride under basic conditions (see SI, Section SI3 for synthetic details). These functionalised, ring-expanded reagents 6d and 6e were then used for CARE modification of CCL5, thus undergoing a further ring expansion. In both cases, N-terminal CARE provided access to 14-membered macrocycle-modified CCL5, bearing either an ethyl ester (13a) or an aryl fluoride (13b) side chain.

Intriguingly, when we compared CCR5 stimulation by the macrocycle-CCL5s 12–13 to wild-type CCL5 11, using phosphorylation, endocytosis and recycling assays (Fig. 5C–F), we observed similar agonistic abilities indicating that introduction of the macrocycles did not disrupt the binding interaction in the ligand pocket. However, our experiments exposed marked differences between wild-type and labelled CCL5s, with reduced receptor endocytosis, recycling and phosphorylation observed for 12–13 compared to 11 (Fig. 5C, E and F). Most notable, however, was the 14-membered aryl fluoride modified macrocycle-conjugate 13b, which did not elicit any CCR5 phosphorylation of Ser349 residue (Fig. 5F), which is the target of G-protein coupled receptor kinases (GRKs) prior to agonist-mediated endocytosis.^19^ Thus, 13b behaves like the 5P12-RANTES derivative (Fig. 5F), in that it does not induce CCR5 phosphorylation, but unlike 5P12-RANTES^20^ it can still trigger CCR5 endocytosis. This demonstrates that the installation of macrocycles at the *N*-terminus of CCL5, with subtle functional group changes, is enough to decouple CCR5 phosphorylation and down-modulation requirements and constitutes a novel approach for dissecting chemokine ligand–receptor interactions.^24^ CCL5-ring expanded macrocycles therefore have the potential for future application in the removal of CCR5 from the cell-surface without inducing detrimental receptor signaling stimulation, as observed with earlier derivatives.^23^

## Conclusions

In conclusion, a novel strategy for N-terminal protein bioconjugation has been established using easy-to-prepare acryloyl imide reagents. The acryloyl imides reagents react selectively with N-terminal amines in a CARE cascade reaction on a range of biologically relevant proteins, with the CARE approach also validated using a range of amino acid derivatives and 6-mer peptide models. Selectivity for N-terminal modification over lysine side chains is observed, driven by differences in p*K*_a_ between α-amines and ε-amines. The ring expanded bioconjugates formed have enhanced stability, relative to conjugates formed using more established Michael acceptor reagents, driven by the irreversible ring expansion step in the CARE cascade. Importantly the CARE approach enables access to novel protein-macrocycle bioconjugates with broad scope for the incorporation of varied chemical functionality within the newly formed macrocycle. The utility of the approach is demonstrated in the modification of untagged native proteins, including nanobodies for cancer cell and tissue imaging, and human chemokines with altered downstream activation and processing. Thus, the CARE approach has significant potential to serve as a platform technology for the post-translational late-stage macrocycle derivatization and diversification of chemokines and other proteins.

## Ethical statement

All surgical specimens were collected in accordance with local ethical and regulatory guidelines, with written informed consent from patients (Newcastle REC 2003/11 and Human Tissue Authority License 12534, Freeman Hospital, Newcastle upon Tyne, United Kingdom).

## Author contributions

M. A. F., C. D. S and W. P. U. designed and supervised the study. O. R. H., A. T., E. H., J. N., R. H., and N. D. Y. performed the experiments and interpreted the results. A. C. W collected and processed the X-ray crystallographic data. C. N. R. and N. S. supervised and advised on biological experiments. O. R. H. M. A. F., C. D. S. and W. P. U. prepared the manuscript, supported by all authors.

## Conflicts of interest

There are no conflicts to declare.

## Supplementary Material

SC-OLF-D5SC08044D-s001

SC-OLF-D5SC08044D-s002

## Data Availability

CCDC 2391160 contains the supplementary crystallographic data for this paper.^10^ The data that support the findings of this study are available in the supplementary information (SI) of this article. Supplementary information: including detailed experimental procedures for small molecule, peptide and protein transformations, compound characterization data, chromatograms, LCMS data, MALDI, kinetic methods, protein expression methods, immunofluorescence staining and microscopy and NMR spectra images. See DOI: https://doi.org/10.1039/d5sc08044d.
