# Supplementary material for: N-terminal protein-macrocycles enabled by conjugate addition/ring expansion cascade reactions
Source: Chem Sci. 2025 Dec 16;17(6):3320–8. doi: 10.1039/d5sc08044d (PMC12720929; doi:10.1039/d5sc08044d)
Supplement: SC-017-D5SC08044D-s001 [file SC-017-D5SC08044D-s001.pdf]

Supplementary Information (SI) for Chemical Science.
This journal is © The Royal Society of Chemistry 2025

Table of contents:

SI1. General considerations

SI2. Reagent synthesis

SI3. Compound characterisation data and synthetic procedures

SI4. Peptide synthesis

SI5. Peptide modification: general procedures and materials

SI6. Peptide modification: supplementary figures

SI7. Protein expression

SI8. Protein modification: general procedures and materials

SI9. Protein modification: supplementary figures

SI10. Methods for functional testing of modified proteins

SI11. NMR spectra

SI12. References

SI1. General considerations

Unless otherwise stated, all chemical reagents were purchased and used directly from commercial sources. Anhydrous CH_2Cl_2 and THF were taken from an Innovative Technology Inc. Puresolv® solvent purification system and used without additional drying. Anhydrous MeOH was purchased from a commercial source and used without additional drying. Water used in reactions and protein manipulations was deionized. RNase A from bovine pancreas (powder, 50 units/mg protein), myoglobin from equine skeletal muscle (powder, 95–100%) and cytochrome C from bovine heart (powder, 95–100%) were purchased from Sigma Aldrich. ^{1}H NMR and ^{13}C NMR spectra were recorded on a JEOL ECX400 or JEOL ECS400 spectrometer (operating at 400 MHz and 100 MHz), a Bruker Avance III 300 NMR spectrometer (operating at 300 MHz, 75 MHz, and 282 MHz), a Bruker Avance I 500 MHz spectrometer (operating at 500 MHz, 125 MHz, and 470 MHz), a Bruker Avance III HD 500 NMR spectrometer (operating at 500 MHz, 125 MHz, and 470 MHz), a Bruker Avance III HD 600 NMR spectrometer (operating at 600 MHz, 151 MHz, and 565 MHz), or a Bruker Avance Neo 700 NMR spectrometer (operating at 700 MHz, 176 MHz, and 659 MHz). All spectral data was recorded at 295 K unless otherwise stated. All chemical shifts are quoted on the δ scale in ppm using residual solvent as the internal standard (^{1}H NMR: $CDCl_3$ = 7.26; CD_3OD = 3.31; D_2O = 4.69; DMSO-d_6 = 2.50 and ^{13}C NMR: $CDCl_3$ = 77.16, CD_3OD = 49.00, DMSO-d_6 – 39.52). Coupling constants (*J*) are reported in Hertz (Hz) to the nearest 0.5 Hz. The multiplicity abbreviations used are: br s broad singlet, s singlet, d doublet, br d broad doublet, t triplet, br t broad triplet, q quartet, p pentet, dd doublet of doublets, ddd doublet of doublets of doublets, dddd doublet of doublet of doublet of doublets, dt doublet of triplets, ddt doublet of triplets, td triplet of doublets, m multiplet. Signal assignment was achieved by analysis of 2D NMR techniques (DEPT, COSY, HMBC, HSQC) where required. Infrared (IR) spectra were recorded on a PerkinElmer UATR 2 spectrometer as a thin film dispersed from either CH_2Cl_2 or $CDCl_3$. Mass spectra (high-resolution) were obtained by the University of York Mass Spectrometry Service, using Electrospray Ionisation (ESI) on a Bruker Daltonics, Micro-tof spectrometer. Nominal and exact *m/z* values are reported in Daltons (Da). Specific rotations ($[\alpha]_D$) were recorded on a Bellingham + Stanley ADP450 polarimeter as a solution in $CDCl_3$. Melting points were determined using Gallenkemp apparatus. Thin layer chromatography was carried out on Merck silica gel $60F_{254}$ pre-coated aluminium foil sheets and were visualised using UV light (254 nm) and stained with basic aqueous potassium permanganate. In most cases, flash column chromatography was carried out using slurry packed Fluka silica gel (SiO_2), 35 – 70 µM, 60 Å, under a light positive pressure, eluting with the specified solvent system. In selected cases, products were purified using automated column chromatography; this was done using a Teledyne ISCO NextGen 300+ automated flash column chromatography unit equipped with UV–Vis (200–800 nm) and evaporative light scattering (ELS) detectors. Crude materials were loaded onto pre-packed RediSep Rf Gold columns (SiO_2: 40–60 mesh) or RediSep Gold® C18 reversed phase columns (SiO_2: 400–632 mesh) either by direct liquid injection or dry loading from adsorbed

Celite. X-ray diffraction data were collected at 110 K on an Oxford Diffraction SuperNova diffractometer with Cu-K_{α} radiation (λ = 1.54184 Å) using an EOS CCD camera. The crystal was cooled with an Oxford Instruments Cryojet. Diffractometer control, data collection, initial unit cell determination, frame integration and unit-cell refinement were carried out with CrysAlisPro (Rigaku Ltd). Face-indexed absorption corrections were applied using spherical harmonics, implemented in SCALE3 ABSPACK scaling algorithm within CrysAliPro. OLEX2 (J. Appl. Cryst., 2009, 42, 339–341) was used for overall structure solution, refinement and preparation of computer graphics and publication data. Within OLEX2, the algorithms used for structure solution was ShelXT dual-space. (G. M. Sheldrick, Acta Cryst. 2015, A71, 3-8). Refinement by full-matric least-squares used the SHELXL algorithm (G. M. Sheldrick, Acta Cryst. 2015, A71, 3-8) within OLEX2. Liquid chromatography-mass spectrometry (LC-MS) was performed on a HCTultra ETD II ion trap spectrometer, coupled to an Ultimate300 HPLC using an Accucore™ C18 column (150 x 2.1 mm, 2.6 μm particle size) or an Accucore™ 150-C4 column (100 x 2.1 mm, 2.6 μm particle size). Water (solvent A) and acetonitrile (solvent B), both containing 0.1% formic acid, were used as the mobile phase at a flow rate of 0.3 mL min^{-1}. LC traces were measured via UV absorption at 220, 270 and 280 nm. Peptide samples were eluted with a linear gradient 5–90% (increasing solvent B) over 13 minutes. Protein samples were eluted with a linear gradient 10–90% (increasing solvent B) over 15 minutes. Samples were analysed using the Bruker Data Analysis 4.4 software. Spectra were charge deconvoluted using ESI Compass 1.3. Both the raw ion series MS data and the deconvoluted spectra are shown for each sample. Expected masses were calculated relative to reported values of 13681 Da (RNase A),[1] 16951 Da (myoglobin),[2] 12327 Da (Cytochrome C), and 12124 (JVZ-007)[3]. Observed masses of CjX183-D WT and R51K mutant were typically ca. 10 Da higher than theoretical masses (11226 Da and 11198 Da respectively), so expected masses for CjX183-D were calculated from the smallest species present in each individual sample.

SI2. Reagent synthesis

Imide 6a:

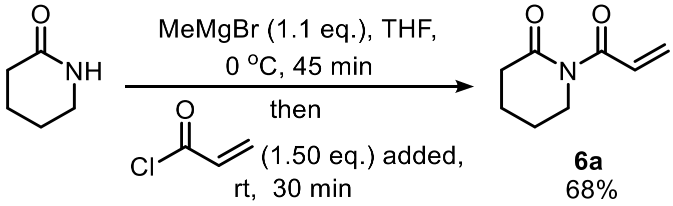

Imide 6b:

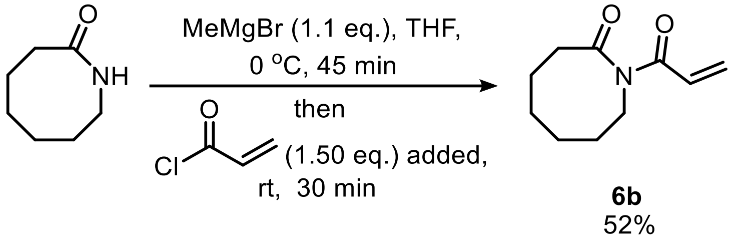

Imide 6c:

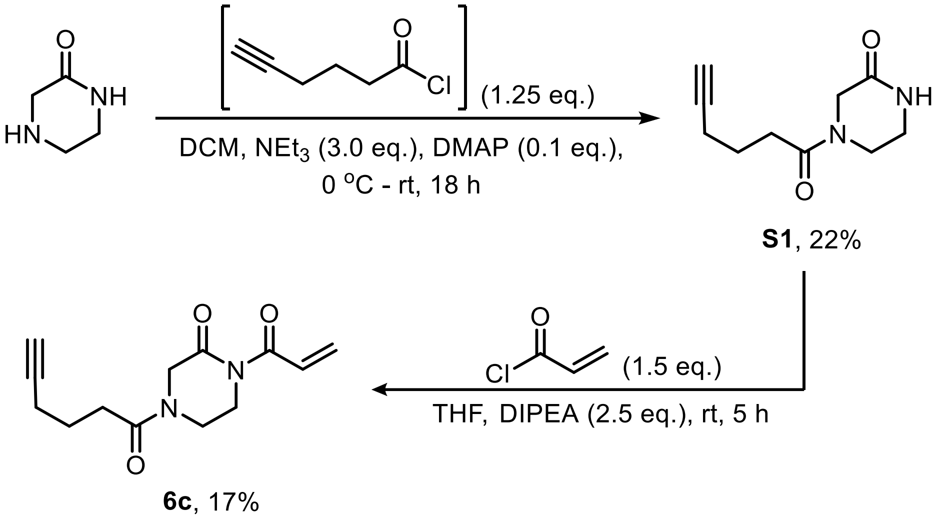

Scheme S1: Synthetic routes to acryloyl imides **6a**, **6b** and **6c** for CARE reactions.

SI3. Compound characterization data and synthetic procedures

Acryloyl-piperidin-2-one (6a)

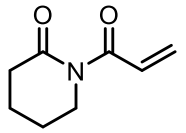

δ-Valerolactam (2.04 g, 20.6 mmol, 1.00 eq.) in dry THF (70 mL) was cooled to 0 °C before the dropwise addition of MeMgBr (3.0 M in Et_2O, 7.40 mL, 22.6 mmol, 1.10 eq.) over 30 mins using a syringe pump. After the addition was complete, the reaction mixture was stirred at 0 °C for 15 mins. At this point, acryloyl chloride (2.50 mL, 30.7 mmol, 1.50 eq.) was added as a single portion and the reaction was stirred for 1 h at 0 °C. The reaction was then quenched with sat. aq. NH_4Cl (60 mL), the aqueous mixture extracted with Et_2O (100 mL) and the organic extracts washed with sat. $NaHCO_3$ (2 x 60 mL). The organic phase was dried over $MgSO_4$ and concentrated *in vacuo*. The crude material was purified by FCC (SiO_2, 4:1; hexane: ethyl acetate) to afford the *title compound* as a viscous, colourless oil (2.13 g, 68%); R_f = 0.31 (4:1; hexane: ethyl acetate); ν_{max}/cm^{-1} (thin film) 2950, 1677, 1542, 1384, 1289, 1207, 1154, 795, 568; δ_H (400 MHz, $CDCl_3$) 7.23 (dd, *J* = 16.9, 9.5 Hz, 1H, COC**H**=CH_2), 6.58 (dd, *J* = 16.9, 1.7 Hz, 1H, COCH=C**H**H'), 5.94 (dd, *J* = 9.5, 1.7 Hz, 1H, COCH=CH**H'**), 4.05 – 3.93 (m, 2H, C**H**$_2$NCO), 2.88 – 2.77 (m, 2H, C**H$_2$**CON), 2.18 – 2.04 (m, 4H, 2 x C**H$_2$**); δ_C (101 MHz, $CDCl_3$) 173.8 (**C**O), 169.6 (**C**O), 132.0 (**C**HCH_2), 127.9 (**C**HCH_2), 44.6 (**C**H$_2$NCO), 34.9 (**C**H$_2$CON), 22.7 (**C**H$_2$), 20.7 (**C**H$_2$); HRMS (ESI$^+$): calcd. for $C_8H_{11}NNaO_2$, 176.0682. Found $[M+Na]^+$, 176.0683. The data obtained match those previously reported.[1]

1-Acryloylazocan-2-one (6b)

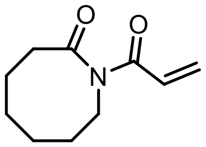

1-Aza-2-cyclooctanone (500 mg, 3.93 mmol, 1.00 eq.) in dry THF (20 mL) was cooled to 0 °C before the dropwise addition of MeMgBr (3.0 M in Et_2O, 1.43 mL, 4.32 mmol, 1.10 eq.) over 30 mins using a syringe pump. After complete addition, the reaction mixture was stirred at 0 °C for 15 mins. At this point, acryloyl chloride (0.48 mL, 5.90 mmol, 1.50 eq.) was added as a single portion and the reaction was stirred for 1 h at 0 °C. The reaction was then quenched with sat. aq. NH_4Cl (20 mL), the aqueous mixture extracted with Et_2O (30 mL) and the organic extracts washed with sat. $NaHCO_3$ (2 x 30 mL). The organic phase was dried over $MgSO_4$ and concentrated *in vacuo* to give the crude material as a yellow oil. Purification by FCC (SiO_2, 4:1; hexane: ethyl acetate) gave the *title compound* as low-melting point, colourless crystals (366 mg, 52%); M.P. = 29 – 33 °C; R_f = 0.15 (4:1; hexane: ethyl acetate); δ_H (400 MHz, $CDCl_3$) 6.88 (dd, *J* = 16.6, 10.5 Hz, 1H, COC**H**=CH_2), 6.31 (dd, *J* = 16.6, 1.7 Hz, 1H, COCH=C**H**H'), 5.68 (dd, *J* = 10.5, 1.7 Hz, 1H, COCH=CH**H'**), 3.96 – 3.89 (m, 2H, C**H$_2$**NCO),

1.95 – 1.84 (m, 2H, C$\mathbf{H_2}$CO), 1.77 (p, J = 6.2 Hz, 2H, C$\mathbf{H_2}$), 1.80 – 1.65 (m, 2H, C$\mathbf{H_2}$), 1.66 – 1.53 (m, 2H, C$\mathbf{H_2}$), 1.51 – 1.43 (m, 2H, C$\mathbf{H_2}$); δ_C (101 MHz, $CDCl_3$) 178.9 (**C**O), 169.4 (**C**O), 132.0 (CO**C**HCH_2), 127.7 (COCH**C**H_2), 43.8 (**C**H_2NCO), 36.7 (C$\mathbf{H_2}$CO), 30.0 (**C**H_2), 29.3 (**C**H_2), 26.3 (**C**H_2), 24.0 (**C**H_2). The data obtained match those previously reported.[1]

4-(Hex-5-ynoyl)piperazin-2-one (S1)

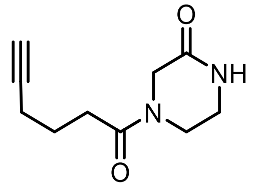

A solution of 5-hexynoic acid (0.12 mL, 1.09 mmol, 1.10 eq.), propylphosphonic anhydride (T3P, 0.95 mL, 1.49 mmol, 1.50 eq.) and *N,N'*-diisopropylethylamine (0.52 mL, 3.00 mmol, 3.00 eq.) in anhydrous DCM (18 mL) was stirred at rt for 10 minutes before the addition of piperazin-2-one (100 mg, 0.990 mmol, 1.00 eq.). The reaction mixture was stirred under these conditions for 18 h before being diluted with DCM (20 mL) and washed with sat. aq. $NaHCO_3$ (2 x 20 mL) and brine (2 x 20 mL). The organic extracts were dried over $MgSO_4$ and concentrated *in vacuo* to give the crude product which was purified by FCC (SiO_2, 19:1; ethyl acetate: methanol) to afford the *title compound* as a colourless oil (41.9 mg, 22%). In solution in $CDCl_3$, the compound exists as a mixture of two rotamers in an approximately 5:4 (A:B) ratio; R_f = 0.17 (19:1; ethyl acetate: methanol); ν_{max}/cm^{-1} (thin film) 3250, 2923, 2114, 1671, 1633, 1492, 1435, 1338, 1237, 1110, 982, 653, 536, 482; δ_H (400 MHz, $CDCl_3$) 7.59 (s, 1H, N**H** (B)), 7.30 (s, 1H, N**H** (A)), 4.21 (s, 2H, C$\mathbf{H_2}$CONH (B)), 4.13 (s, 2H, C$\mathbf{H_2}$CONH (A)), 3.79 (t, J = 5.5 Hz, 2H, CONHCH_2C$\mathbf{H_2}$ (A)), 3.68 (t, J = 5.3 Hz, 2H, CONHCH_2C$\mathbf{H_2}$ (B)), 3.44 – 3.39 (m, 2H, CONHC$\mathbf{H_2}$$CH_2$ (B)), 3.38 – 3.29 (m, 2H, CONHC$\mathbf{H_2}$$CH_2$ (A)), 2.53 – 2.40 (m, 4H, NCOC$\mathbf{H_2}$ (A,B [overlapping]), 2.32 – 2.21 (m, 4H, CH_2C$\mathbf{H_2}$CCH (A,B [overlapping]), 1.99 – 1.95 (m, 2H, CH_2CH_2CC**H** (A,B [overlapping]), 1.91 – 1.77 (m, 4H, C$\mathbf{H_2}$$CH_2$CCH (A,B [overlapping]); δ_C (101 MHz, $CDCl_3$) 171.0 (**C**O (A)), 170.8 (**C**O (B)), 168.6 (**C**O (B)), 167.3 (**C**O (A)), 83.6 (CH_2CH_2**C**CH (B)), 83.5 (CH_2CH_2**C**CH (A)), 69.5 (CH_2CH_2C**C**H (A)), 69.4 (CH_2CH_2C**C**H (B)), 48.7 (**C**H_2CONH (B)), 46.0 (**C**H_2CONH (A)), 42.3 (CONHCH_2**C**H_2 (B)), 41.2 (CONH**C**H_2CH_2 (B)), 40.8 (CONH**C**H_2CH_2 (A)), 38.4 (CONHCH_2**C**H_2 (A), 31.6 (NCO**C**H_2 (A)), 31.5 (NCO**C**H_2 (B)), 23.5 (**C**H_2CH_2CCH (B)), 23.4 (**C**H_2CH_2CCH (A)), 17.9 (CH_2**C**H_2CCH (A,B [overlapping])); HRMS (ESI^+): calc. for $C_{10}H_{15}N_2NaO_2$, 217.0947. Found $[M+Na]^+$, 217.0948.

1-Acryloyl-4-(hex-5-ynoyl)piperazine-2-one (6c)

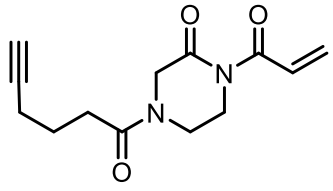

A solution of 4-(hex-5-ynol)piperazin-2-one **S1** (126 mg, 0.510 mmol, 1.00 eq.) in THF (2.50 mL) was cooled to 0 ºC before the addition of *N*, *N'*-diisopropylethylamine (0.220 mL, 1.27 mmol, 2.50 eq.). The mixture was stirred at this temperature for 10 minutes before the dropwise addition of acryloyl chloride (0.060 mL, 0.770 mmol, 1.50 eq.) in THF (0.50 mL). Upon complete addition, the reaction mixture was warmed to room temperature and stirring continued, monitored every hour by TLC until complete conversion of starting material was observed (~4 hours). The reaction mixture was then quenched with sat. aq. NH_4Cl (10 mL) and extracted with Et_2O (2 x 10 mL). The organic extracts were then washed with sat. aq. $NaHCO_3$ (2 x 10 mL) and brine (2 x 10 mL), dried over $MgSO_4$ and concentrated *in vacuo* to give the crude product. This was purified by FCC (SiO_2, 2:1; hexane: ethyl acetate → 1:1; hexane: ethyl acetate) to afford the *title compound* as a golden oil (21.3 mg, 17%). In solution in $CDCl_3$, the compound exists as a mixture of two rotamers in an approximately 4:3 (A:B) ratio; R_f = 0.19 (1:1; hexane: ethyl acetate); ν_{max}/cm^{-1} (thin film) 3280, 2933, 2115, 1686, 1647, 1434, 1407, 1390, 1366, 1307, 1191, 1111, 1028, 978, 965, 796, 641; δ_H (400 MHz, $CDCl_3$) 7.23 – 7.09 (m, 2H, COC**H**=CHH' (A, B [overlapping])), 6.50 – 6.40 (m, 2H, COCH=C**H**H' (A, B [overlapping])), 5.84 (d, *J* = 10.4 Hz, 1H, COCH=CH**H'** (A)), 5.83 (d, *J* = 10.4 Hz, 1H, COCHCH**H'** (B)), 4.40 (s, 2H, CONC**H₂**CO (A)), 4.32 (s, 2H, CONC**H₂**CO (B)), 4.04 – 3.96 (m, 2H, CONC**H₂**CH₂ (A)), 3.94 – 3.87 (m, 2H, CONC**H₂**CH₂ (B)), 3.84 – 3.77 (m, 2H, CONCH₂C**H₂** (B)), 3.77 – 3.71 (m, 2H, CONCH₂C**H₂** (A)), 2.54 – 2.43 (m, 4H, NCOC**H₂** (A, B [overlapping])), 2.30 (td, *J* = 6.7, 2.7 Hz, 4H, CH₂C**H₂**CCH (A, B [overlapping])), 2.04 – 1.94 (m, 2H, CH₂CH₂CC**H** (A, B [overlapping])), 1.88 (app p, *J* = 6.7 Hz, 4H, C**H₂**CH₂CCH (A, B [overlapping])); δ_C (101 MHz, $CDCl_3$) 171.1 (**C**O), 170.8 (**C**O), 168.5 (**C**O), 167.8 (**C**O), 167.7 (**C**O), 167.6 (**C**O), 130.9 (CO**C**HCHH' (A, B [overlapping])), 130.8 (COCH**C**HH' (A, B [overlapping])), 83.6 (CH₂CH₂**C**CH (A)), 83.5 (CH₂CH₂**C**CH (B)), 69.6 (CH₂CH₂C**C**H (B)), 69.5 (CH₂CH₂C**C**H (A)), 50.3 (CON**C**H₂CO (B)), 47.6 (CON**C**H₂CO (A)), 42.9 (CONCH₂**C**H₂ (A)), 41.9 (CON**C**H₂CH₂ (A, B [overlapping])), 39.8 (CONCH₂**C**H₂ (B)), 31.7 (NCO**C**H₂ (B)), 31.5 (NCO**C**H₂ (A)), 23.3 (**C**H₂CH₂CCH (A, B [overlapping]), 17.9 (CH₂**C**H₂CCH (A, B [overlapping]). HRMS (ESI$^+$): calc. for $C_{13}H_{16}N_2NaO_3$, 271.1053. Found $[M+Na]^+$, 271.1053.

Ethyl 2-(4,10-dioxo-1,5-diazecan-1-yl) acetate (7a)

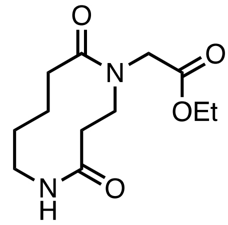

To a flask charged with glycine ethyl ester hydrochloride (206 mg, 1.48 mmol, 1.10 eq.) in triethylamine (0.740 mL, 5.24 mmol, 4.00 eq.) was added 1-acryloyl-piperidin-2-one **6a** (204 mg, 1.34 mmol, 1.00 eq.) as a solution in MeOH (1.50 mL). The solution was stirred at room temperature for 4 h before the solvent was removed *in vacuo* and the crude material purified by FCC (SiO_2, 19:1; ethyl acetate: methanol → 9:1; ethyl acetate: methanol). The *title compound* was isolated as a white powder (306 mg, 90%). M.P. = 117 – 120 ºC; R_f = 0.51 (100% MeOH); ν_{max}/cm^{-1} (thin film) 3316, 2931, 1721, 1640, 1557, 1477, 1208, 1153, 1022, 861, 716, 566, 523; δ_H (400 MHz, $CDCl_3$) 7.63 – 7.56 (m, 1H, N**H**), 4.74 (d, *J* = 17.3 Hz, 1H, C**H**H'COO), 4.30 – 4.16 (m, 2H, C**H₂**CH₃), 4.17 – 4.02 (m, 1H, C**H**H'), 3.88 – 3.74 (m, 1H, C**H**H'NH), 3.32 (d, *J* = 17.3 Hz, 1H, CH**H'**COO), 3.24 (dt, *J* = 15.6, 3.7 Hz, 1H, C**H**), 2.91 (dd, *J* = 13.8, 4.9 Hz, 1H, CH**H'**NH), 2.69 – 2.55 (m, 1H, C**H**H'), 2.41 (td, *J* = 12.8, 3.2 Hz, 1H, C**H**H'), 2.27 – 2.17 (m, 1H, C**H**H'), 2.14 – 1.98 (m, 2H, 2 x C**H**H'), 1.68 – 1.57 (m, 2H, 2 x C**H**H'), 1.53 – 1.38 (m, 1H, C**H**H'), 1.29 (t, *J* = 7.3 Hz, 3H, CH_2**CH_3**); δ_C (101 MHz, $CDCl_3$) 174.5 (**C**O), 172.3 (**C**O), 170.8 (**C**O), 62.2 (**C**H_2CH_3), 51.5 (**C**H_2COO), 48.9 (**C**H_2), 39.0 (**C**H_2NH), 37.5 (**C**H_2), 27.9 (**C**H_2), 25.2 (**C**H_2), 24.1 (**C**H_2), 14.1 (CH_2**C**H_3); HRMS (ESI^+): calc. for $C_{12}H_{20}N_2NaO_4$, 279.1315. Found $[M+Na]^+$, 279.1313. The data obtained match those previously reported.[2]

Ethyl 2-(5-acryloyl-4,10-dioxo-1,5-diazecan-1-yl)acetate (6d)

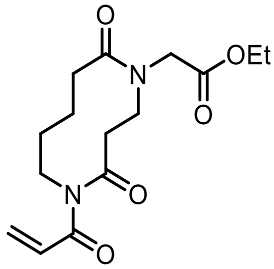

A stirring solution of ethyl 2-(4,10-dioxo-1,5-diazecan-1-yl) acetate **7a** (71.0 mg, 0.280 mmol, 1.00 eq.) and DIPEA (0.120 mL, 0.690 mmol, 2.50 eq.) in THF (1.70 mL) was cooled to 0 ºC. A 0 ºC cooled solution of acryloyl chloride (0.030 mL, 0.420 mmol, 1.50 eq.) in THF (0.50 mL) was added dropwise to the reaction mixture. The solution was stirred under an argon atmosphere for 2 h at 0 ºC before being warmed to rt and stirred for a further 17 h. The reaction mixture was then quenched with sat. aq. NH_4Cl (5 mL), extracted with Et_2O (2 x 5 mL), and the organic extracts washed with sat. aq. $NaHCO_3$ (2 x 5 mL). The combined organic mixture was dried over $MgSO_4$ and concentrated *in vacuo* to give the crude

material as a yellow oil. Purification through a silica plug (SiO_2, 1:1; hexane: ethyl acetate → 100% ethyl acetate) gave the *title compound* as a colourless oil (31.1 mg, 36%). R_f = 0.32 (100% ethyl acetate); ν_{max}/cm^{-1} (thin film) 2943, 1743, 1681, 1640, 1458, 1405, 1343, 1247, 1197, 1178, 1149, 1092, 1026, 983, 797; δ_H (400 MHz, $CDCl_3$) 6.66 (dd, *J* = 16.7, 10.3 Hz, 1H, COC**H**CHH'), 6.49 (dd, *J* = 16.7, 1.6 Hz, 1H, COCHC**H**H'), 5.91 (dd, *J* = 10.3, 1.6 Hz, 1H, COCHCH**H'**), 4.60 – 3.58 (m, 8H, COOC**H₂**CH₃, C**H₂**COO & 2 x C**H₂**), 3.27 (s, 2H, C**H₂**), 2.44 (s, 2H, C**H₂**), 1.91 (s, 2H, C**H₂**), 1.72 – 1.56 (m, 2H, C**H₂**), 1.25 (t, *J* = 7.0 Hz, 3H, COOCH₂C**H₃**); δ_C (101 MHz, $CDCl_3$) 173.3 (**C**O), 169.2 (**C**OO), 168.8 (**C**OCHCHH'), 132.2 (COCH**C**HH'), 129.4 (CO**C**HCHH'), 61.3 (COO**C**H₂CH₃), 47.4 (**C**H₂COO), 46.5 (**C**H₂), 45.7 (**C**H₂), 38.7 (**C**H₂), 29.0 (**C**H₂), 24.4 (**C**H₂), 24.2 (**C**H₂), 14.3 (COOCH₂**C**H₃); HRMS (ESI$^+$): calc. for $C_{15}H_{22}N_2NaO_5$, 333.1421. Found [M+Na]$^+$, 333.1424.

5-(4-Fluorobenzyl)-1,5-diazecane-2,6-dione (S2)

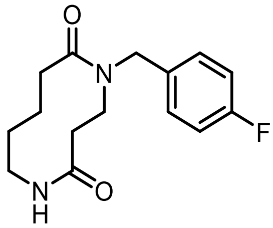

To a flask charged with 1-acryloyl-piperidin-2-one **6a** (496 mg, 3.24 mmol, 1.00 eq.) in methanol (7.50 mL) was added 4-fluorobenzylamine (0.410 mL, 3.57 mmol, 1.10 eq.). The mixture was stirred at room temperature for 4 h before the solvent was removed *in vacuo* and the crude material purified by FCC (SiO_2, 4:1; hexane: ethyl acetate → 2:1; hexane: ethyl acetate → 1:2; hexane: ethyl acetate → 1:19; methanol: ethyl acetate → 1:9; methanol: ethyl acetate). The *title compound* was obtained as a peach-coloured solid (610 mg, 68%). In solution in $CDCl_3$, the compound exists as a 10:1 (A:B) mixture of rotamers; R_f = 0.48 (9:1; ethyl acetate: methanol); ^{1}H NMR data for the major rotamer (A) can be found at: δ_H (400 MHz, $CDCl_3$) 7.34 – 7.24 (m, 2H, 2 x Ar**H**), 7.08 – 6.97 (m, 2H, 2 x Ar**H**), 5.17 (br d, *J* = 9.4 Hz, 1H, N**H**), 4.95 (d, *J* = 14.5 Hz, 1H, C**H**H'NCO), 4.29 (d, *J* = 14.7 Hz, 1H, CH**H'**NCO), 4.00 – 3.89 (m, 1H, C**H**), 3.88 – 3.75 (m, 1H, C**H**H'NH), 3.39 – 3.24 (m, 1H, C**H**), 3.00 – 2.80 (m, 1H, CH**H'**NH), 2.79 – 2.61 (m, 1H, C**H**), 2.28 – 2.03 (m, 4H, 4 x C**H**), 1.82 – 1.58 (m, 3H, 3 x C**H**); Diagnostic ^{1}H NMR signals for the minor rotamer (B) can be found at: δ_H (400 MHz, $CDCl_3$) 7.17 – 7.09 (m, 2H, 2 x Ar**H**), 5.91 (br d, *J* = 10.5 Hz, 1H, N**H**), 4.83 (d, *J* = 16.2 Hz, 1H, C**H**), 4.20 – 4.10 (m, 1H, C**H**), 2.47 – 2.30 (m, 1H, C**H**); ^{13}C NMR data for the major rotamer (A) can be found at: δ_C (101 MHz, $CDCl_3$) 174.2 (**C**O), 171.0 (**C**O), 162.4 (d, $^1J_{CF}$ = 246.5 Hz, Ar**C**F), 133.9 (d, $^4J_{CF}$ = 3.3 Hz, Ar**C**), 130.1 (d, $^3J_{CF}$ = 7.7 Hz, 2 x Ar**C**H), 115.9 (d, $^2J_{CF}$ = 21.1 Hz, 2 x Ar**C**H), 48.8 (**C**H₂NCO), 45.3 (**C**H₂), 39.4 (**C**H₂NH), 37.8 (**C**H₂), 28.4 (**C**H₂), 25.9 (**C**H₂), 23.9 (**C**H₂); Diagnostic ^{13}C NMR signals for the minor rotamer were not found; ^{19}F NMR data for the major rotamer (A) can be found at: δ_F (376

MHz, $CDCl_3$) -114.84 (m, 1F, Ar**F**); ^{19}F NMR data for the minor rotamer (B) can be found at: δ_F (376 MHz, $CDCl_3$) -114.42 (m, 1F, Ar**F**). The data obtained match those previously reported.[1,2]

1-Acryloyl-5-(4-fluorobenzyl)-1,5-diazecane-2,6-dione (6e)

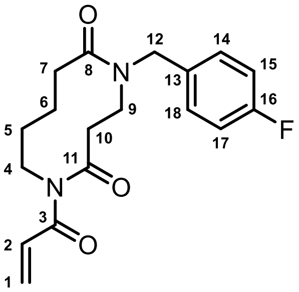

A stirring solution of 5-(4-fluorobenzyl)-1,5-diazecane-2,6-dione **S2** (100 mg, 0.36 mmol, 1.00 eq.) and DIPEA (0.16 mL, 0.90 mmol, 2.50 eq.) in THF (2.00 mL) was cooled to 0 ºC. A 0 ºC cooled solution of acryloyl chloride (0.04 mL, 0.54 mmol, 1.50 eq.) in THF (0.50 mL) was added dropwise to the reaction mixture. The solution was left stirring under an argon atmosphere for 2 h at this temperature and allowed to warm to rt whilst stirring for 18 h. The reaction mixture was then quenched with sat. aq. NH_4Cl (2 mL), extracted with Et_2O (2 x 5 mL), and the organic extracts washed with sat. aq. $NaHCO_3$ (2x 5 mL). The combined organic extracts were dried over $MgSO_4$ and concentrated *in vacuo* to give the crude material as a pale-yellow oil. Purification by FCC (SiO_2, 100 % ethyl acetate) gave the *title compound* as a pale-yellow oil (80.4 mg, 67%). In $CDCl_3$ the compound exists as a 10:1 (A:B) mixture of rotamers which causes significant broadening of the ^{1}H spectrum. Therefore, the compound's identity is most accurately determined using its ^{13}C NMR spectrum. R_f = 0.36 (100 % ethyl acetate); ^{1}H NMR data for the major rotamer (A) can be found at: δ_H (400 MHz, $CDCl_3$) 7.23 – 7.15 (m, 2H, 2 x Ar**H**), 7.07 – 6.91 (m, 2H, 2 x Ar**H**), 6.65 (dd, J = 16.6, 10.2 Hz, 1H, **H**-2), 6.47 (dd, J = 16.6, 1.5 Hz, 1H, **H**-1), 5.90 (dd, J = 10.2, 1.5 Hz, 1H, **H**-1'), 4.90 – 4.14 (m, 2H, C**H₂**), 3.89 (br s, 1H, C**H₂**), 3.60 (br s, 2H, C**H₂**), 3.29 (br s, 2H, C**H₂**), 2.43 (br s, 2H, C**H₂**), 1.94 (br s, 2H, C**H₂**), 1.76 – 1.66 (m, 2H, C**H₂**). Diagnostic ^{1}H NMR data for the minor rotamer (B) can be found at: 7.14 – 7.08 (m, 2H, 2 x Ar**H**), 6.81 – 6.64 (m, 1H), 6.41 (dd, J = 17.3, 1.6 Hz, 1H, **H**-1), 6.10 (dd, J = 17.3, 10.4 Hz, 1H, **H**-2), 5.85 (dd, J = 10.4, 1.6 Hz, 1H, **H**-1'), 4.79 (d, J = 16.2 Hz, 1H), 4.22 (d, J = 16.2 Hz, 1H). ^{13}C NMR data for the major rotamer (A) can be found at: δ_C (101 MHz, $CDCl_3$) 175.6 (**C**O), 173.0 (**C**O), 168.9 (**C**O), 162.3 (d, $^1J_{CF}$ = 245.4 Hz, **C**-16), 133.1 (d, $^4J_{CF}$ = 3.6 Hz, **C**-13), 132.2 (**C**-1), 130.0 (d, $^3J_{CF}$ = 8.5 Hz, **C**-14 & **C**-18), 129.5 (**C**-2), 115.6 (d, $^2J_{CF}$ = 21.0 Hz, **C**-15 & **C**-17), 46.9 (**C**H₂), 45.7 (**C**H₂), 43.9 (**C**H₂), 38.2 (**C**H₂), 29.2 (**C**H₂), 24.2 (**C**H₂), 24.1 (**C**H₂). Diagnostic ^{13}C NMR data for the minor rotamer (B) can be found at: 169.1 (**C**O), 168.3 (**C**O), 131.7, 128.5, 128.5 (d, $^3J_{CF}$ = 6.1 Hz, **C**-14 & **C**-18), 116.0 (d, $^2J_{CF}$ = 22.0 Hz, **C**-15 & **C**-17), 44.6 (**C**H₂); ^{19}F NMR data for the major rotamer (A) can be found at: δ_F (376 MHz, $CDCl_3$) -114.89 (m, 1F, Ar**F**); ^{19}F NMR data for the minor rotamer (B) can be found

at: δ_F (376 MHz, $CDCl_3$) -114.19 (m, 1F, Ar**F**); HRMS (ESI$^+$): calc. for $C_{18}H_{21}FN_2NaO_3$, 355.1428. Found $[M+Na]^+$, 355.1430. The data obtained match those previously reported.[1]

Ethyl 2-(4,10-dioxo-1,5-diazecan-1-yl) propanoate (7b)

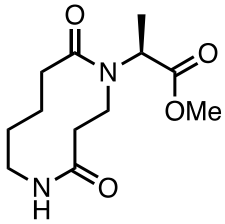

To a flask charged with *L*-alanine methyl ester hydrochloride (101 mg, 0.720 mmol, 1.10 eq.) in triethylamine (0.380 mL, 2.72 mmol, 4.00 eq.) was added 1-acryloyl-piperidin-2-one **6a** (104 mg, 0.680 mmol, 1.00 eq.) as a solution in MeOH (1.50 mL). The solution was stirred for 15 h before the solvent was removed *in vacuo* to afford the crude material as a white powder. This was purified by FCC using a gradient eluent system (SiO_2, 100% ethyl acetate → 9:1; ethyl acetate: methanol) to afford the *title compound* as a pale orange oil (70.9 mg, 41%). In $CDCl_3$ the compound exists as a 25:11 (A:B) mixture of rotamers. R_f = 0.22 (9:1; ethyl acetate: methanol); $[\alpha]_D^{20}$ = –11.06 (9.7 mg/mL in CH_2Cl_2); ν_{max}/cm^{-1} (thin film) 3322, 2934, 1717, 1667, 1633, 1552, 1431, 1348, 1214, 1256, 1211, 1156, 1102, 1060, 763, 734, 697. ^{1}H NMR signals for the major rotamer (A) can be found at: δ_H (400 MHz, $CDCl_3$) 7.39 – 7.32 (m, 1H, N**H**), 4.01 – 3.82 (m, 1H, NC**H**H'CH_2), 3.74 (s, 3H, $CH_3CHCOOC$**H$_3$**), 3.47 (q, *J* = 6.9 Hz, 1H, CH_3C**H**$COOCH_3$), 3.22 (dt, *J* = 15.5, 3.6 Hz, 1H, NCH**H'**CH_2), 2.89 – 2.80 (m, 2H, NHC**H$_2$**), 2.73 – 1.90 (m, 6H, NHCHH'C**H$_2$** & 2 x C**H$_2$** [overlapping with minor rotamer signals]), 1.51– 1.48 (m, 2H, C**H$_2$** [overlapping with minor rotamer signals]), 1.46 (d, *J* = 6.9 Hz, 3H, C**H$_3$**$CHCOOCH_3$). Diagnostic ^{1}H NMR signals for the minor rotamer (B) can be found at: δ 7.49 – 7.41 (m, 1H, N**H**), 4.88 (q, *J* = 7.9 Hz, 1H, CH_3C**H**$COOCH_3$), 3.71 (s, 3H, CHCOOC**H$_3$**), 3.42 – 3.39 (m, 2H, NC**H$_2$**CH_2), 2.73 – 1.90 (m, 6H, NHCHH'C**H$_2$** & 2 x C**H$_2$** [overlapping with major rotamer signals]), 1.51– 1.48 (m, 2H, C**H$_2$** [overlapping with major rotamer signals]), 1.40 (d, *J* = 7.9 Hz, 3H, C**H$_3$**$CHCOOCH_3$) – not all ^{1}H NMR signals for the minor rotamer (B) could be found. ^{13}C NMR signals for the major rotamer can be found at: δ_C (101 MHz, $CDCl_3$) 173.4 (**C**O), 173.2 (**C**O), 170.7 (**C**O), 58.7 (**C**HCOO), 52.8 (**C**H_3OCO), 47.4 (N**C**H_2), 39.0 (NH**C**H_2), 36.9 (**C**H_2), 27.9 (**C**H_2), 24.8 (**C**H_2), 23.8 (**C**H_2), 14.3 (**C**H_3CH). Diagnostic ^{13}C NMR signals for the minor rotamer can be found at: 176.2 (**C**O), 174.8 (**C**O), 170.9 (**C**O), 53.2 (**C**H_3OCO), 40.5 (**C**H_2), 38.9 (**C**H_2), 38.1 (**C**H_2), 28.5 (**C**H_2), 25.0 (**C**H_2), 23.9 (**C**H_2), 14.8 (**C**H_3CH); HRMS (ESI$^+$): calc. for $C_{12}H_{20}N_2NaO_4$, 279.1315. Found $[M+Na]^+$, 279.1316. The data obtained match those previously reported.[2]

Methyl 2-(4,10-dioxo-1,5-diazecan-1-yl)-3-methylbutanoate (7c)

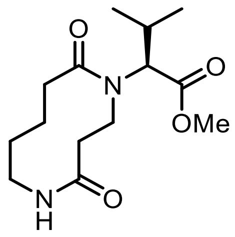

To a flask charged with *L*-valine methyl ester hydrochloride (128 mg, 0.720 mmol, 1.10 eq.) in triethylamine (0.370 mL, 2.68 mmol, 4.00 eq.) was added 1-acryloyl-piperidin-2-one **6a** (102 mg, 0.670 mmol, 1.00 eq.) as a solution in DCM (1.50 mL). Drops of methanol were added to the flask to aid the dissolution of the starting material. The solution was stirred for 23 h before the solvent was removed *in vacuo* to afford the crude material as a white powder. The crude material was then purified by FCC (SiO_2, 1:1; hexane: ethyl acetate → 1:2; hexane: ethyl acetate) to afford the *title compound* as a fine white powder (12.5 mg, 7%). R_f = 0.31 (100% ethyl acetate); $[\alpha]_D^{20}$ = –5.36 (10.0 mg/mL in CH_2Cl_2); ν_{max}/cm^{-1} (thin film) 2956, 1687, 1733, 1571, 1447, 1291, 1262, 1197, 1174, 1153, 1024, 912, 728, 646, 576; δ_H (400 MHz, $CDCl_3$) 3.81– 3.63 (m, 5H, COOC**H₃** & C**H₂**), 3.16 – 3.05 (m, 2H, C**H₂**), 3.01 (d, *J* = 6.1 Hz, 1H, C**H**COO), 2.95 – 2.87 (m, 1H, C**H**H'), 2.80 – 2.68 (m, 1H, C**H**H'), 2.59 – 2.49 (m, 2H, C**H₂**), 1.95 – 1.87 (m, 1H, C**H**(CH_3)₂), 1.87 – 1.75 (m, 4H, 2 x C**H₂**), 0.92 (t, *J* = 6.6 Hz, 6H, CH(C**H₃**)₂); δ_C (101 MHz, $CDCl_3$) 175.9 (**C**O), 175.5 (**C**O), 173.5 (**C**O), 67.8 (**C**HCOO), 51.6 (COO**C**H₃), 44.3 (**C**H₂), 44.0 (**C**H₂), 40.3 (**C**H₂), 35.0 (**C**H₂), 31.6 (**C**H(CH_3)₂), 22.5 (**C**H₂), 20.4 (**C**H₂), 19.2 (**C**H₃), 18.9 (**C**H₃); HRMS (ESI⁺): calc. for $C_{14}H_{24}N_2NaO_4$, 307.1628. Found $[M+Na]^+$, 307.1635.

Ethyl 2-(4,10-dioxo-1,5-diazecan-1-yl)-3-methylpentanoate (7d)

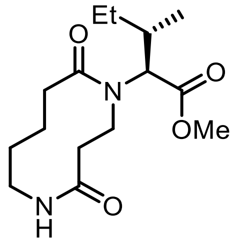

To a flask charged with methyl *L*-isoleucinate hydrochloride (130 mg, 0.720 mmol, 1.10 eq.) in triethylamine (0.360 mL, 2.60 mmol, 4.00 eq.) was added 1-acryloyl-piperidin-2-one **6a** (100 mg, 0.650 mmol, 1.00 eq.) as a solution in DCM (1.50 mL). The solution was stirred overnight for 23 h before being concentrated *in vacuo* to afford the crude material as a white paste. The crude material was purified by FCC using a gradient eluent system (2:1; hexane: ethyl acetate → 1:1; hexane: ethyl acetate → 2:1; ethyl acetate: hexane → 100% ethyl acetate) to give the *title compound* as a colourless oil (37.5 mg, 19%). R_f = 0.20 (2:1; ethyl acetate: hexane); $[\alpha]_D^{20}$ = 0.70 (10.0 mg/mL in CH_2Cl_2); ν_{max}/cm^{-1} (thin film) 2959, 2876, 1734, 1711, 1667, 1438, 1197, 1174, 730, 659; δ_H (400 MHz, $CDCl_3$) 3.73 – 3.68 (m,

5H, C**H₂** & C**H₃**OCO), 3.16 – 3.00 (m, 3H, C**H**COO & C**H₂**), 2.98 – 2.85 (m, 1H, C**H**H'), 2.78 – 2.67 (m, 1H, C**H**H'), 2.60 – 2.50 (m, 2H, C**H₂**), 1.89 – 1.76 (m, 4H, 2 x C**H₂**), 1.73 – 1.59 (m, 1H, C**H**CH_3), 1.56 – 1.44 (m, 1H, C**H**H'CH_3), 1.27 – 1.08 (m, 1H, CH**H'**CH_3), 0.97 – 0.84 (m, 6H, 2 x C**H₃**); δ_C (101 MHz, $CDCl_3$) 175.9 (**C**O), 175.6 (**C**O), 173.5 (**C**O), 66.6 (**C**HCOO), 51.5 (COO**C**H_3), 44.3 (**C**H_2), 44.0 (**C**H_2), 40.5 (**C**H_2), 38.3 (**C**HCH_3), 35.0 (**C**H_2), 25.8 (**C**H_2CH_3), 22.5 (**C**H_2), 20.4 (**C**H_2), 15.6 (**C**H_3), 11.6 (**C**H_3); HRMS (ESI$^+$): calc. for $C_{15}H_{26}N_2NaO_4$, 321.1785. Found $[M+Na]^+$, 321.1784.

Methyl 2-(4,10-dioxo-1,5-diazecan-1-yl)-4-(methylthio)butanoate (7e)

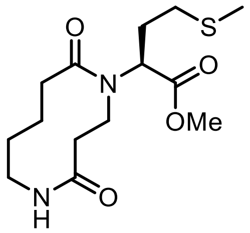

To a flask charged with *L*-methionine methyl ester hydrochloride (144 mg, 0.72 mmol, 1.10 eq.) in triethylamine (0.360 mL, 2.60 mmol, 4.00 eq.) was added 1-acryloyl-piperidin-2-one **6a** (100 mg, 0.650 mmol, 1.00 eq.) as a solution in MeOH (1.50 mL). The solution was stirred at room temperature for 16 h before being concentrated *in vacuo* to afford the crude material as an orange oil. The crude material was purified by FCC using a gradient eluent system (2:1; hexane: ethyl acetate → 1:1; hexane: ethyl acetate → 100% ethyl acetate → 9:1; ethyl acetate: methanol) to afford the *title compound* as a yellow oil (105 mg, 51%). In $CDCl_3$ the compound exists in a ~10:2:0.5 (A:B:C) mixture of rotamers. R_f = 0.64 (100% MeOH); $[\alpha]_D^{20}$ = –37.03 (10.0 mg/mL in CH_2Cl_2); ν_{max}/cm^{-1} (thin film) 3329, 2921, 2242, 1720, 1634, 1429, 1251, 908, 724, 645. ^{1}H NMR signals for the major rotamer (A) can be found at: δ_H (400 MHz, $CDCl_3$) 7.48 – 7.39 (m, 1H, N**H**), 3.99 – 3.88 (m, 1H, CONC**H**H'CHH'), 3.83 – 3.65 (m, 5H, C**H**COOCH₃, CHCOOC**H₃** & NHC**H**H'), 3.37 (br dt, *J* = 15.5, 3.7 Hz, 1H, CONCH**H'**CHH'), 2.87 – 2.78 (m, 1H, NHCH**H'**), 2.71 – 2.16 (m, 5H, CONCHH'C**H**H' & 4 x C**H**H'), 2.20 – 2.08 (m, 1H, CONCHH'CH**H'**), 2.06 – 1.91 (m, 6H, SC**H₃**, C**H₂** & C**H**H'), 1.61 – 1.30 (m, 3H, C**H₂** & CH**H'**). Diagnostic ^{1}H NMR signals for the minor rotamers can be found at: δ 7.24 – 7.17 (m, 1H, N**H** (B)), 6.56 (br t, *J* = 5.9 Hz, 1H, N**H** (C)), 3.64 (s, 3H, COOC**H₃** (C)), 3.56 (s, 3H, COOC**H₃** (B)) – not all ^{1}H NMR signals for the minor rotamers could be found, likely due to overlap with major rotamer signals. ^{13}C NMR signals for the major rotamer can be found at: δ_C (101 MHz, $CDCl_3$) 173.8 (**C**O), 173.2 (**C**O), 170.7 (**C**O), 61.0 (**C**HCOO), 52.9 (COO**C**H_3), 48.5 (CON**C**HH'CHH'), 38.8 (NH**C**HH'), 36.9 (CONCHH'**C**HH'), 30.8 (**C**H_2), 28.0 (**C**H_2), 27.6 (**C**H_2), 24.6 (**C**H_2), 23.5 (**C**H_2), 15.1 (S**C**H_3). Diagnostic ^{13}C NMR signals for the minor rotamers can be found at: 175.2 (**C**O), 175.0 (**C**O), 174.8 (**C**O), 172.4 (**C**O), 172.0 (**C**O), 170.9 (**C**O), 59.7, 55.9, 51.9 (COO**C**H_3 (C)), 51.5 (COO**C**H_3 (B)), 46.9, 44.0, 40.5, 38.6 , 37.6, 36.0, 35.0, 33.8, 33.4, 32.3, 30.4, 28.9, 25.0, 23.8, 22.1, 15.5, 15.2 – not all ^{13}C NMR signals could be found or assigned from rotamers B and C due to overlap with major rotamer signals; HRMS (ESI$^+$): Calcd. for $C_{14}H_{24}N_2NaO_4S$, 339.1349; Found $[M+Na]^+$, 339.1350.

Ethyl 2-(4,10-dioxo-1,5-diazecan-1-yl)-3-(4-hydroxyphenyl)propanoate (7f)

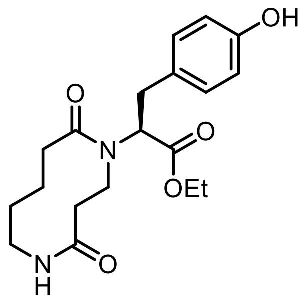

To a flask charged with *L*-tyrosine ethyl ester hydrochloride (357 mg, 1.45 mmol, 1.10 eq.) in triethylamine (0.750 mL, 5.40 mmol, 4.00 eq.) was added 1-acryloyl-piperidin-2-one **6a** (206 mg, 1.35 mmol, 1.00 eq.) as a solution in MeOH (3.00 mL). The solution was stirred for 15 h overnight before the crude material was purified by FCC (20:1; ethyl acetate: methanol) to afford the *title compound* as a pale orange oil (60.8 mg, 26%). R_f = 0.34 (15:1; ethyl acetate: methanol); ν_{max}/cm^{-1} (thin film) 3301, 2936, 2248, 1710, 1632, 1515, 1431, 1322, 1250, 1028, 907, 725, 645, 540; δ_H (400 MHz, $CDCl_3$) 7.65 – 7.58 (m, 1H, N**H**), 6.91 (d, *J* = 8.2 Hz, 2H, 2 x Ar**H**), 6.76 (d, *J* = 8.2 Hz, 2H, 2 x Ar**H**), 4.38 – 4.22 (m, 2H, COOC**H₂**CH₃), 3.88 – 3.75 (m, 1H, C**H**H'NH), 3.56 – 3.50 (m, 1H, C**H**COOEt), 3.50 – 3.44 (m, 1H, C**H**H'), 3.42 – 3.29 (m, 1H, C**H**H'ArC), 3.21 (dd, *J* = 13.8, 4.1 Hz, 1H, CH**H'**ArC), 3.03 – 2.78 (m, 1H, CH**H'**NH), 2.51 – 2.27 (m, 3H, 3 x C**H**H'), 2.15 – 1.98 (m, 3H, 3 x C**H**H'), 1.67 – 1.45 (m, 3H, 3 x C**H**H'), 1.35 (t, *J* = 7.1 Hz, 3H, COOCH₂C**H₃**); δ_C (101 MHz, $CDCl_3$) 174.0 (**C**O), 172.4 (**C**O), 171.6 (**C**O), 156.0 (Ar**C**OH), 130.2 (2 x Ar**C**H), 128.6 (Ar**C**), 115.7 (2 x Ar**C**H), 65.7 (**C**HCOOEt), 62.5 (**C**H₂CH₃), 48.6 (**C**H₂), 39.3 (**C**HH'NH), 36.4 (**C**H₂), 33.6 (**C**HH'ArC), 28.1 (**C**H₂), 24.7 (**C**H₂), 23.8 (**C**H₂), 14.1 (CH₂**C**H₃); HRMS (ESI$^+$): Calcd. for $C_{19}H_{26}N_2NaO_5$, 385.1734; Found $[M+Na]^+$, 385.1745

Methyl 2-(4,10-dioxo-1,5-diazecan-1-yl)-3-phenylpropanoate (7g)

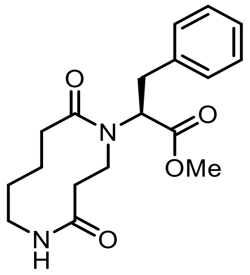

To a flask charged with *L*-phenylalanine methyl ester hydrochloride (157 mg, 0.720 mmol, 1.10 eq.) in triethylamine (0.360 mL, 2.60 mmol, 4.00 eq.) was added 1-acryloyl-piperidin-2-one **6a** (102 mg, 0.65 mmol, 1.00 eq.) as a solution in MeOH (1.50 mL). The solution was stirred for 4 h before being concentrated *in vacuo* and the crude material being purified by FCC (100% ethyl acetate) to afford the

title compound as a white solid (114 mg, 53%). In $CDCl_3$ the compound exists in a ~20:3 (A: B) mixture of rotamers. R_f = 0.16 (100% ethyl acetate); $[\alpha]_D^{20}$ = –119.34 (10.0 mg/mL in CH_2Cl_2); ν_{max}/cm^{-1} (thin film) 3328, 2932, 1717, 1668, 1553, 1454, 1319, 1257, 1043, 1019, 754, 702, 634. ^1H NMR signals for the major rotamer (A) can be found at: δ_H (400 MHz, $CDCl_3$) 7.36 – 7.01 (m, 5H, 5 x ArC**H**), 3.84 (s, 3H, CHCOOC**H₃**), 3.83 – 3.72 (m, 1H, CONC**H**H'), 3.62 – 3.35 (m, 3H, C**H**COOCH₃, CHC**H**H'ArC & C**H**H'), 3.30 (dd, *J* = 13.6, 4.3 Hz, 1H, CHCH**H'**ArC), 2.93 – 2.77 (m, 1H, CONCH**H'**), 2.52 – 2.37 (m, 1H, C**H**H'), 2.35 – 2.20 (m, 2H, 2 x C**H**H'), 2.13 – 1.93 (m, 3H, 3 x C**H**H'), 1.65 – 1.37 (m, 3H, C**H₂** & C**H**H'). Diagnostic ^1H NMR signals for the minor rotamers can be found at: δ 7.97 – 7.89 (m, 1H, N**H** (B)), 3.68 (s, 3H, COOC**H₃** (B)), 3.15 – 2.92 (m, 2H, C**H₂** (B)) – not all ^1H NMR signals for the minor rotamers could be found likely due to overlap with major rotamer signals. ^{13}C NMR signals for the major rotamer (A) can be found at: δ_C (101 MHz, $CDCl_3$) 173.9 (**C**O), 172.7 (**C**O), 170.7 (**C**O), 138.0 (Ar**C**), 129.3 (2 x Ar**C**H), 128.8 (2 x Ar**C**H), 126.9 (Ar**C**H), 65.3 (**C**HCOOCH₃), 53.1 (CHCOO**C**H₃), 48.6 (**C**H₂), 39.0 (**C**H₂), 36.7 (**C**H₂), 34.6 (CH**C**H₂ArC), 28.1 (**C**H₂), 24.9 (**C**H₂), 23.8 (**C**H₂). Diagnostic ^{13}C NMR signals for the minor rotamer were not found; HRMS (ESI⁺): Calcd. for $C_{18}H_{24}N_2NaO_4$, 355.1628; Found $[M+Na]^+$, 355.1635.

Methyl 2-(4,10-dioxo-1,5-diazecan-1-yl)-4-methylpentanoate (7h)

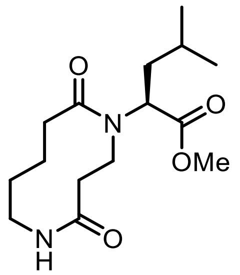

To a flask charged with *L*-leucine methyl ester hydrochloride (131 mg, 0.720 mmol, 1.10 eq.) in triethylamine (0.380 mL, 2.76 mmol, 4.00 eq.) was added 1-acryloyl-piperidin-2-one **6a** (106 mg, 0.690 mmol, 1.00 eq.) as a solution in MeOH (1.50 mL). The solution was stirred for 17 h before being concentrated *in vacuo* to afford the crude material as a white solid. The crude material was purified by FCC using a gradient eluent system (2:1; hexane: ethyl acetate → 1:1; hexane: ethyl acetate) to afford the *title compound* as a white solid (72 mg, 35%). In $CDCl_3$ the compound exists as a ~20:7:4 (A: B: C) mixture of rotamers. M.P. = 132 – 136 °C; R_f = 0.12 (100% ethyl acetate); $[\alpha]_D^{20}$ = –15.79 (10.0 mg/mL in CH_2Cl_2); ν_{max}/cm^{-1} (thin film) 3328, 2954, 2243, 1715, 1665, 1633, 1554, 1445, 1428, 1317, 1257, 1156, 912, 727, 643. ^1H NMR signals for the major rotamer (A) can be found at: δ_H (400 MHz, $CDCl_3$) 7.54 – 7.47 (m, 1H, N**H**), 4.11 – 3.97 (m, 1H, C**H**H'), 3.92 – 3.75 (m, 4H, C**H**H'NH & COOC**H₃** [overlapping with minor rotamer signal]), 3.45 (br dd, *J* = 8.9, 5.0 Hz, 1H, C**H**COO), 3.21 (dt, *J* = 15.5, 3.6 Hz, 1H, C**H**H'), 2.94 – 2.83 (m, 1H, CH**H'**NH), 2.61 – 2.36 (m, 2H, 2 x C**H**H' [overlapping with unidentifiable minor rotamer signals]), 2.22 (dd, *J* = 12.4, 3.9 Hz, 1H, C**H**H'), 2.12 – 1.86 (m, 3H,

$CH_2CH(CH_3)_2$ & CHH' [overlapping with unidentifiable minor rotamer signals]), 1.79 – 1.34 (m, 4H, CH_2, $CH_2CH(CH_3)_2$ & CHH' [overlapping with unidentifiable minor rotamer signals]), 0.97 – 0.87 (m, 6H, 2 x CH_3 [overlapping with minor rotamer signal]). Diagnostic ^{1}H NMR signals for the minor rotamers can be found at: δ 7.42 – 7.34 (m, 1H, NH (B)), 5.15 (dd, J = 10.6, 5.5 Hz, 1H, CHCOO (B)), 3.92 – 3.75 (m, 2H, CHH'NH (B) [overlapping with major rotamer signal]), 3.74 (s, 3H, $COOCH_3$ (B)), 3.68 (s, 2H, $COOCH_3$ (C)), 3.32 (dt, J = 16.2, 3.6 Hz, 1H, CHH'NH (B)), 2.75 – 2.65 (m, 1H, CHH' (B)), 2.18 – 2.13 (m, 1H, CHH' (B)), 0.97 – 0.87 (m, 6H, 2 x CH_3 (B & C) [overlapping with major rotamer signal]) - not all ^{1}H NMR signals for the minor rotamers could be found or identified likely due to overlap with major rotamer signals. ^{13}C NMR signals for the major rotamer (A) can be found at: δ_C (101 MHz, $CDCl_3$) 173.8 (CO), 173.5 ($COOCH_3$), 170.9 (CO), 61.9 (CHCOO), 53.0 ($COOCH_3$), 48.8 (CH_2), 39.1 (CH_2NH), 38.4 ($CH_2CH(CH_3)_2$), 37.0 (CH_2), 28.2 (CH_2), 25.0 ($CH(CH_3)_2$), 24.8 (CH_2), 23.7 (CH_2), 23.4 (CH_3), 22.3 (CH_3). Diagnostic ^{13}C NMR signals for the minor rotamers can be found at δ 176.0 (CO (B)), 175.4 (CO (B)), 171.2 (CO (B)), 55.1 (CHCOO (B)), 52.9 ($COOCH_3$ (B)), 52.0 ($COOCH_3$ (C)), 40.4 (CHH'NH (B)), 38.9 (CH_2 (B)), 37.8 (CH_2 (B)), 28.7 (CH_2 (B)), 25.2 (CH_2 (B)), 25.2 ($CH(CH_3)_2$ (B)), 24.0 (CH_2 (B)), 23.1 (CH_3 (B)), 23.0 (CH_3 (C)), 21.9 (CH_3 (C)), 21.4 (CH_3 (B)) – not all ^{13}C NMR signals for the minor rotamers could be found; HRMS (ESI$^+$): Calcd. for $C_{15}H_{26}N_2NaO_4$, 321.1785; Found $[M+Na]^+$, 321.1789.

Methyl 2-(4,10-dioxo-1,5-diazecan-1-yl)-2-hydroxybutanoate (7i)

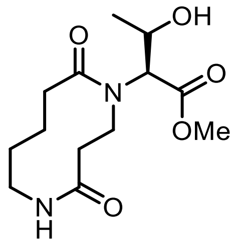

To a flask charged with *L*-threonine methyl ester hydrochloride (128 mg, 0.75 mmol, 1.10 eq.) in triethylamine (0.380 mL, 2.72 mmol, 4.00 eq.) was added 1-acryloyl-piperidin-2-one **6a** (104 mg, 0.680 mmol, 1.00 eq.) as a solution in MeOH (1.50 mL). The solution was stirred for 20 h before the solvent was removed *in vacuo* to afford the crude material as an orange oil. The crude material was purified by FCC using a gradient eluent system (2:1; ethyl acetate: hexane → 100% ethyl acetate → 9:1; ethyl acetate: methanol) to afford the *title compound* as a pale orange oil (36 mg, 19%). In $CDCl_3$ the compound exists as a ~9:1:1 (A: B: C) mixture of rotamers. R_f = 0.15 (9:1; ethyl acetate: methanol); $[\alpha]_D^{20}$ = –15.79 (10.0 mg/mL in CH_2Cl_2); ν_{max}/cm^{-1} (thin film) 3294, 2939, 1741, 1625, 1555, 1438, 1212, 1163, 728, 506. ^{1}H NMR signals for the major rotamer (A) can be found at: δ_H (400 MHz, $CDCl_3$) 7.49 – 7.42 (m, 1H, NH), 6.33 (s, 1H, OH), 4.53 – 4.43 (m, 1H, CH_3CHOH), 4.17 – 4.05 (m, 1H, CONCHH'CHH'), 3.93 – 3.78 (m, 4H, $COOCH_3$ & NHCHH'), 3.80 – 3.62 (m, 1H, CHCOOMe [overlapping with minor rotamer signals]), 3.34 – 3.18 (m, 1H, CONCHH'CHH' [overlapping with

minor rotamer signals]), 3.01 – 2.82 (m, 1H, NHCH**H'**), 2.74 – 2.57 (m, 1H, C**H**H'), 2.45 – 2.37 (m, 1H, CONCHH'C**H**H'), 2.28 – 1.97 (m, 3H, CONCHH'CH**H'** & 2 x C**H**H'), 1.68 – 1.41 (m, 3H, C**H**$_2$ & C**H**H'), 1.25 (d, J = 6.4 Hz, 3H, C**H**$_3$CHOH). Diagnostic ^{1}H NMR signals for the minor rotamers can be found at: δ 4.61 – 4.54 (m, 1H), 3.80 – 3.62 (m, 1H, COOC**H**$_3$ (B) & COOC**H**$_3$ (C) [overlapping with major rotamer signal]), 3.34 – 3.18 (m, 4H [overlapping with major rotamer signal]), 2.39 – 2.28 (m, 2H), 1.86 – 1.69 (m, 6H), 1.37 (d, J = 6.1 Hz, 3H, C**H**$_3$CHOH (B)), 1.20 (d, J = 6.1 Hz, 3H, C**H**$_3$CHOH (C)) - not all ^{1}H NMR signals for the minor rotamers were found due to overlap with major rotamer and impurity signals. ^{13}C NMR signals for the major rotamer (A) can be found at: δ_C (101 MHz, $CDCl_3$) 176.5 (**C**O), 172.5 (**C**O), 171.5 (**C**O), 69.0 (**C**HCOOMe), 66.7 (**C**HOH), 53.2 (COO**C**H$_3$), 49.5 (CON**C**HH'CHH'), 39.1 (NH**C**HH'), 36.8 (CONCHH'**C**HH'), 28.5 (**C**H$_2$), 24.9 (**C**H$_2$), 23.8 (**C**H$_2$), 21.2 (**C**H$_3$CHOH). Diagnostic ^{13}C NMR signals for the minor rotamers can be found at δ 170.6, 68.1, 67.8, 65.2, 52.6 (COO**C**H$_3$ (B)), 52.2, 51.7 (COOCH$_3$ (C)), 42.4, 44.6, 38.9, 37.3, 36.5, 33.5, 31.6, 29.0, 28.6, 25.5, 24.4, 22.4, 22.2, 20.9, 19.9 (**C**H$_3$CHOH) – not all ^{13}C NMR signals for the minor rotamers could be found or identified due to overlap with major rotamer and impurity signals; HRMS (ESI$^+$): Calcd. for $C_{13}H_{22}KN_2O_5$, 325.1160; Found $[M+K]^+$, 325.1158.

Methyl 2-(4,10-dioxo-1,5-diazecan-1-yl)-3-hydroxypropanoate (7j)

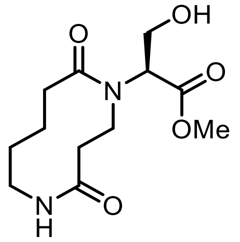

To a flask charged with *L*-serine methyl ester hydrochloride (113 mg, 0.726 mmol, 1.09 eq.) in triethylamine (0.370 mL, 2.64 mmol, 4.00 eq.) was added 1-acryloyl-piperidin-2-one **6a** (101 mg, 0.660 mmol, 1.00 eq.) as a solution in MeOH (1.50 mL). The solution was stirred for 17 h before the solvent was removed *in vacuo* to afford the crude material as a white powder. The crude material was purified by FCC using a gradient eluent system (2:1; hexane: ethyl acetate → 100% ethyl acetate → 20:1; ethyl acetate: methanol → 9:1; ethyl acetate: methanol) to afford the *title compound* as a colourless oil (83.4 mg, 47%). In $CDCl_3$ the compound exists as a 2:1:0.15 (A: B: C) mixture of rotamers. R_f = 0.33 (100% ethyl acetate); $[\alpha]_D^{20}$ = –6.63 (10.0 mg/mL in CH_2Cl_2); ν_{max}/cm^{-1} (thin film) 3315, 2935, 1731, 1632, 1564, 1433, 1209, 1036, 918, 726, 645. ^{1}H NMR signals for the major rotamer (A) can be found at: δ_H (400 MHz, $CDCl_3$) 7.31 – 7.22 (m, 1H, N**H**), 6.19 (s, 1H, O**H**), 4.15 (dd, J = 11.6, 5.1 Hz, 1H, C**H**H'OH), 4.09 – 3.94 (m, 2H, CH**H'**OH & C**H**H'), 3.87 – 3.59 (m, 5H, COOC**H**$_3$, C**H**COO & C**H**H'NH [overlapping with minor rotamer signals]), 3.44 – 3.34 (m, 1H, C**H**H'), 2.91 – 2.82 (m, 1H, CH**H'**NH [overlapping with unidentified minor rotamer signals]), 2.67 – 2.48 (m, 1H, C**H**H' [overlapping with minor rotamer signals]), 2.47 – 2.35 (m, 1H, C**H**H'), 2.23 – 2.16 (m, 1H, C**H**H'

[overlapping with minor rotamer signals]), 2.08 – 1.96 (m, 2H, 2 x C**H**H' [overlapping with minor rotamer signals]), 1.66 – 1.50 (m, 2H, 2 x C**H**H' [overlapping with minor rotamer signals]), 1.47 – 1.35 (m, 1H, C**H**H'). Diagnostic ^{1}H NMR signals for the minor rotamers can be found at: δ 8.30 – 8.24 (m, 1H, N**H** (C)), 7.52 – 7.45 (m, 1H, N**H** (B)), 4.37 – 4.31 (m, 2H, C**H**H'OH (B & C)), 4.08 – 4.02 (m, 1H, CH**H'**OH (B)), 3.87 – 3.59 (m, 6H, C**H**$_3$OCO (B & C) [overlapping with major rotamer signals]), 3.55 – 3.47 (m, 2H, 2 x C**H**H' (C)), 3.31 – 3.15 (m, 5H), 2.67 – 2.48 (m, 2H, C**H$_2$** (C) [overlapping with major rotamer signals]), 2.29 (t, J = 6.4 Hz, 2H, C**H$_2$** (B)), 1.81 – 1.65 (m, 4H, 2 x C**H$_2$** (B)) – not all ^{1}H NMR signals for the minor rotamers were found due to overlap with major rotamer and impurity signals. ^{13}C NMR signals for the major rotamer (A) can be found at: δ_C (101 MHz, CDCl$_3$) 174.8 (**C**O), 171.6 (**C**O), 171.0 (**C**O), 64.5 (**C**HCOO), 60.7 (**C**H$_2$OH), 52.8 (**C**H$_3$OCO), 48.5 (**C**H$_2$), 39.2 (**C**H$_2$NH), 37.0 (**C**H$_2$), 28.3 (**C**H$_2$), 25.0 (**C**H$_2$), 23.9 (**C**H$_2$). Diagnostic ^{13}C NMR signals for the minor rotamers can be found at: δ 175.1 (**C**O), 172.7 (**C**O), 171.7 (**C**O), 171.3 (**C**O), 64.3 (**C**HCOO), 63.0 (**C**H$_2$OH (C)), 62.9, 59.2 (**C**H$_2$OH (B)), 56.0, 52.5 (**C**H$_3$OCO (B/C)), 52.2 (**C**H$_3$OCO (B/C)), 45.7 (**C**H$_2$ (C)), 39.0 (**C**H$_2$NH), 42.4, 37.6 (**C**H$_2$), 33.6, 31.5 (**C**H$_2$ (B)), 28.8, 25.7, 24.5, 22.3 (**C**H$_2$ (B)), 20.9 (**C**H$_2$ (B)) – not all ^{13}C NMR signals for the minor rotamers could be found or identified due to overlap with major rotamer and impurity signals; HRMS (ESI^{+}): Calcd. for $C_{12}H_{20}N_2NaO_5$, 295.1264; Found [M+Na]$^{+}$, 295.1268.

Methyl 2-(4,10-dioxo-1,5-diazecan-1-yl)-3-(1*H*-imidazol-4-yl) propanoate (7k)

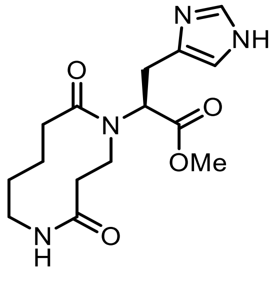

To a flask charged with *L*-histidine methyl ester dihydrochloride (174 mg, 0.72 mmol, 1.10 eq.) in triethylamine (0.360 mL, 2.60 mmol, 4.00 eq.) was added 1-acryloyl-piperidin-2-one **6a** (99 mg, 0.650 mmol, 1.00 eq.) as a solution in MeOH (1.50 mL). The solution was stirred for 18 h before contaminating quaternary ammonium salts were precipitated in acetone and filtered from the reaction mixture. The filtrate was concentrated *in vacuo* to give the crude material as an orange oil. This was purified by FCC (6:1; ethyl acetate: methanol) to afford the *title compound* as a white powder (57.1 mg, 27%). M.P. = 47 – 52 ºC; R_f = 0.18 (ethyl acetate: methanol); $[\alpha]_D^{20}$ = –5.12 (10.0 mg/mL in CDCl$_3$); ν_{max}/cm^{-1} (thin film) 3319, 2933, 1717, 1633, 1563, 1433, 1263, 729, 634; δ_H (400 MHz, CDCl$_3$) 7.65 – 7.54 (m, 2H, Ar**H** & N**H**), 6.77 (s, 1H, Ar**H**), 4.21 – 4.11 (m, 1H, C**H**COOCH$_3$), 3.89 – 3.71 (m, 4H, CHCOOC**H$_3$** & C**H**H'NH), 3.76 – 3.53 (m, 1H, C**H**H'), 3.48 – 3.29 (m, 2H, CHC**H$_2$**), 3.00 – 2.88 (m, 2H, CH**H'**NH & C**H**H'), 2.52 – 2.38 (m, 2H, 2 x C**H**H'), 2.15 – 1.92 (m, 3H, 3 x C**H**H'), 1.64 – 1.37 (m, 3H, C**H$_2$** & C**H**H'); δ_C (101 MHz, CDCl$_3$) 173.9 (**C**O), 173.0 (**C**O), 171.3 (**C**O), 136.2 (Ar**C**H),

135.0 (Ar**C**), 115.3 (Ar**C**H), 62.8 (**C**HCOO), 53.1 (COO**C**H_3), 48.0 (**C**H_2), 39.1 (**C**H_2NH), 37.0 (**C**H_2), 28.1 (**C**H_2), 27.0 (CH**C**H_2), 25.0 (**C**H_2), 24.0 (**C**H_2); HRMS (ESI$^+$): Calcd. for $C_{15}H_{22}N_4NaO_4$, 345.1533; Found [M+Na]$^+$, 345.1531.

Methyl 5-amino-2-(4,10-dioxo-1,5-diazecan-1-yl)-5-oxopentanoate (7l)

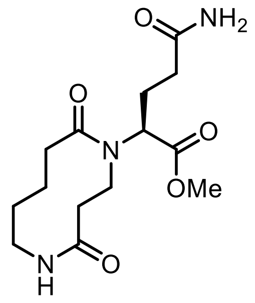

To a flask charged with glutamine methyl ester hydrochloride (141 mg, 0.72 mmol, 1.10 eq.) in triethylamine (0.370 mL, 2.68 mmol, 4.00 eq.) was added 1-acryloyl-piperidin-2-one **6a** (102 mg, 0.670 mmol, 1.00 eq.) as a solution in MeOH (1.50 mL). The solution was stirred for 18 h before the solvent was removed *in vacuo* to afford the crude material as a white powder. This was purified by FCC using a gradient eluent system (9:1; ethyl acetate: methanol → 6:1; ethyl acetate: methanol → 3:1; ethyl acetate: methanol) to afford the *title compound* as a colourless oil (64 mg, 31%). In $CDCl_3$ the compound exists as a 10:1 (A: B) mixture of rotamers. R_f = 0.21 (3:1; ethyl acetate: methanol); $[\alpha]_D^{20}$ –18.08 (10.0 mg/mL in CH_2Cl_2); ν_{max}/cm^{-1} (thin film) 3320, 3206, 2934, 1716, 1629, 1557, 1432, 1257, 1210, 1175, 1041, 920, 728, 646, 582. ^{1}H NMR signals for the major rotamer (A) can be found at: δ_H (400 MHz, $CDCl_3$) 7.49 – 7.42 (m, 1H, N**H**), 5.99 (s, 1H, CONH**H**'), 5.91 (s, 1H, CONH**H'**), 4.01 – 3.91 (m, 1H, C**H**H'), 3.86 – 3.67 (m, 5H, C**H**COO, COOC**H$_3$** & C**H**H'NH), 3.39 (dt, J = 15.5, 3.6 Hz, 1H, C**H**H'), 2.96 – 2.82 (m, 1H, CH**H'**NH), 2.63 – 2.12 (m, 7H, 3 x C**H$_2$** & C**H**H' [overlapping with unidentifiable minor rotamer signals]), 2.12 – 1.91 (m, 2H, 2 x C**H**H'), 1.66 – 1.34 (m, 2H, CH_2 & C**H**H'). Diagnostic ^{1}H NMR signals for the minor rotamers can be found at: δ 7.28 – 7.20 (m, 1H, N**H** (B)), 3.62 (s, 3H, COOC**H$_3$** (B)) - not all ^{1}H NMR signals for the minor rotamer (B) were found due to overlap with major rotamer signals and signal weakness. ^{13}C NMR signals for the major rotamer (A) can be found at: δ_C (101 MHz, $CDCl_3$) 175.2 (**C**O), 174.1 (**C**O), 173.2 (**C**O), 171.1 (**C**O), 62.0 (**C**HCOO), 53.0 (COO**C**H_3), 48.5 (**C**H_2), 39.0 (**C**HH'NH), 37.0 (**C**H_2), 31.2 (**C**H_2), 28.1 (**C**H_2), 24.8 (**C**H_2), 24.6 (**C**H_2), 23.7 (**C**H_2). Diagnostic ^{13}C NMR signals for the minor rotamer were not found. HRMS (ESI$^+$): Calcd. for $C_{14}H_{23}N_3NaO_5$, 336.1530; Found [M+Na]$^+$, 336.1527.

Diethyl 2-(4,10-dioxo-1,5-diazecan-1-yl)pentanedioate (7m)

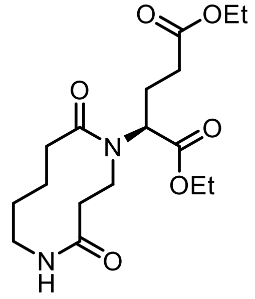

To a flask charged with glutamic acid diethyl ester hydrochloride (173 mg, 0.72 mmol, 1.10 eq.) in triethylamine (0.360 mL, 2.60 mmol, 4.00 eq.) was added 1-acryloyl-piperidin-2-one **6a** (100 mg, 0.650 mmol, 1.00 eq.) as a solution in MeOH (1.50 mL). The solution was stirred for 18 h before the solvent was removed *in vacuo* to afford the crude material as a white solid. The crude material was purified by FCC using a gradient eluent system (1:1; hexane: ethyl acetate → ethyl acetate) to afford the *title compound* as a colourless oil (85 mg, 37%) which in $CDCl_3$ exists as a complex mixture of rotamers, with the most distinguishable in a 12:1 (A: B) ratio with the major rotamer's signals. R_f = 0.12 (100% ethyl acetate); $[\alpha]_D^{20}$ –18.08 (10.0 mg/mL in $CDCl_3$); ν_{max}/cm^{-1} (thin film) 3323, 2981, 2934, 2240, 1717, 1668, 1639, 1446, 1254, 1181, 1023, 919, 727. ^{1}H NMR signals for the major rotamer (A) can be found at: δ_H (400 MHz, $CDCl_3$) 7.51 – 7.44 (m, 1H, N**H**), 4.28 – 4.13 (m, 2H, COOC**H₂**CH₃ [overlapping with minor rotamer signals]), 4.15 – 3.98 (m, 2H, COOC**H₂**CH₃ [overlapping with minor rotamer signals]), 3.99 – 3.90 (m, 1H, C**H**H'), 3.82 – 3.68 (m, 1H, C**H**H'NH), 3.63 – 3.56 (m, 1H, C**H**COOEt), 3.23 (dt, *J* = 15.4, 3.7 Hz, 1H, C**H**H'), 2.88 – 2.79 (m, 1H, CH**H'**NH), 2.56 – 2.21 (m, 6H, C**H**H'CHH'COOEt, CH**H'**CHH'COOEt, CHH'C**H**H'COOEt, CHH'CH**H'**COOEt & 2 x C**H**H'), 2.15 (br dd, *J* = 12.2, 3.8 Hz, 1H, C**H**H'), 2.05 – 1.89 (m, 2x C**H**H'), 1.60 – 1.34 (m, 3H, C**H₂** & C**H**H'), 1.26 (t, *J* = 7.1 Hz, 3H, C**H₃**), 1.18 (t, *J* = 7.1 Hz, 3H, C**H₃** [overlapping with minor rotamer signals]). Diagnostic ^{1}H NMR signals for the distinguishable minor rotamer (B) can be found at: δ 7.30 – 7.23 (m, 1H, N**H** (B)), 4.28 – 4.13 (m, 1H, COOC**H**H'CH₃ (B) & COOCH**H'**CH₃ (B) [overlapping with major rotamer signals]), 4.12 – 3.99 (m, 2H, COOC**H₂**CH₃ (B) [overlapping with major rotamer signals]), 3.44 – 3.34 (m, 1H, C**H**COOEt (B)), 1.81 – 1.68 (m, 1H, C**H**H' (B)), 1.24 – 1.12 (m, 6H, 2 x C**H₃** (B) [overlapping with major rotamer signals]) – due to the presence of a complex mixture of rotamers, not all the ^{1}H NMR signals for the distinguishable minor rotamer (B) or other minor rotamers could be found. ^{13}C NMR signals for the major rotamer (A) can be found at: δ_C (101 MHz, $CDCl_3$) 173.8 (**C**O), 173.3 (**C**O), 172.5 (**C**O), 170.8 (**C**O), 62.2 (COO**C**H₂CH₃), 62.1 (C**H**COOEt), 60.6 (COO**C**H₂CH₃), 48.5 (**C**H₂), 38.9 (**C**HH'NH), 36.9 (CHH'**C**HH'COOEt), 30.4 (**C**H₂), 27.9 (**C**H₂), 24.7 (**C**H₂), 24.3 (**C**HH'CHH'COOEt), 23.6 (**C**H₂), 14.2 (**C**H₃), 14.0 (**C**H₃). Diagnostic ^{13}C NMR signals for the distinguishable minor rotamer (B) can be found at δ 60.9 (COO**C**H₂CH₃), 60.4 (COO**C**H₂CH₃), 53.7 (**C**HCOOEt), 29.7 (**C**H₂), 14.1 (**C**H₃), 14.0 (**C**H₃). The remaining ^{13}C NMR signals for all minor

rotamers can be found at: 177.7, 176.0, 175.6, 175.3, 173.1, 173.1, 172.6, 172.0, 171.0, 61.5, 60.8, 56.4, 55.4, 55.2, 53.7, 52.5, 52.0, 40.5, 38.8, 37.7, 31.2, 30.6, 30.5, 29.7, 29.1, 28.5, 25.1, 24.8, 23.9, 23.3; HRMS (ESI$^+$): Calcd. for $C_{17}H_{28}N_2NaO_6$, 379.1840; Found $[M+Na]^+$, 379.1837.

Methyl 2-(4,10-dioxo-1,5-diazecan-1-yl)-3-(1*H*-indol-3-yl)propanoate (7n)

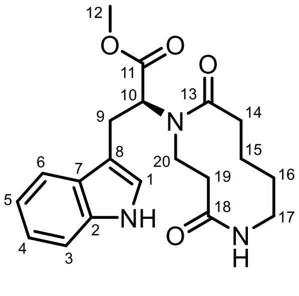

To a flask charged with tryptophan methyl ester hydrochloride (185 mg, 0.73 mmol, 1.10 eq.) in triethylamine (0.380 mL, 2.76 mmol, 4.00 eq.) was added 1-acryloyl-piperidin-2-one **6a** (106 mg, 0.690 mmol, 1.00 eq.) as a solution in MeOH (1.50 mL). The solution was stirred for 17 h before the solvent was removed *in vacuo* to afford the crude material as a white solid. Efforts to purify the *title compound* using FCC were unsuccessful due to co-elution with several other reaction by-products. However, the *title compound* was successfully purified through recrystallisation in $CDCl_3$ (28 mg, 11%). R_f = 0.38 (9:1; ethyl acetate: methanol); $[\alpha]_D^{20}$ –117.96 (10.0 mg/mL in $CDCl_3$); ν_{max}/cm^{-1} (thin film) 3309, 3181, 2944, 1719, 1627, 1434, 1250, 1029, 744, 724, 645; δ_H (400 MHz, $CDCl_3$) 8.10 (s, 1H, ArN**H**), 7.51 (d, *J* = 8.1 Hz, 1H, ArC**H**), 7.37 (d, *J* = 8.1 Hz, 1H, ArC**H**), 7.26 – 7.16 (m, 1H, ArC**H**), 7.13 – 7.08 (m, 1H, ArC**H**), 6.99 (br d, *J* = 2.3 Hz, 1H, **H**-1), 3.90 (s, 3H, **H**-12), 3.87 – 3.60 (m, 4H, **H**-10 & 3 x C**H**H'), 3.56 – 3.42 (m, 2H, **H**-9 & C**H**H'), 2.91 (d, *J* = 13.9 Hz, 1H, C**H**H'), 2.51 – 2.35 (m, 2H, **H**-9' & C**H**H'), 2.27 (td, *J* = 12.7, 3.1 Hz, 1H, C**H**H'), 2.15 – 2.06 (m, 2H, 2 x C**H**H'), 1.99 – 1.90 (m, 1H, C**H**H'), 1.67 – 1.45 (m, 2H, 2 x C**H**H'); δ_C (101 MHz, $CDCl_3$) 173.8 (**C**O), 173.0 (**C**O), 170.9 (**C**O), 136.3 (Ar**C**), 127.3 (Ar**C**), 122.9 (**C**-1), 122.5 (Ar**C**H), 119.9 (Ar**C**H), 118.3 (Ar**C**H), 112.2 (Ar**C**), 111.6 (Ar**C**H), 64.4 (**C**-10), 53.1 (**C**-12), 48.6 (**C**-9), 39.1 (**C**H_2), 36.8 (**C**H_2), 28.3 (**C**H_2), 25.0 (**C**H_2), 24.3 (**C**H_2), 23.9 (**C**H_2); HRMS (ESI$^+$): Calcd. for $C_{20}H_{26}N_3NaO_4$, 394.1737; Found $[M+Na]^+$, 394.1746.

Cysteine methyl ester:

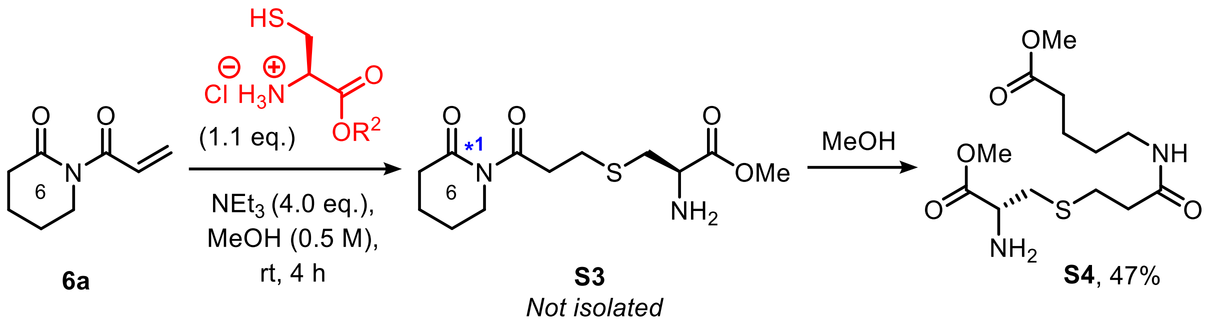

6a **S3** *Not isolated* **S4**, 47%

Methyl 5-(3-((2-amino-3-methoxy-3-oxopropyl)thio)propanamido)pentanoate (S4)

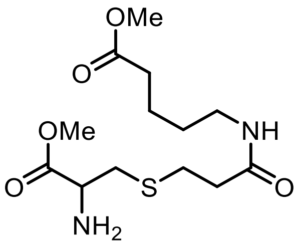

To a flask charged with cysteine methyl ester hydrochloride (124 mg, 0.76 mmol, 1.10 eq.) in triethylamine (0.400 mL, 2.76 mmol, 4.00 eq.) was added 1-acryloyl-piperidin-2-one **2.01** (107 mg, 0.690 mmol, 1.00 eq.) as a solution in MeOH (1.50 mL). The solution was stirred for 18 h before the solvent was removed *in vacuo* to afford the crude material as a colourless oil. The crude material was purified by FCC using a gradient eluent system (100% ethyl acetate → 9:1; ethyl acetate: methanol). The *title compound* was isolated as one of many reaction byproducts as a colourless oil (105 mg, 47%). R_f = 0.11 (9:1; ethyl acetate: methanol); ν_{max}/cm^{-1} (thin film) 3281, 2949, 2871, 1732, 1637, 1551, 1496, 1437, 1353, 1199, 1168, 1110, 660, 499; δ_H (400 MHz, $CDCl_3$) 6.83 (s, 2H, **N**H_2), 6.60 – 6.43 (m, 1H, N**H**), 3.67 (s, 3H, COOC**H**$_3$), 3.64 – 3.56 (m, 4H, CH_2CHCOOC**H**$_3$ & CH_2C**H**COOCH_3), 3.26 – 3.20 (m, 2H, C**H**$_2$), 3.17 (q, *J* = 6.5 Hz, 2H, C**H**$_2$), 2.95 – 2.80 (m, 1H, C**H**H'CHCOOCH_3), 2.81 – 2.67 (m, 3H, CH**H'**CHCOOCH_3 & C**H**$_2$), 2.38 (t, *J* = 7.2 Hz, 2H, C**H**$_2$), 2.30 – 2.22 (m, 1H, C**H**$_2$), 1.62 – 1.53 (m, 1H, C**H**), 1.53 – 1.39 (m, 1H, C**H**); δ_C (101 MHz, $CDCl_3$) δ 174.4 (**C**O), 174.0 (**C**O), 171.1 (**C**O), 54.1 (CH_2**C**HCOOCH_3), 52.3 (COO**C**H_3), 51.5 (CH_2CHCOO**C**H_3), 42.2 (**C**H_2), 39.0 (**C**H_2), 37.2 (**C**H_2CHCOOCH_3), 36.6 (**C**H_2), 31.4 (**C**H_2), 28.4 (**C**H_2), 22.1 (**C**H_2); HRMS (ESI$^+$): Calcd. for $C_{13}H_{25}N_2O_5S$, 321.1479; Found $[M+Na]^+$, 321.1482.

<u>Dimethyl guanidine sulphate:</u>

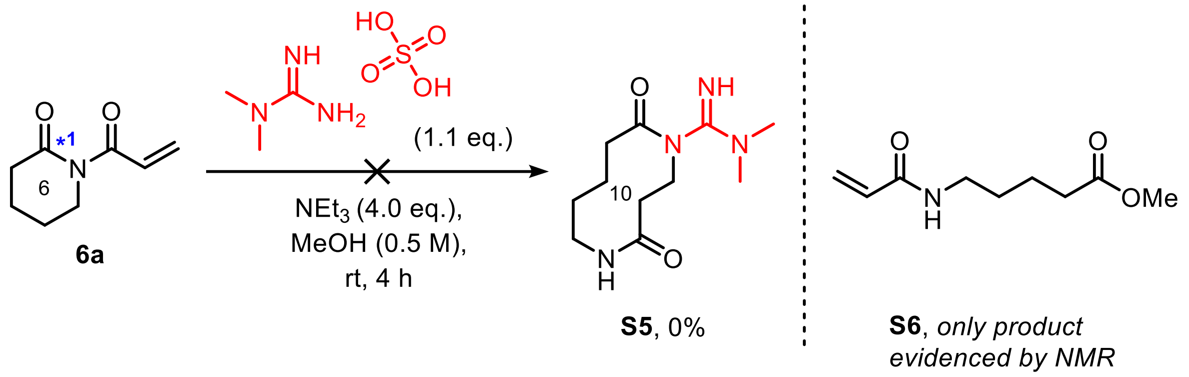

Methyl 5-acrylamidopentanoate (S6)

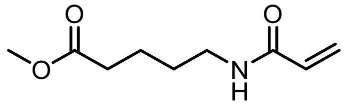

To a flask charged with dimethylguanidine sulphate (197 mg, 0.72 mmol, 1.10 eq.) in triethylamine (1.00 mL, 7.20 mmol, 10.0 eq.) was added 1-acryloyl-piperidin-2-one **2.01** (101 mg, 0.65 mmol, 1.00 eq.) as a solution in MeOH (1.50 mL). The solution was stirred at room temperature for 4 h before the solvent was removed *in vacuo* and the crude material purified by FCC (SiO_2, 1:1; ethyl acetate: hexane → 2:1; ethyl acetate: hexane → 3:1; ethyl acetate: hexane). The *title compound* was isolated as a reaction byproduct as a yellow oil (98.5 mg, 82%); R_f = 0.52 (9:1; ethyl acetate: methanol); δ_H (400 MHz, $CDCl_3$) 6.26 (dd, *J* = 17.0, 1.5 Hz, 1H, NCOC**H**CHH'), 6.08 (dd, *J* = 17.0, 10.2 Hz, 1H, NCOCHC**H**H'), 5.88 (br s, 1H, N**H**), 5.62 (dd, *J* = 10.2, 1.5 Hz, 1H, , NCOCHCH**H'**), 3.66 (s, 3H, C**H**$_3$), 3.33 (m, 2H, NHC**H**$_2$), 2.34 (t, *J* = 7.1 Hz, 2H, C**H**$_2$$CO_2$Me), 1.74 – 1.51 (m, 4H, 2 x C**H**$_2$); δ_C (101 MHz, $CDCl_3$) 174.1 (**C**O), 165.7 (**C**O), 131.0 (NCO**C**HCHH'), 126.4 (NCOCH**C**HH'), 51.7 (**C**H$_3$), 39.2 (NH**C**H$_2$), 33.6 (**C**H$_2$COMe), 29.0 (**C**H$_2$), 22.2 (**C**H$_2$); HRMS (ESI$^+$): Calcd. For $C_9H_{15}NNaO_3$, 208.0944; Found $[M+Na]^+$, 208.0945. The data obtained match those previously reported.[50]

SI4. Peptide synthesis

Solid-phase peptide synthesis (SPPS) was performed on a CEM Liberty Lite Automated Microwave Peptide Synthesiser, according to the manufacturer's standard protocols. Briefly, Fmoc-protected amino acids (5.5 equiv. (11 equiv. of Fmoc-arginine), 0.2 M in DMF) were coupled in the presence of excess *N*, *N*'-Diisopropylcarbodiimide (DIC, 125 equiv, 1.0 M in DMF) and Oxyma Pure (100 equiv, 1.0 M in DMF), as coupling agent and base respectively, under microwave irradiation at a temperature of 25 ^{o}C for 5 seconds, 78 ^{o}C for 30 seconds, 88 ^{o}C for 20 seconds, and 90 ^{o}C for 60 seconds. Fmoc deprotection was performed using 20% piperidine in DMF at 25 ^{o}C for 5 minutes. Syntheses were performed on a 0.1 mmol scale, using Rink Amide MBHA resin (*C*-terminal amide, 0.5 mmol/g loading, 1% DVB, 100-200 mesh, Fluorochem) or Wang resin pre-loaded with the *C*-terminal amino acid (*C*-terminal acid, glycine), unless otherwise stated. Where specified, *N*-terminal acetyl capping was performed in DMF (5 mL) with acetyl chloride (2 equiv.) and DIPEA (4 equiv.) at room temperature for 2 h. Prior to cleavage, the resin was washed sequentially with DCM (3 × 15 mL) and methanol (3 × 15 mL). Peptides were cleaved from the resin in 20 mL of cleavage cocktail (90% TFA, 5% H_2O, 3% TIPS, 2% DTT for Cys-containing sequences) for 4 hrs (18 hrs for Arg-containing sequences). After filtration, the resin was washed extensively with DCM (3 × 50 mL) and the filtrate concentrated *in vacuo* to ~2 mL volume. The residue was dropped into ice cold diethyl ether (~50 mL), and the resultant precipitate collected by centrifugation (3000 rpm, 5 min), resuspended in diethyl ether (~50 mL), and centrifuged again. The residual solid was allowed to air dry for 10 min, then dissolved in deionized water (10 mL) and dried by lyophilisation. Peptides were typically pure by LC-MS analysis, and used directly. In cases where the LC-MS analysis showed the sample to contain impurities, the peptide was purified by automated reverse-phase column chromatography using a Teledyne ISCO NextGen 300+ automated flash column chromatography unit on a RediSep Gold® C18 reversed phase column (SiO_2: 400–632 mesh).

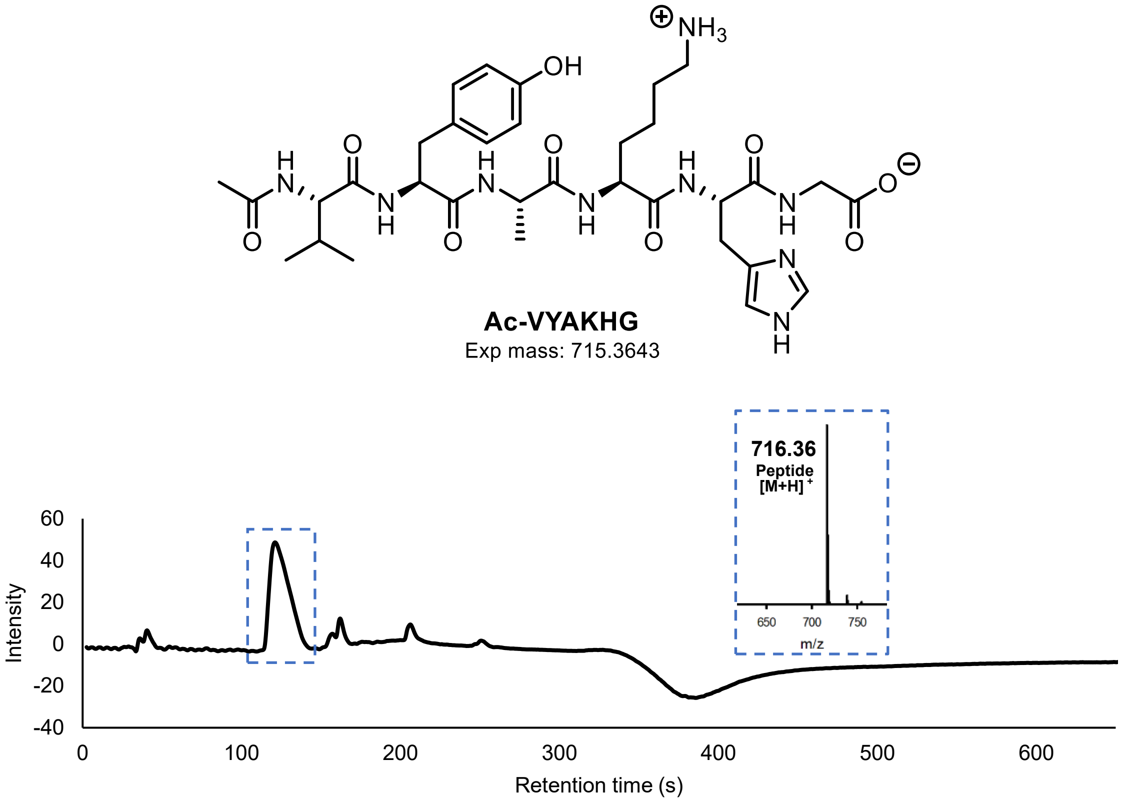

Figure S1: 280 nm chromatogram of hexapeptide (Ac-VYAKHG) made using automated solid phase peptide synthesis.

Analysis Information

Analysis Filename	wpu118460oh_P1-E-3_01_56782.d	Acquisition Date	27/03/2025 11:45:50
Method	ESI_low mass_BadTransAlt.m	Instrument	compact
Submission Name	wpu118460oh	ESI	Positive

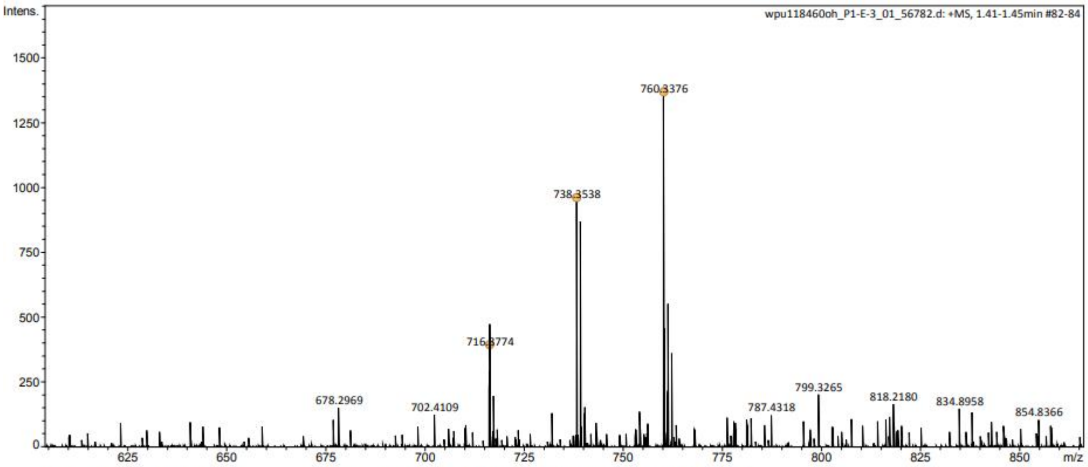

Meas. m/z	#	Ion Formula	m/z	err [ppm]	err [mDa]	mSigma	Mean err [ppm]
716.3774	1	C33H50N9O9	716.3726	-6.6	-4.8	n.a.	-2.9
738.3538	1	C33H49N9NaO9	738.3545	1.0	0.7	265.7	2.3
760.3376	1	C33H48N9Na2O9	760.3365	-1.4	-1.1	88.0	-2.1

Figure S2: HRMS spectrum of hexapeptide Ac-VYAKHG.

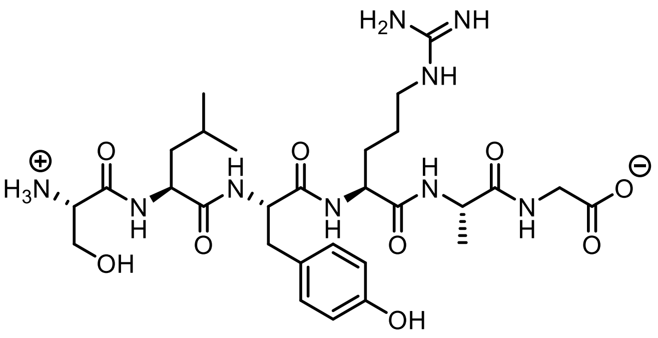

SLYRAG
Exp mass: 665.3497

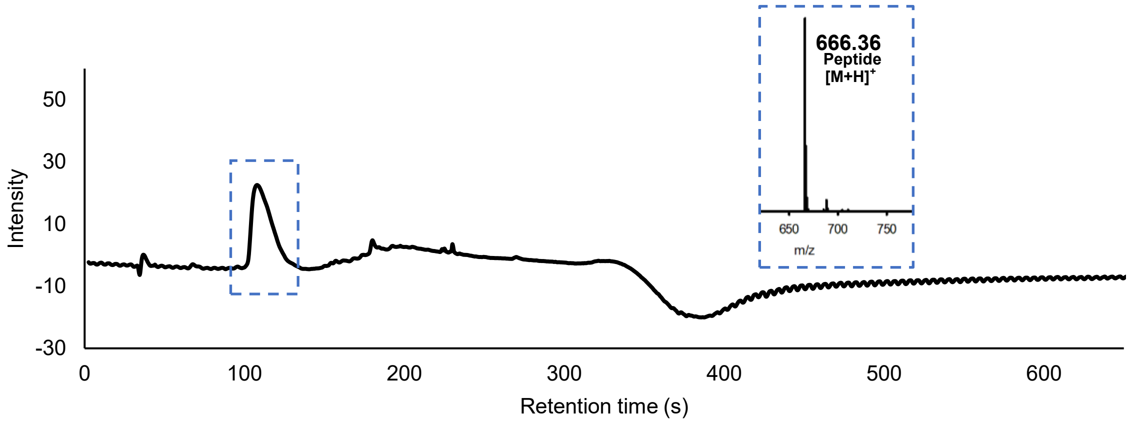

Figure S3: 280 nm chromatogram of hexapeptide (SLYRAG) made using automated solid phase peptide synthesis.

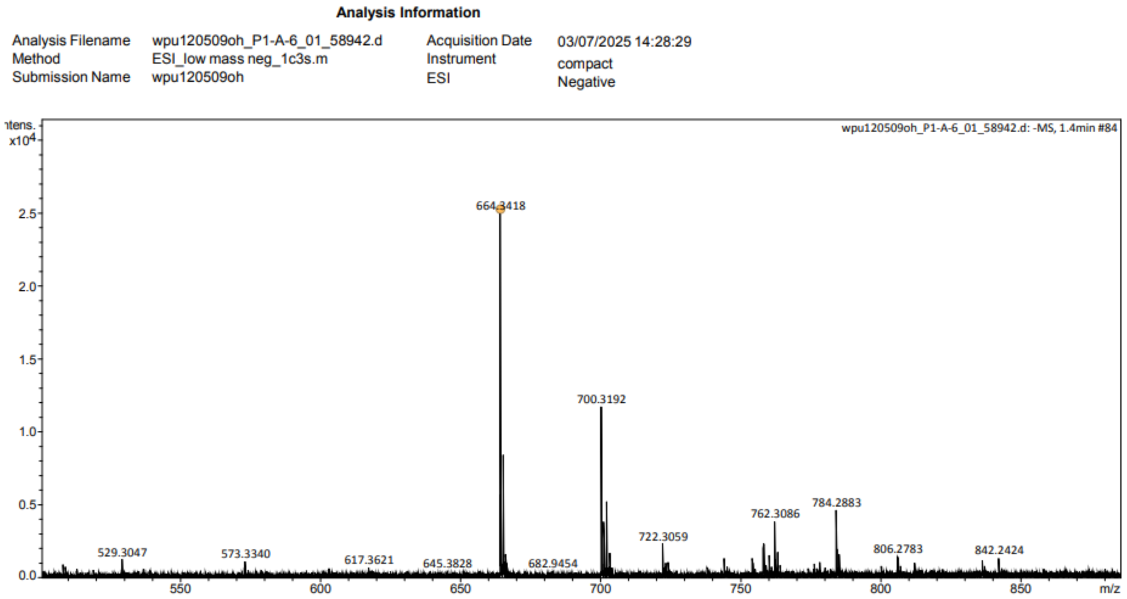

Meas. m/z	#	Ion Formula	m/z	err [ppm]	err [mDa]	mSigma	Mean err [ppm]
664.3418	1	C29H46N9O9	664.3424	0.9	0.6	11.2	0.1

Figure S4: HRMS spectrum of hexapeptide SLYRAG.

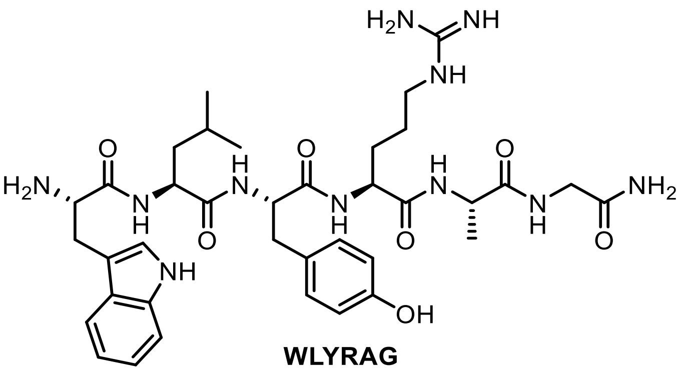

WLYRAG

Exp mass: 763.4118

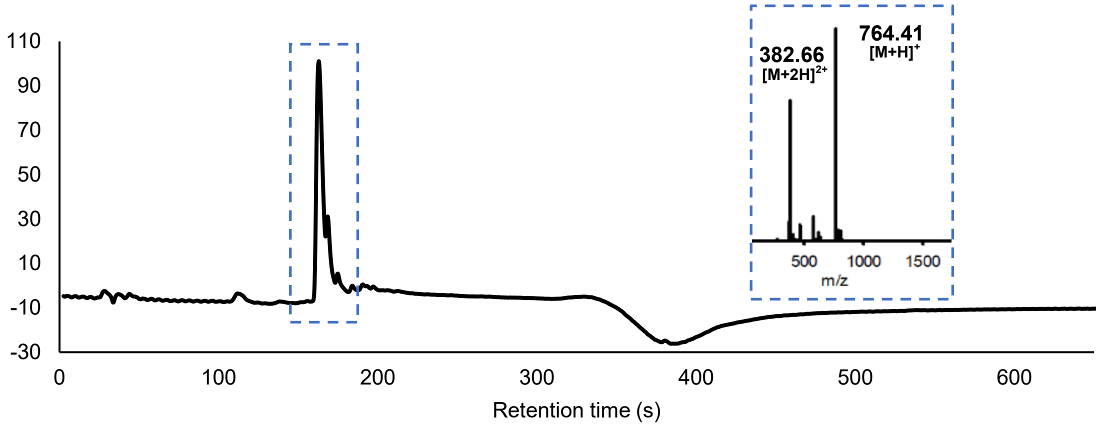

Figure S5: 280 nm chromatogram of hexapeptide (WLYRAG) made using automated solid phase peptide synthesis.

Analysis Filename	wpu118462oh_P1-E-5_01_56792.d	Acquisition Date	27/03/2025 12:23:01
Method	ESI_low mass_BadTransAlt.m	Instrument	compact
Submission Name	wpu118462oh	ESI	Positive

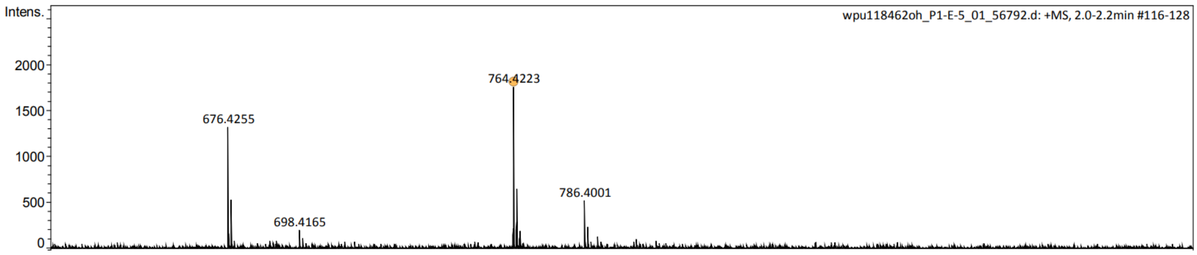

Meas. m/z	#	Ion Formula	m/z	err [ppm]	err [mDa]	mSigma	Mean err [ppm]
764.4223	1	C37H54N11O7	764.4202	-2.8	-2.1	48.3	-0.2

Figure S6: HRMS spectrum of hexapeptide WLYRAG.

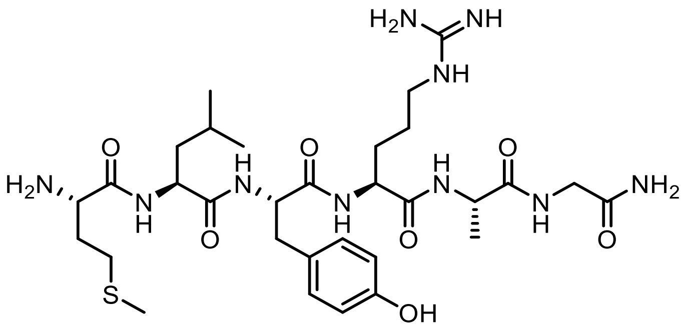

MLYRAG
Exp mass: 708.3741

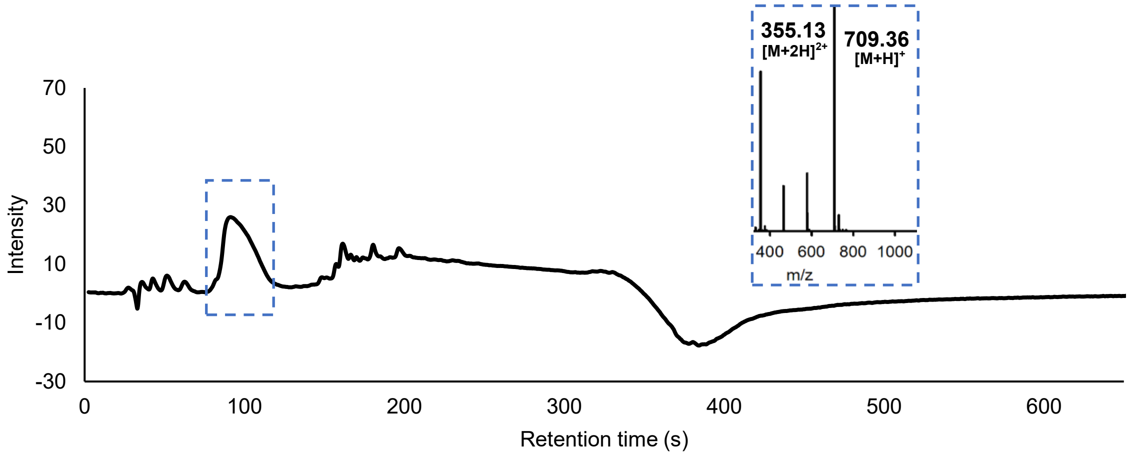

Figure S7: 280 nm chromatogram of hexapeptide (MLYRAG) made using automated solid phase peptide synthesis.

Analysis Filename	wpu118463oh_P1-E-6_01_56793.d	Acquisition Date	27/03/2025 12:27:03
Method	ESI_low mass_BadTransAlt.m	Instrument	compact
Submission Name	wpu118463oh	ESI	Positive

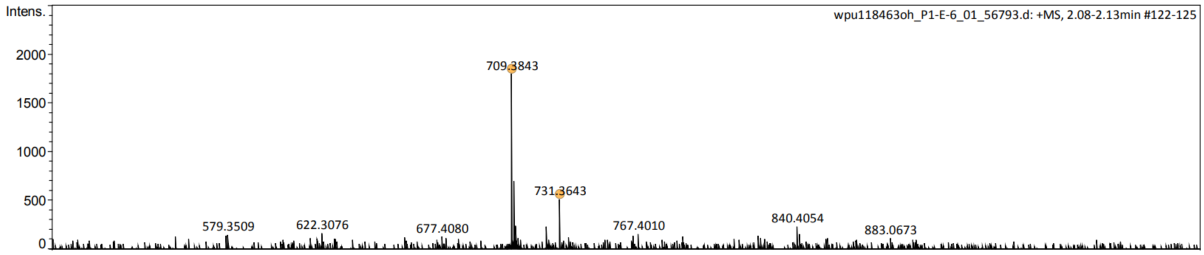

Meas. m/z	#	Ion Formula	m/z	err [ppm]	err [mDa]	mSigma	Mean err [ppm]
709.3843	1	C31H53N10O7S	709.3814	-4.2	-3.0	17.1	-3.2
731.3643	1	C31H52N10NaO7S	731.3633	-1.3	-1.0	n.a.	-0.6

Figure S8: HRMS spectrum of hexapeptide MLYRAG.

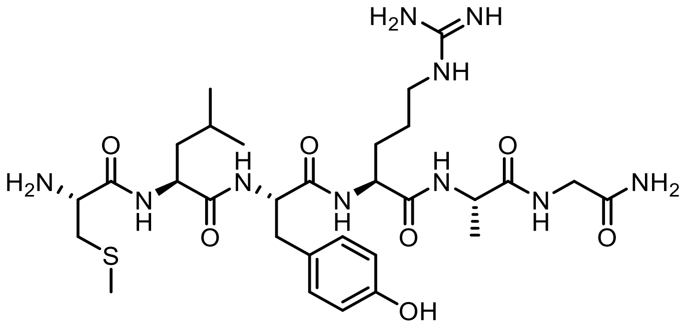

C(Me)LYRAG
Exp mass: 694.3585

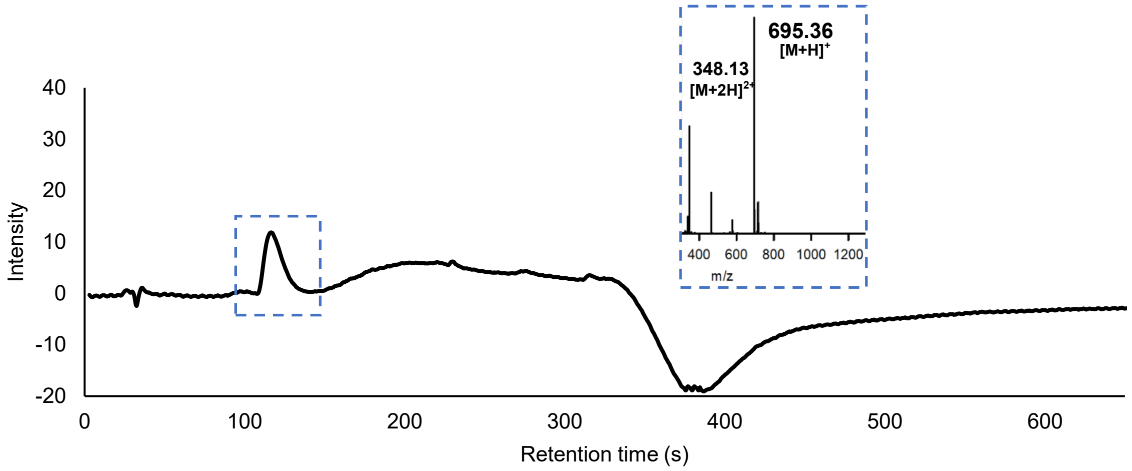

Figure S9: 280 nm chromatogram of hexapeptide (C(Me)LYRAG) made using automated solid phase peptide synthesis.

Analysis Filename	wpu118464oh_P1-E-7_01_56786.d	Acquisition Date	27/03/2025 11:57:43
Method	ESI_low mass_BadTransAlt.m	Instrument	compact
Submission Name	wpu118464oh	ESI	Positive

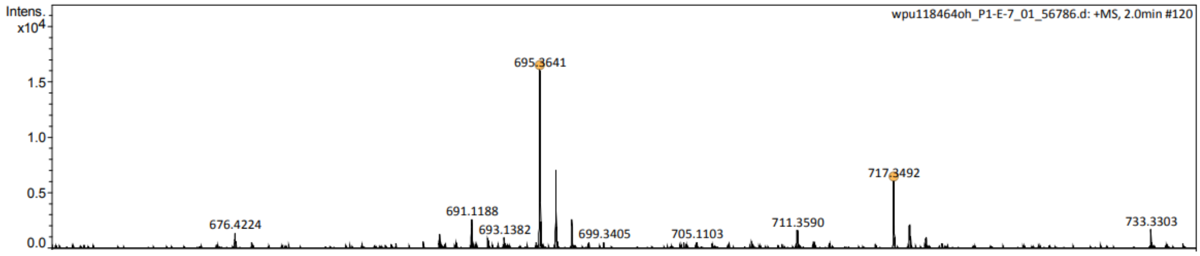

Meas. m/z	#	Ion Formula	m/z	err [ppm]	err [mDa]	mSigma	Mean err [ppm]
695.3641	1	C30H51N10O7S	695.3657	2.4	1.7	39.8	3.6
717.3492	1	C30H50N10NaO7S	717.3477	-2.2	-1.6	27.7	-4.4

Figure S10: HRMS spectrum of hexapeptide C(Me)LYRAG.

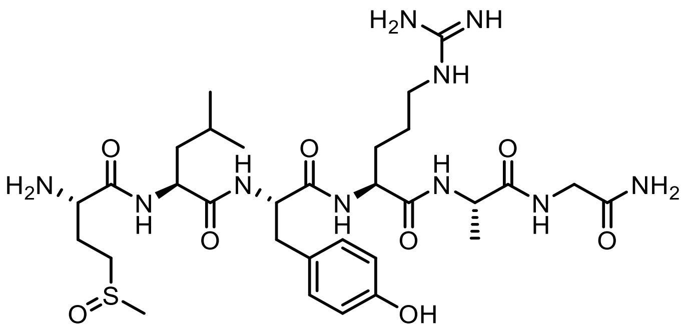

M(O)LYRAG
Exp mass: 724.3690

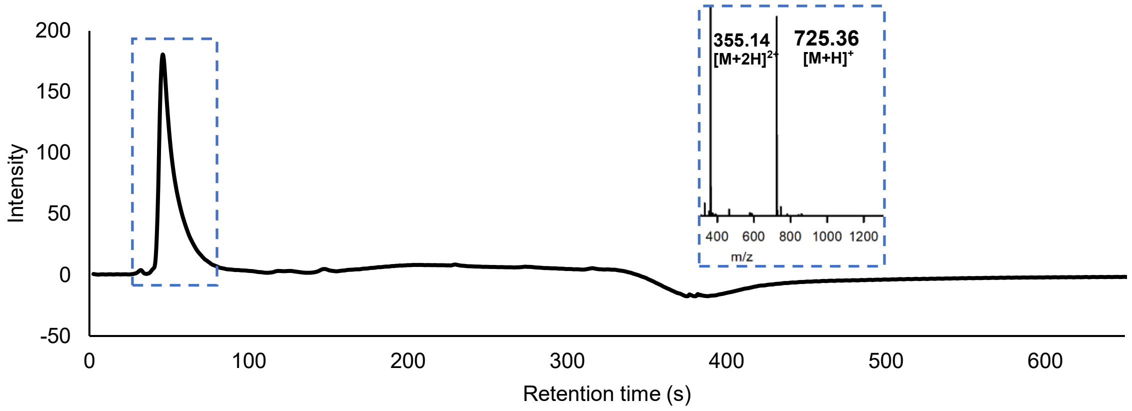

Figure S11: 280 nm chromatogram of hexapeptide (M(O)LYRAG) made using automated solid phase peptide synthesis.

Analysis Filename	wpu118583oh_P1-D-9_01_56916.d	Acquisition Date	01/04/2025 14:21:13
Method	ESI_low mass_2c1s.m	Instrument	compact
Submission Name	wpu118583oh	ESI	Positive

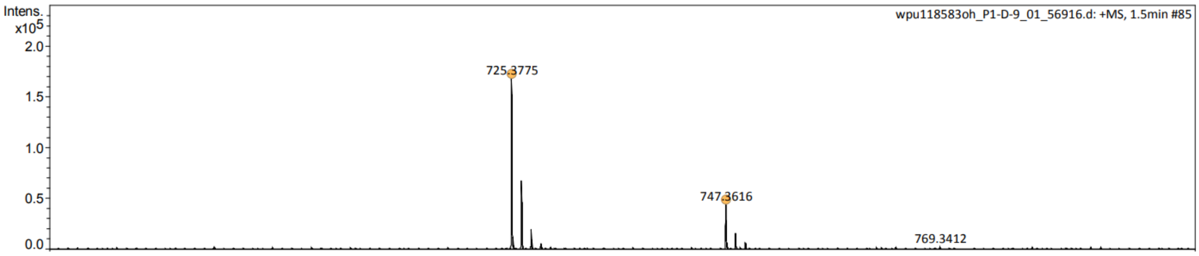

Meas. m/z	#	Ion Formula	m/z	err [ppm]	err [mDa]	mSigma	Mean err [ppm]
725.3775	1	C31H53N10O8S	725.3763	-1.6	-1.2	11.1	-1.3
747.3616	1	C31H52N10NaO8S	747.3583	-4.4	-3.3	22.0	-3.0

Figure S12: HRMS spectrum of hexapeptide M(O)LYRAG.

SI5. Peptide modifications: general procedures and materials

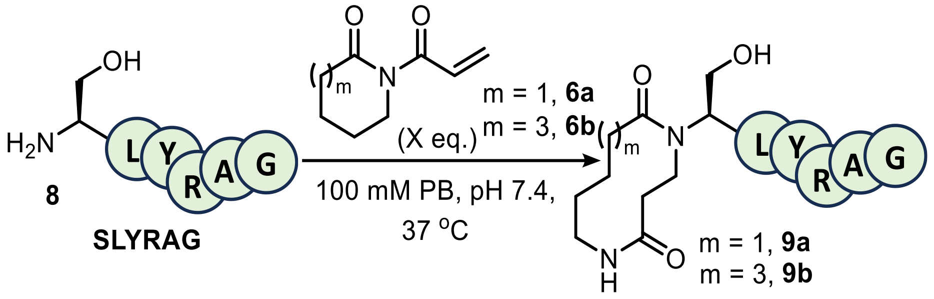

Reagents **6a** and **6b** were used to modify 6-mer SLYRAG **8** (Scheme 3a) using the conditions outlined in **General Procedure A** below:

General Procedure A: To a solution of SLYRAG **8** (10 µL, 5 mM stock in HPLC-grade water, 1 mM, 1.00 eq.), sodium phosphate buffer (10 µL, 0.5 M stock, 0.1 M, pH 7.4) and HPLC-grade water (25 µL) was added reagent (**6a** or **6b**) (5 µL; 20 mM, 50 mM, 100 mM or 500 mM stock in DMSO; 2, 5, 10 or 50 mM; 2, 5, 10 or 50 eq.). The reaction was incubated at 37 °C with agitation (1000 rpm) for up to 4 h before an aliquot was taken and analysed by LC-MS in accordance with the procedure outlined in **LC-MS method A**.

Each of the reagents **6a**, **6b**, *N*-methylmaleimide and *N*,*N*-dimethylacrylamide were used to modify 6-mer SLYRAG **8** and their conjugate's stability investigated using the conditions outlined in **General Procedure B** below:

General Procedure B: To a solution of SLYRAG **8** (20 µL, 5 mM stock in HPLC-grade water, 1 mM, 1 eq.), sodium phosphate buffer (20 µL, 0.5 M stock, 0.1 M, pH 7.4) and HPLC-grade water (50 µL) was added reagent (**6a**, **6b**, *N*-methylmaleimide or *N*,*N*-dimethylacrylamide) (10 µL, 100 mM stock in DMSO, 10 mM, 10 eq.). The reaction was incubated at 37 °C with agitation (1000 rpm) for 4 h before the crude reaction mixtures were purified on a reversed phase C18 SPE cartridge eluting with 30 % acetonitrile in HPLC-grade water and fractions containing peptide flash frozen and lyophilised. The stability of the conjugates was investigated by resuspending the samples to a concentration of 1 mM in sodium phosphate buffer (0.1 M, pH 7.4), followed by the addition of a cysteine solution (100 mM stock in HPLC-grade water, 100 uL, 10 mM, 10 eq.). The reactions were then incubated at rt with agitation (1000 rpm) whilst aliquots were taken every 15 mins and analysed by LC-MS in accordance with the procedure outlined in **LC-MS method A**.

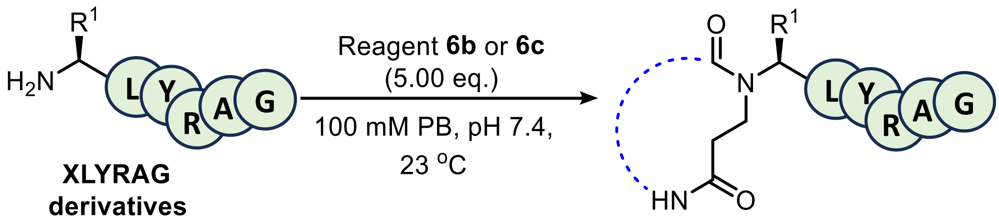

The reaction kinetics between reagents **6b** or **6c** and 6-mer XLYRAG peptides (See Figure S17) was investigated using the method described in **General Procedure C** below:

General Procedure C: To a solution of peptide (20 µL, 5 mM stock in HPLC-grade water, 1 mM, 1 eq.), sodium phosphate buffer (20 µL, 0.5 M stock, 0.1 M, pH 7.4) and HPLC-grade water (50 µL) in an LC-MS vial was added reagent (**6b** or **6c**) (10 µL, 50 mM in DMSO, 5 mM, 5 eq.). The reaction was incubated at 23 °C with no agitation. Samples were analysed every 15 minutes by LC-MS with the procedure outlined in **LC-MS method B.**

LC-MS method A: LC-MS samples were prepared by aliquoting 5 µL of crude reaction mixture into 45 µL of 1:1 acetonitrile/water with 1 % (v/v) formic acid. The samples were eluted on an Accucore™ C18 2.6 µm column (50 × 2.1 mm) (Thermoscientific) with a linear gradient 5-90 % (increasing solvent B, details found below) over 13 mins with a mobile phase flow rate of 0.3 mL/min at 30 °C.

Solvent A: HPLC-grade water with 0.1 % (v/v) formic acid.

Solvent B: HPLC-grade acetonitrile with 0.1 % (v/v) formic acid.

Conversion from starting material to modified product (bioconjugation yield, %) was calculated using the peak area of the UV 280 nm chromatograph signals corresponding to each species. Since our starting material, intermediate and product all contain a tyrosine residue, their absorbance coefficient at 280 nm was equivalent., Relative absorbance at 280 nm was therefore used to calculate conversion using **Equation 1**:

$$\frac{Area\ of\ peak\ (product)}{Area\ of\ peak\ (SM) + Area\ of\ peak\ (product)} \times 100 = Conversion\ (\%) \qquad \textbf{\textit{Equation 1}}$$

LC-MS method B: Kinetic experiments were conducted on a 100 µL scale with the samples incubated within the LC-MS instrumentation. Aliquots of the reaction mixture were injected every 15 mins and run as described in **LC-MS method A**. Each sample underwent a total of 12 injections over 3 h, and the reaction progress was monitored by calculating concentrations of each species using **Equation 2**, where AOP represents the peak area:

$$\frac{AOP\ (product)}{AOP\ (SM) + AOP\ (intermediate) + AOP\ (product)} \times 0.001 = Concentration\ (M) \qquad \textbf{\textit{Equation 2}}$$

The data from **Equation 2** was fitted in Copasi 4.34.251 to a model based on the following two-step reaction:

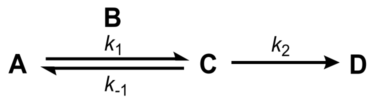

The aza-michael addition (A+B → C) was defined as a reversible reaction (forward rate constant k_1, reverse rate constant k_{-1}), whilst the ring-expansion (C → D) was defined as an irreversible transformation (forward rate constant k_2). Rate constants were estimated using the evolutionary programming method built into the software, with 200 generations and a population size of 20. Parameters were restricted within the confines of: k_1 $10^{-6} – 10^{7}$ $M^{-1}s^{-1}$; k_{-1} $10^{-8} – 10^{3}$ s^{-1}; k_2 $10^{-8} – 10^{3}$ s^{-1}.

SI6. Peptide modifications: supplementary figures

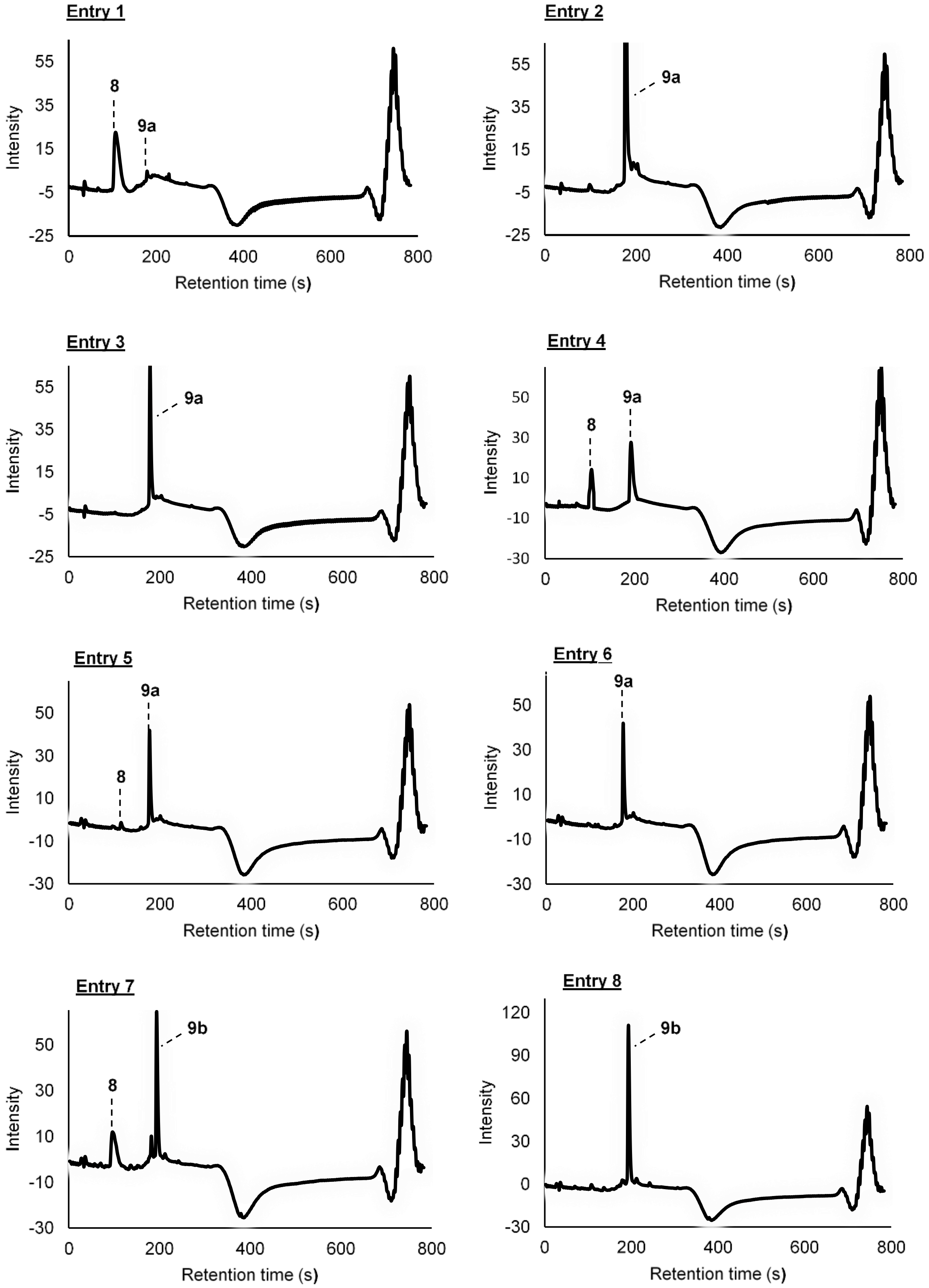

Figure S13: 280 nm UV chromatograms from LC-MS analyses of the crude reaction mixtures of SLYRAG **8** with acryloyl imides **6a** and **6b** under varying conditions (Figure 1D of the manuscript).

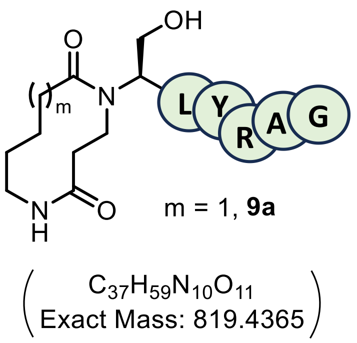

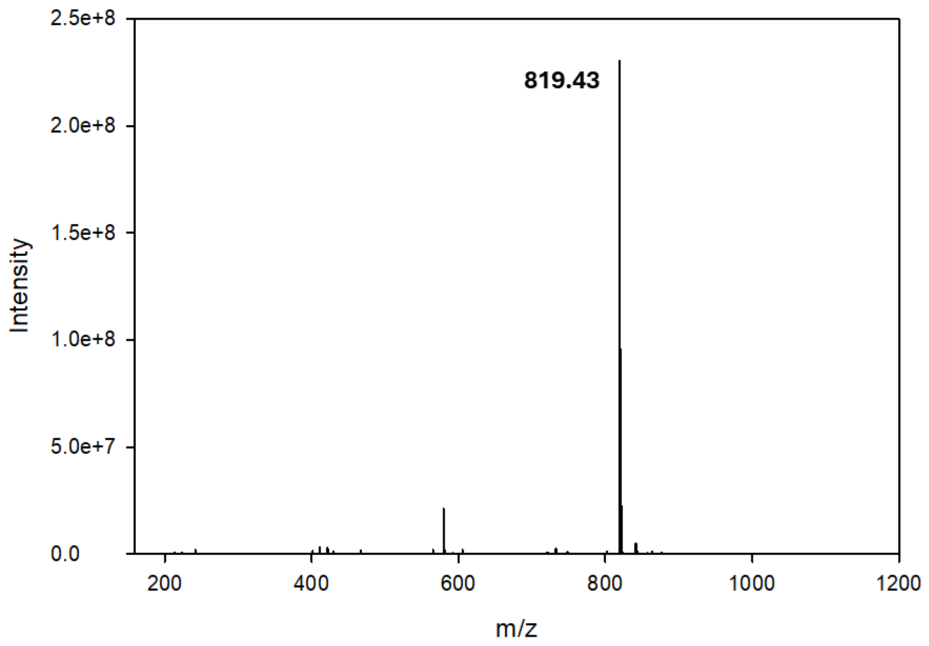

Figure S14: Mass spectrum obtained for **9a**.

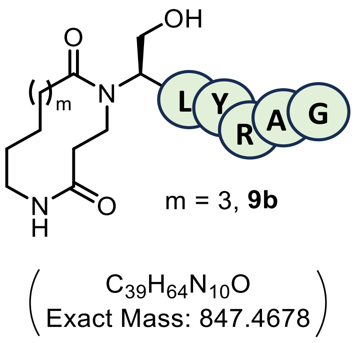

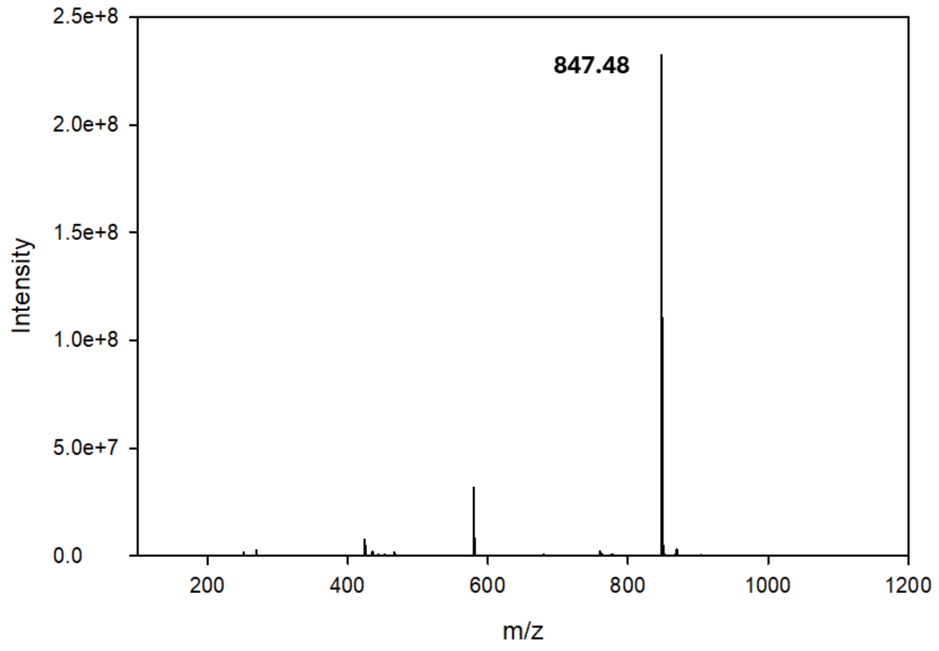

Figure S15: Mass spectrum obtained for **9b**.

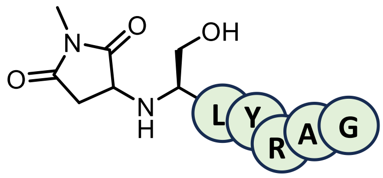

9c

($C_{34}H_{53}N_{10}O_{11}$
Exact Mass: 777.3895)

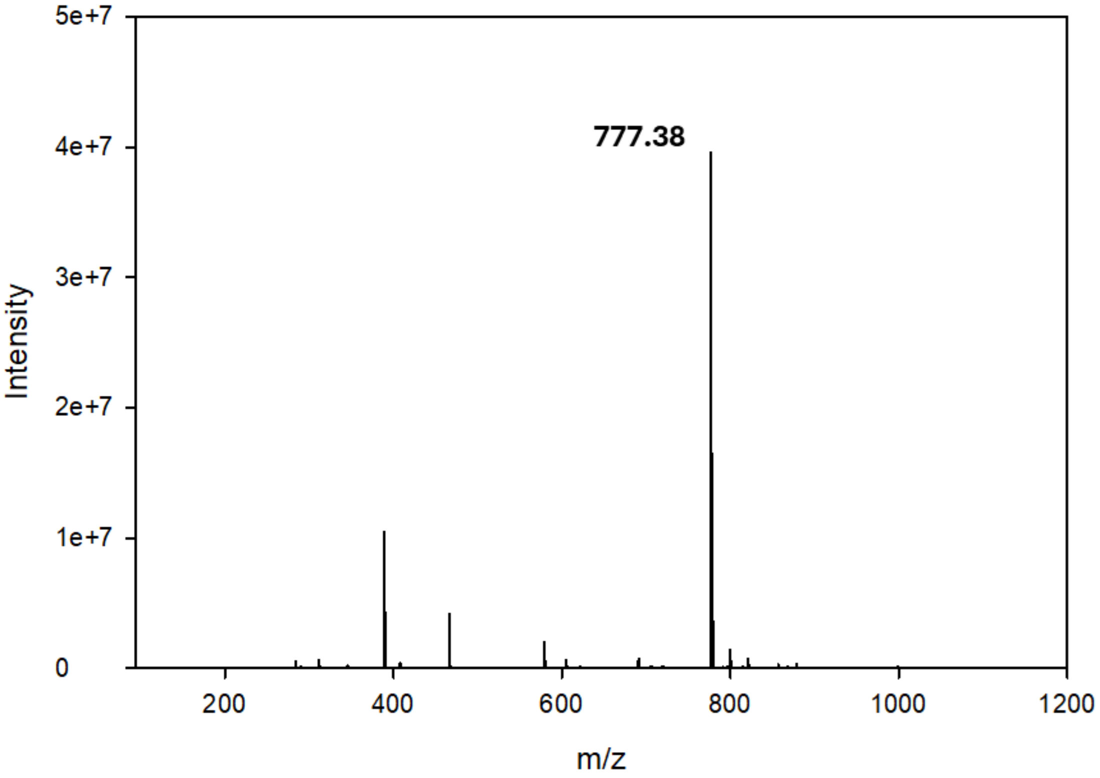

Figure S16: Mass spectrum obtained for **9c**.

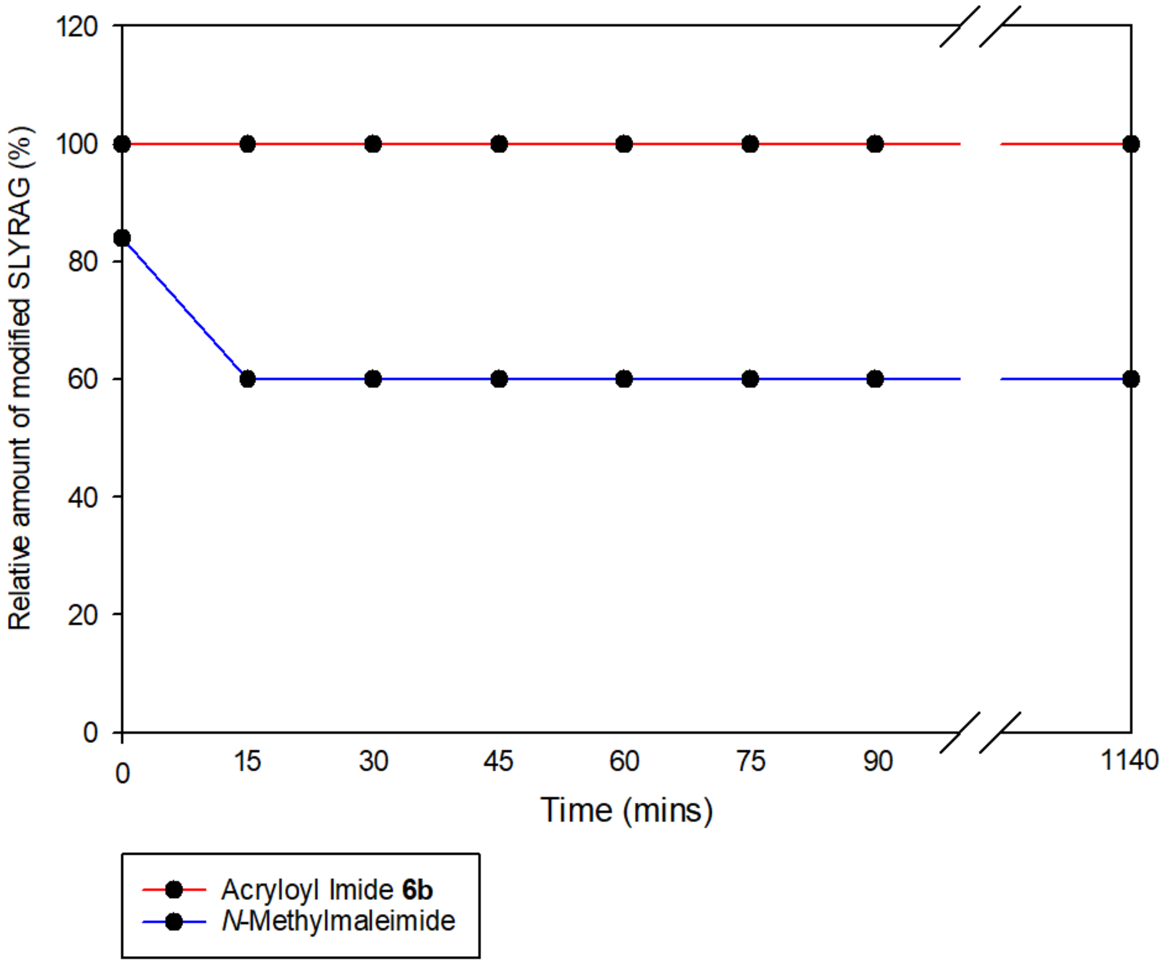

Figure S17: Stability of SLYRAG **8** modified with **6b** and *N*-methylmaleimide when incubated with 10 equivalents of cysteine at $t = 0$ mins.

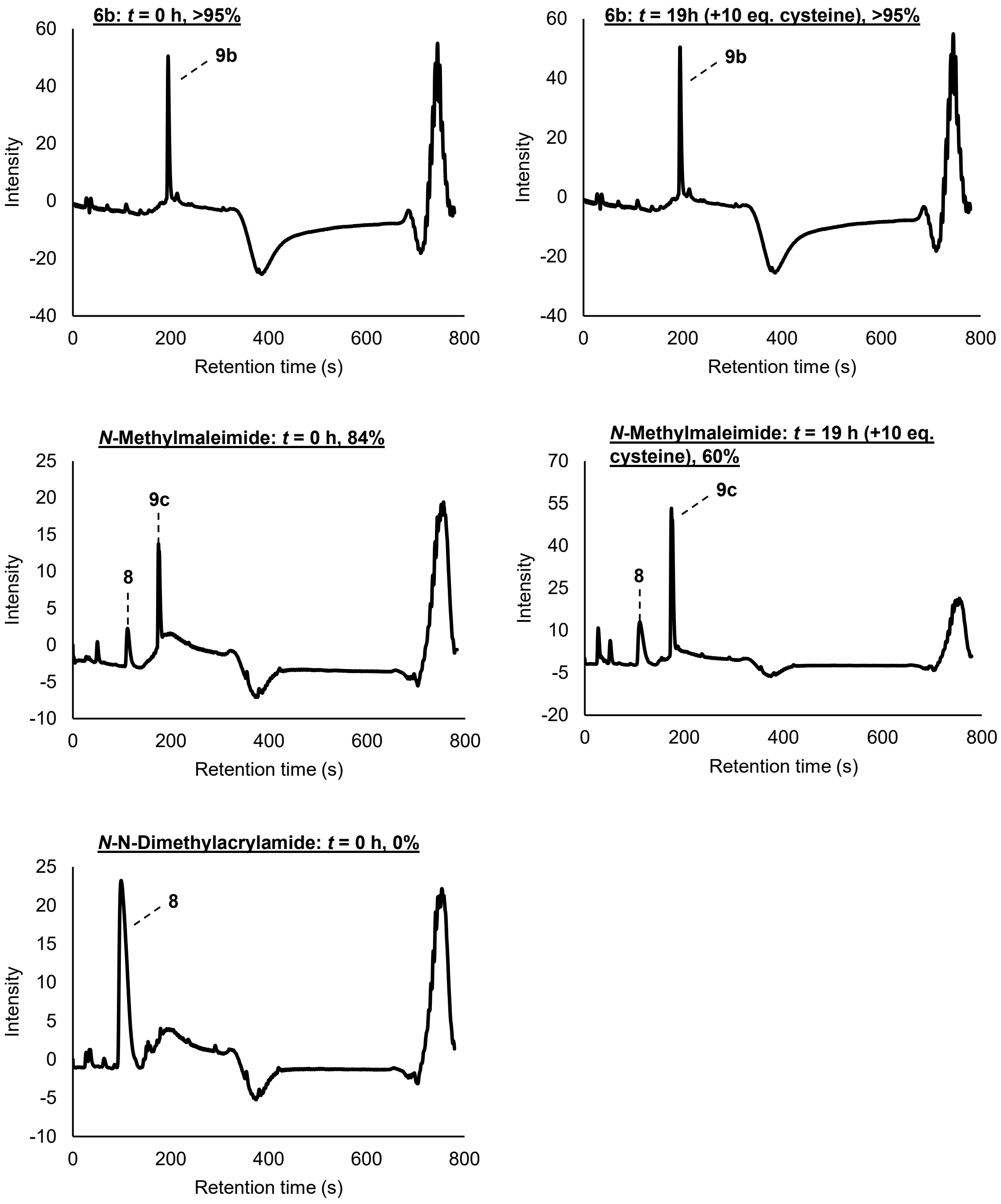

Figure S18: 280 nm UV chromatograms from LC-MS analyses of the purified conjugates of SLYRAG **8** with **6b**, *N*-methylmaleimide and *N,N*-dimethylacrylamide before and after incubation with 10 eq. of cysteine.

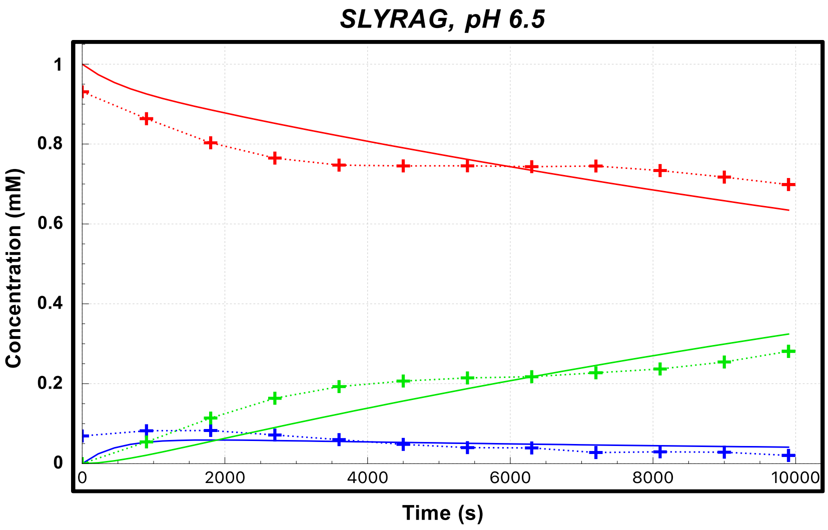

Entry	pH	Conversion after 3 h (%)	k_1 ($M^{-1}s^{-1}$)	k_{-1} (s^{-1})	k_2 (s^{-1})
1	6.5	28	0.027 ± 0.02	0.001 ± 0.001	6.60 ± 0.7 x10^{-4}

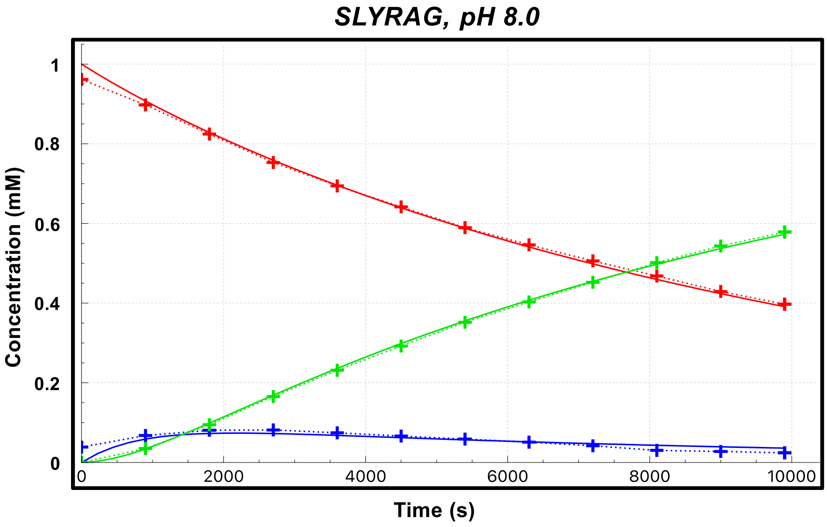

Entry	pH	Conversion after 3 h (%)	k_1 ($M^{-1}s^{-1}$)	k_{-1} (s^{-1})	k_2 (s^{-1})
3	8.0	58	0.023 ± 0.003	1.20 ± 1 x10^{-4}	0.001 ± 5 x10^{-4}

Figure S19: Plots of concentrations against time for the *N*-terminal modification of SLYRAG with **6b** at different pHs. Reactions were run as described in **General Procedure A**. Fits are based on two-step process as described above.

Ac-VYAKHG

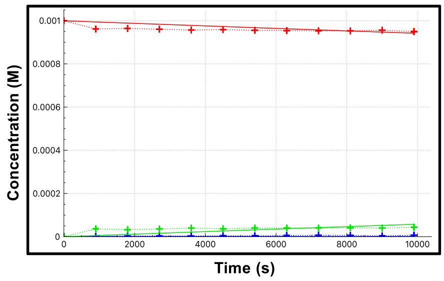

SLYRAG

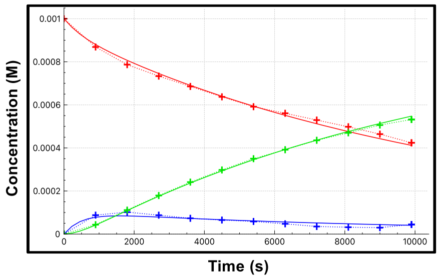

k_1	$6.3 \pm 283 \times 10^{-3}$ $M^{-1}s^{-1}$	0.040 ± 0.005 $M^{-1}s^{-1}$
k_{-1}	0.05 ± 2.75 s^{-1}	$9.7 \pm 2.6 \times 10^{-4}$ s^{-1}
k_2	0.01 ± 0.05 s^{-1}	$9.0 \pm 0.2 \times 10^{-4}$ s^{-1}

WLYRAG

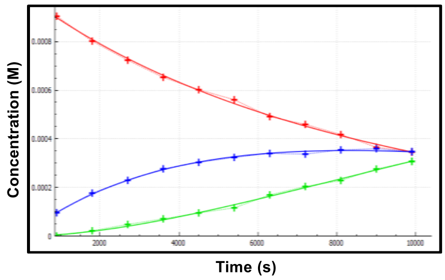

MLYRAG

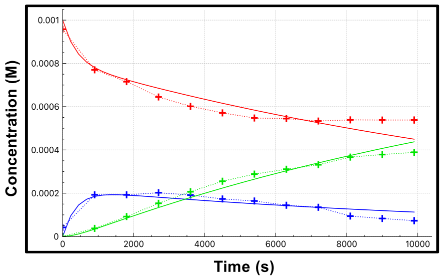

k_1	0.024 ± 0.005 $M^{-1}s^{-1}$	0.113 ± 0.03 $M^{-1}s^{-1}$
k_{-1}	$8.0 \pm 0.8 \times 10^{-6}$ s^{-1}	$1.2 \pm 0.5 \times 10^{-3}$ s^{-1}
k_2	$1.0 \pm 0.02 \times 10^{-4}$ s^{-1}	$3.0 \pm 0.1 \times 10^{-4}$ s^{-1}

C(Me)LYRAG

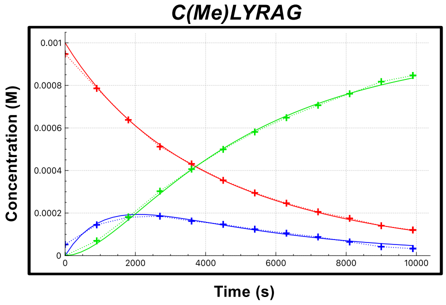

M(=O)LYRAG

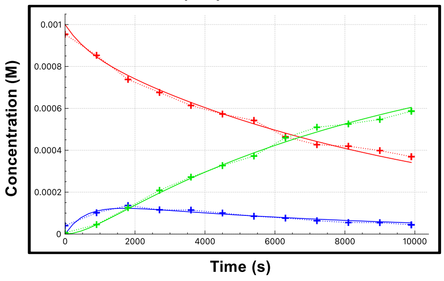

k_1	0.051 ± 0.003 $M^{-1}s^{-1}$	0.051 ± 0.6 $M^{-1}s^{-1}$
k_{-1}	$2.0 \pm 4.3 \times 10^{-5}$ s^{-1}	$8.0 \pm 1.8 \times 10^{-4}$ s^{-1}
k_2	$7.2 \pm 0.1 \times 10^{-4}$ s^{-1}	$7.0 \pm 0.2 \times 10^{-4}$ s^{-1}

Figure S20: Plots of concentrations against time for the *N*-terminal modification of XLYRAG derivatives with **6b**. Reactions were run as described in **General Procedure C**. Fits are based on two-step process as described above.

SI7. Protein expression

Expression of CjX183-D,[4] CjX183-D R51K,[4] JVZ-007,[5] and CCL5,[6] was undertaken as previously reported by our groups.

Protein sequences:

<u>CjX183-D:</u> GYLVGDATRG ANLWNTQTCV ACHGVDGERN ASGTPALTPL NPNRDLYRHS RDTQDRALRD FISMWMPQGN EGSCTGQCAA DIEAFIRTWH HHHHH

Theoretical mass (+ heme): 11226 Da

* Nb. In all experiments using CjX183-D and mutants, the observed mass was ~11 Da higher than the theoretical mass and thus was taken as the unmodified protein mass in all instances. The reasons for this observation are unknown but are consistent with previous reports.

<u>CjX183-D R51K:</u> GYLVGDATRG ANLWNTQTCV ACHGVDGERN ASGTPALTPL NPNRDLYRHS KDTQDRALRD FISMWMPQGN EGSCTGQCAA DIEAFIRTWH HHHHH

Theoretical mass (+ heme): 11198 Da

<u>JVZ-007:</u> SEVQLVESGG GLVQPGGSLT LSCAASRFMI SEYSMHWVRQ APGKGLEWVS TINPAGTTDY AESVKGRFTI SRDNAKNTLY LQMNSLKPED TAVYYCDGYG YRGQGTQVTV SS

Theoretical mass: 12121 Da

<u>CCL5:</u> SPYSSDTTPC CFAYIARPLP RAHIKEYFYT SGKCS NPAV VFVTRKNRQV CANPEKKWVR EYINSLEMS

Theoretical mass: 7847 Da

SI8. Protein modification: general procedures and materials

CARE modifications

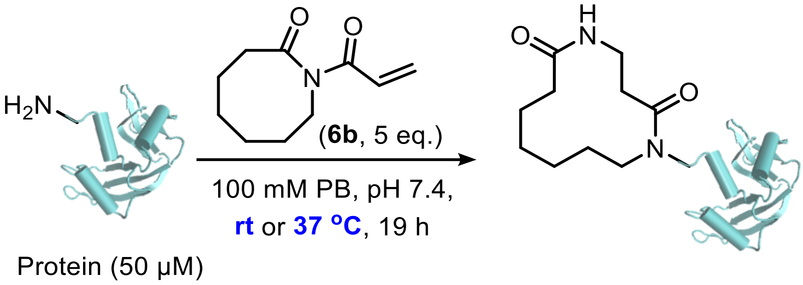

Reagent **6b** was used to modify the *N*-termini of a panel of proteins (see Figure 2C of the manuscript) using the conditions outlined in **General Procedures D1 and D2** below:

General Procedure D1: *Modification of CjX183-D, RNase A, myoglobin and cytochrome C with* ***6b****:* A stock solution of **6b** (2.00 μL, 2.5 mM in DMSO, 5 nmol, 5 eq.) was added to a mixture of a stock solution of protein (6.67 μL, 150 μM in 0.1 M, pH 7.4 sodium phosphate buffer, 1 nmol, 1 eq.), sodium phosphate buffer (4 μL, 0.5 M , pH 7.4) and HPLC-grade water (7.33 μL). The mixture was incubated at rt or 37 °C with agitation (1000 rpm) for 19 h before analysis by LC-MS in accordance with the procedure outlined in **LC-MS method C**.

General Procedure D2: *Modification of CjX183-D R51K with* ***6b****:* A stock solution of **6b** (2.00 μL, 2.5 mM in DMSO, 5 nmol, 5 eq.) was added to a mixture of a stock solution of CjX183-D R51K (9.17 μL, 109 μM in 0.1 M, pH 7.4 sodium phosphate buffer, 1 nmol, 1 eq.), sodium phosphate buffer (4 μL, 0.5 M stock, 0.1 M, pH 7.4) and HPLC-grade water (4.83 μL). The mixture was incubated at rt or 37 ºC with agitation (1000 rpm) for 19 h before analysis by LC-MS in accordance with the procedure outlined in **LC-MS method C**.

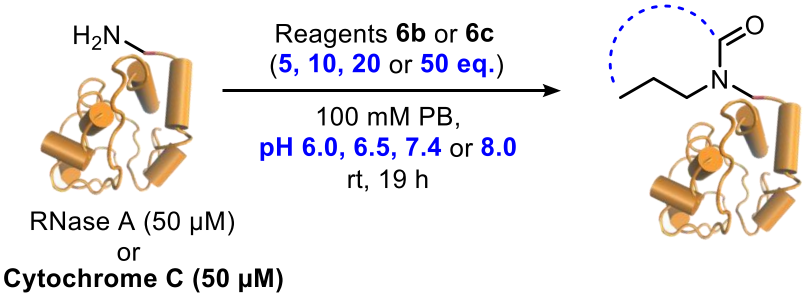

Conditions were screened for the modification of the *N*-termini of RNase A and Cytochrome C with reagents **6b** and **6c** (see Figure 2D of the manuscript and Figure S20) using the conditions outlined in **General Procedure D3** below:

General Procedure D3: *For screening conditions for the modification of RNase A and Cytochrome C with* ***6b*** *and* ***6c***: A stock solution of **6b** or **6c** (2.00 μL, 2.5, 5, 10 or 25 mM stock in DMSO, 5, 10, 20

or 50 nmol, 5, 10, 20 or 50 eq.) was added to a mixture of a stock solution of RNase A (6.67 µL, 150 µM in 0.1 M, pH 7.4 sodium phosphate buffer, 1 nmol, 1 eq.), sodium phosphate buffer (4 µL, 0.5 M stock, 0.1 M, pH 6.0, 6.5, 7.4 or 8.0) and HPLC-grade water (7.33 µL). The mixture was incubated at rt with agitation (1000 rpm) for 19 h before analysis by LC-MS in accordance with the procedure outlined in **LC-MS method C**.

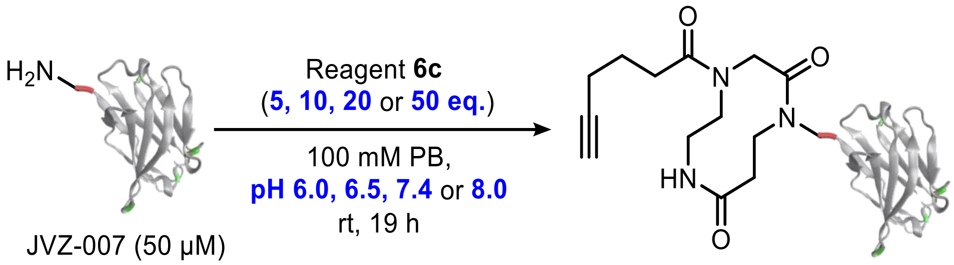

Conditions were screened for the modification of the *N*-termini of JVZ-007 with reagent **6c** (see Figure 3A of the manuscript) using the conditions outlined in **General Procedure D4** below:

General Procedure D4: *For screening conditions for the modification of JVZ-007 with* ***6c****:* A stock solution of **6c** (2.00 µL, 2.5, 5, 10 or 25 mM stock in DMSO, 5, 10, 20 or 50 nmol, 5, 10, 20 or 50 eq.) was added to a mixture of a stock solution of JVZ-007 (5.92 µL, 168.9 µM in 0.1 M, pH 7.4 sodium phosphate buffer, 1 nmol, 1 eq.), sodium phosphate buffer (4 µL, 0.5 M stock, 0.1 M, pH 6.0, 6.5, 7.4 or 8.0) and HPLC-grade water (8.08 µL). The mixture was incubated at rt with agitation (1000 rpm) for 19 h before analysis by LC-MS in accordance with the procedure outlined in **LC-MS method C**.

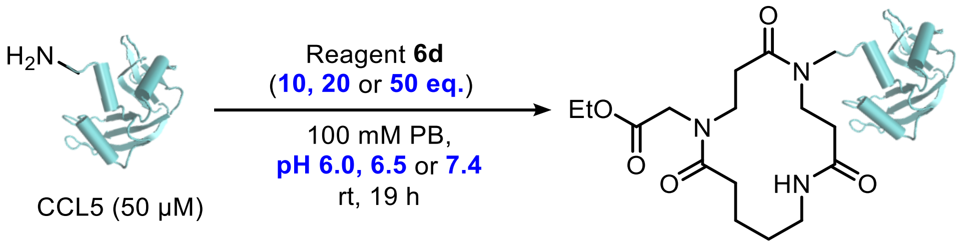

Conditions were screened for the modification of the *N*-termini of CCL5 with reagent **6e** (see Figure 5B of the manuscript) using the conditions outlined in **General Procedure D5** below:

General Procedure D5: *For screening conditions for the modification of CCL5 with* ***6e****:* A stock solution of **6e** (2.00 µL, 2.5, 5, 10 or 25 mM stock in DMSO, 5, 10, 20 or 50 nmol, 5, 10, 20 or 50 eq.) was added to a stock solution of CCL5 (18.0 µL, 55.6 µM in 0.1 M, pH 6.0, 6.5, 7.4 or 8.0 sodium phosphate buffer, 1 nmol, 1 eq.). The mixture was incubated at rt with agitation (1000 rpm) for 19 h before analysis by LC-MS in accordance with the procedure outlined in **LC-MS method C**.

LC-MS analyses of protein modifications

LC-MS method C: LC-MS samples were prepared by aliquoting 5 µL of crude reaction mixture into 45 µL of 1:1 acetonitrile/water with 1 % (v/v) formic acid. The samples were eluted on an Accucore™ 150-C4 2.6 µm column (100 × 2.1 mm) (Thermoscientific) with a linear gradient 10-90% (increasing solvent B) over 15 minutes with a mobile phase flow rate of 0.3 mL/min at 30 °C.

Solvent A: HPLC-grade water with 0.1% (v/v) formic acid.

Solvent B: HPLC-grade acetonitrile with 0.1% (v/v) formic acid.

Conversion from starting material to desired product (bioconjugation yield, %) was calculated by the MS peak intensity of each species after 'Protein Charge Deconvolution' using the ESI Compass 1.3 DataAnalysis V4.1 software (Bruker Daltonics). The deconvoluted *m/z* values were matched to their respective species and their relative intensities used to calculate conversion using Equation 3:

$$\frac{Intensity\ of\ peak\ (product)}{Int.of\ peak\ (SM) + Int.of\ peak\ (product)} \times 100 = Conversion\ (\%)$$ ***Equation 3***

Copper-catalysed azide-alkyne cycloaddition (CuAAC) of modified JVZ-007 samples

Crude reaction mixtures of JVZ-007 conjugates modified with **6c** were dialysed into sodium phosphate buffer (100 mM, pH 7.4) (SnakeSkin™ Dialysis Tubing, 3.5K MWCO). To a solution of **modified JVZ-007** (50 µL, 40 µM in 0.1 M, pH 7.4 sodium phosphate buffer, 2 nmol, 1 eq.) was added a stock solution of AZ647-N_3 (0.8 µL, 5 mM stock in DMSO, 4 nmol, 2 eq.), a stock solution of $CuSO_4$ (2 µL, 200 mM stock in HPLC-water, 400 nmol, 200 eq.) and a stock solution of (+)-sodium L-ascorbate (8.3 µL, 15 mg/mL stock in HPLC-water, 628 nmol, ~315 eq.). The reaction vessel was sealed and protected from light and agitated for 60 mins. After this time the sample was dialysed into phosphate buffered saline (100 mM, pH 7.4) (SnakeSkin™ Dialysis Tubing, 3.5K MWCO) for 19 h before being used in further analyses.

Tricine sodium dodecyl sulphate polyacrylamide gel electrophoresis (TSDS-PAGE)

TSDS-PAGE analysis was performed on a 6% (w/v) stacking polyacrylamide gel atop a 15% (w/v) resolving polyacrylamide gel using the procedures and recipes found in the literature.[3] To 15 µL of sample (50 µM) was added 5 µL of loading buffer (2% SDS, 2 mM 2-mercaptoethanol, 4% glycerol, 40 mM Tris-HCl and 0.01% bromophenol blue) before being heated at 40 °C for 1 h. 5 µL of protein ladder (PageRuler Plus Prestained Protein Ladder (Thermoscientific)) was added to the first well and 15 µL of boiled sample added to successive wells. The gel was ran at 60 mA, 100 V for 45 mins before increasing to 100 mA, 150 V for around 3.5 h, or until the gel front was approaching the bottom of the

gel. The gels were then carefully removed from the PAGE apparatus and covered in fixing solution (50% MeOH, 10% AcOH, 100 mM ammonium acetate) and rocked for 1 h. The gels were then stained in 0.1% Coomassie Brilliant Blue R-250 (50% MeOH, 10% AcOH) for 24 h before destaining in 50% MeOH, 10% AcOH. Images were captured on a Syngene G:BOX Chemi XRQ equipped with Synoptics 4.0 MP camera, with GeneSys software.

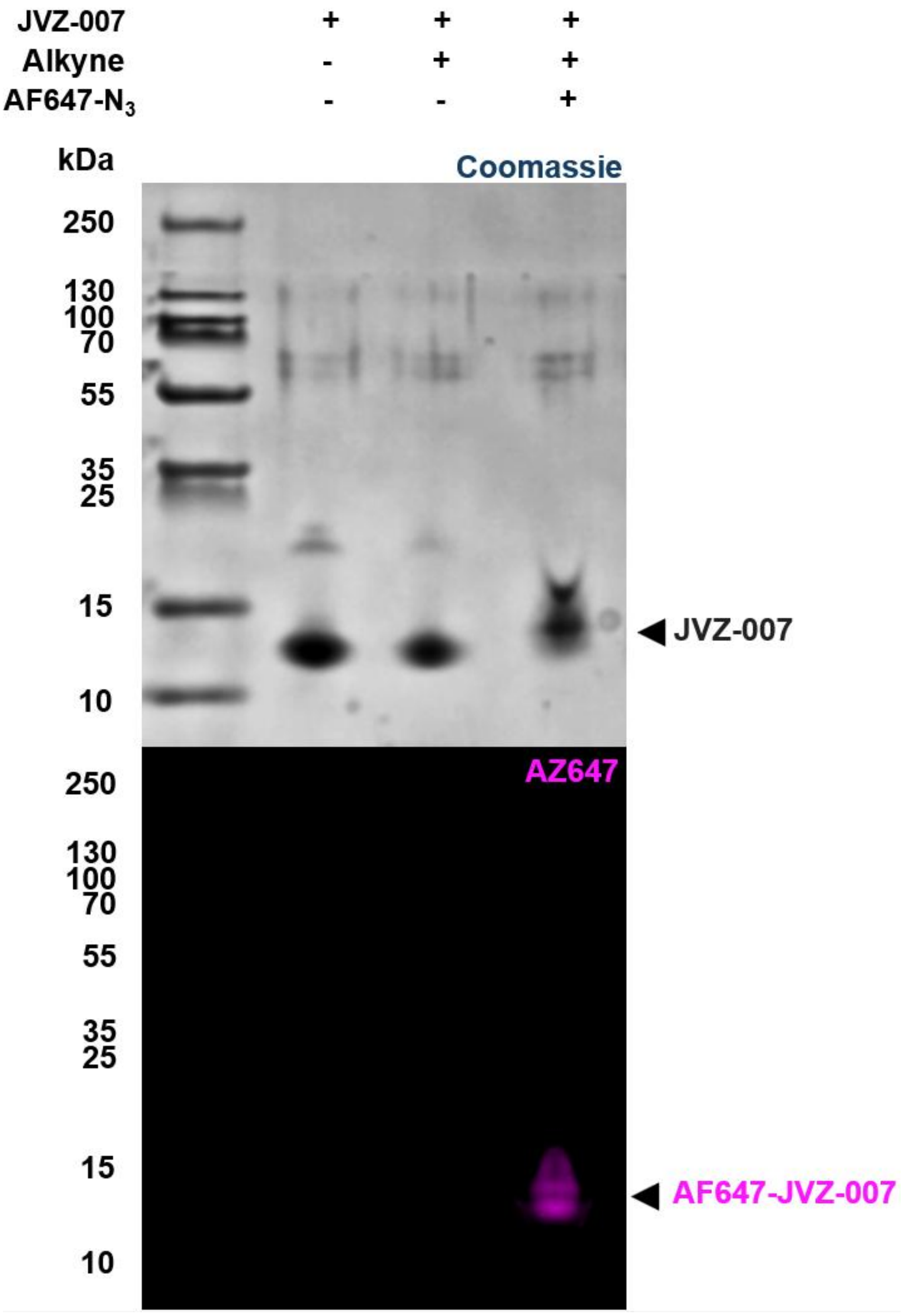

Figure S21: Analysis of **JVZ-007**, **Modified JVZ-007**, and **AF647-JVZ-007** by TSDS-PAGE and visualisation by initial fluorescent imaging (bottom), followed by Coomassie Staining (top).

Protein M_W by linear MALDI-MS

A 1 mL aliquot of 100 mM protein solution in aqueous 10 mM ammonium acetate was applied to a ground steel MALDI target plate, followed immediately by an equal volume of a freshly-prepared 10 mg/mL solution of sinapinic acid (Sigma, 85429) in 50% aqueous (v:v) acetonitrile containing 0.1% , trifluoroacetic acid (v:v).

Positive-ion MALDI mass spectra were acquired using a Bruker ultrfleXtreme in linear mode, equipped with a Nd:YAG smart beam laser. Bruker FlexControl (version 3.4) was used to operate acquisition. Laser power was manually adjusted for each sample. MS spectra were acquired over a range of 5,000-20,000 *m/z*. Spectra were smoothed using SavitzkyGolay, 5 *m/z*, 3 cycles, before centroid peak picking to average masses with a signal to nose threshold of 3. Bruker flexAnalysis software (version 3.4) was used to perform spectral processing and peak list generation.

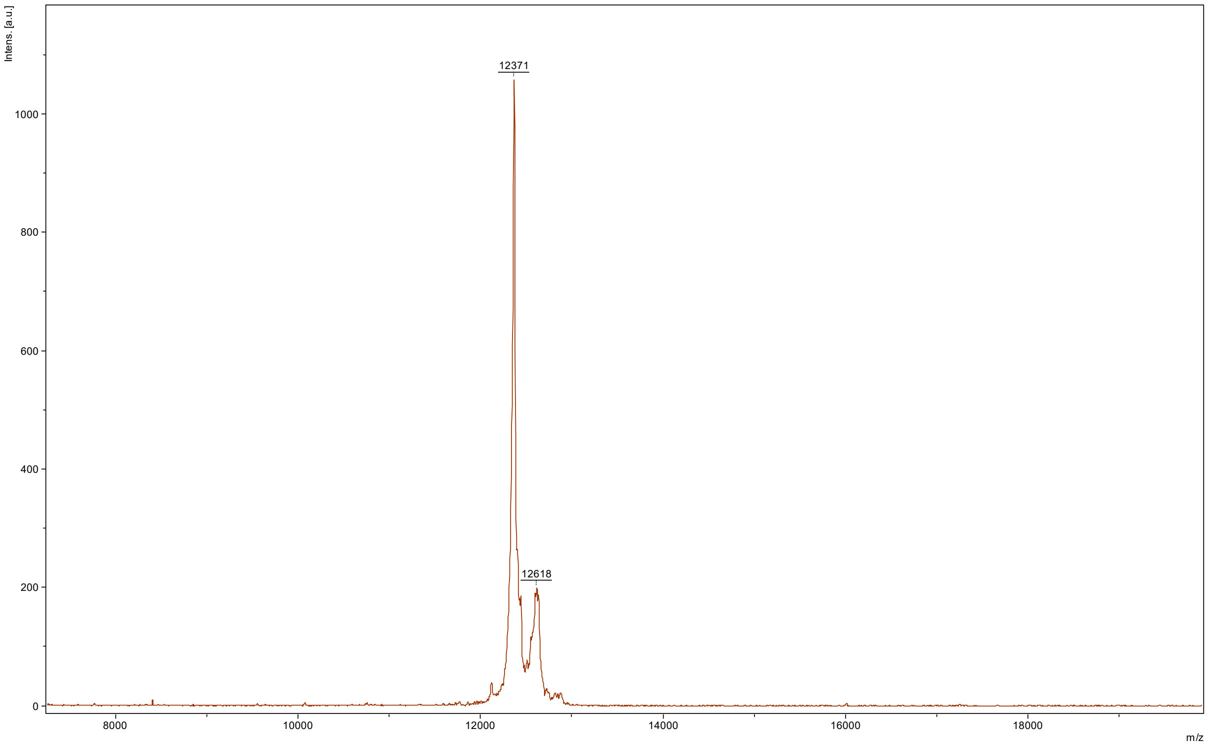

Figure S22: Intact protein M_W by MALDI of JVZ-007 conjugated with **6c** (Scheme 7a) found two *m/z* values at 12,370 Da [M (singular modification] +H]$^+$ and 12,618 Da [M (double modification]$^+$ in >10:1 ratio.

Terminal verification by MALDI-ISD

A 1 mL aliquot of 100 mM protein solution in aqueous 10 mM ammonium acetate was applied to a ground steel MALDI target plate, followed immediately by an equal volume of a freshly-prepared saturated solution of 1,5-Diaminonaphthalene matrix (Sigma, 56451) in 50% aqueous (v:v) acetonitrile containing 0.1% , trifluoroacetic acid (v:v).

Positive-ion MALDI-ISD mass spectra were acquired using a Bruker ultrfleXtreme in reflectron mode, equipped with a Nd:YAG smart beam laser. Bruker FlexControl (version 3.4) was used to operate acquisition. MS spectra were acquired over a range of 800-4,000 *m/z*. Monoisotopic peak detection used a SNAP averagine algorithm (C 4.9384, N 1.3577, O 1.4773, S 0.0417, H 7.7583) with a minimum S/N of 3. Laser power was manually adjusted for each sample. Bruker flexAnalysis software (version 3.4) was used to perform spectral processing, peak list generation and annotation. In source decay, c-ion series was manually annotated onto the obtained spectrum for N-terminal identification, allowing for a mass error of 0.5 Da.

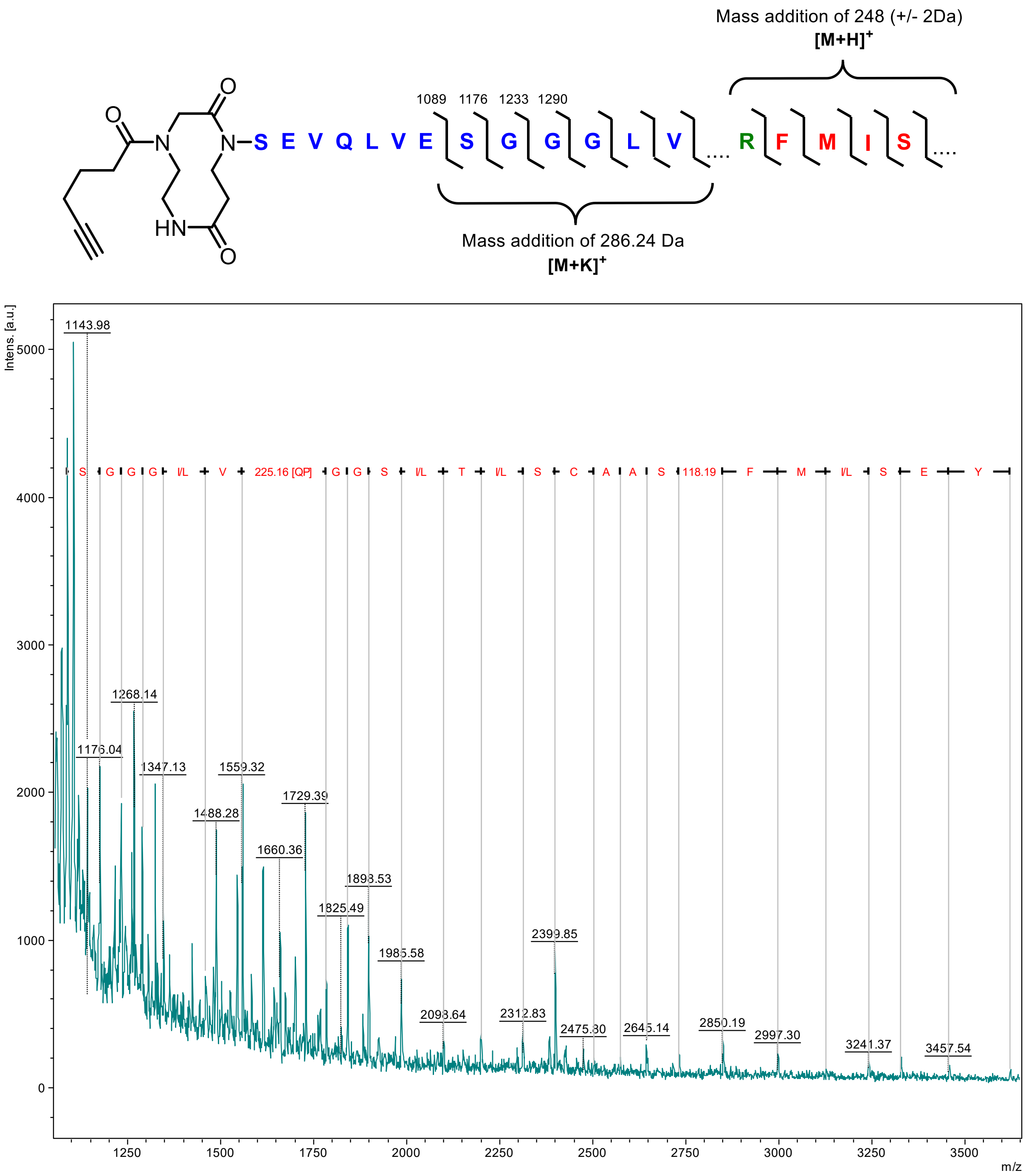

Figure S23: MALDI-ISD *N*-terminal sequencing spectrum of JVZ-007 + **6c** conjugates found the *N*-terminal SEVQLVE sequence consistent with mass addition of 284 Da $[M+K]^+$ as would be expected for our modification at the *N*-terminus.

SI9. Protein modifications: supplementary figures

CjX183-D: *was modified using General Procedure D1 at 37 ºC.*

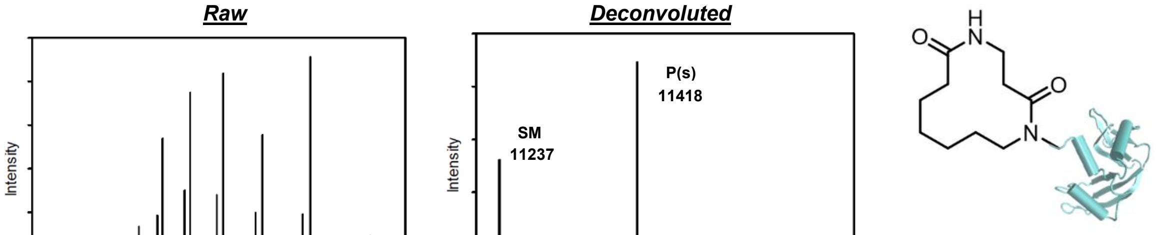

CjX183-D + 6b

63%, 100% (s)

MS (ESI+): [**SM**+H]+ found 11237, calculated 11226; [SM+H_2O+H]+ found 11253, calculated 11244; [**P(s)**+H]+ found 11418, calculated 11407; [**P(s)**+H_2O+H]+ found 11437, calculated 11425.

CjX183-R51K: *was modified using General Procedure D2 at 37 ºC and rt.*

6b at 37 ºC:

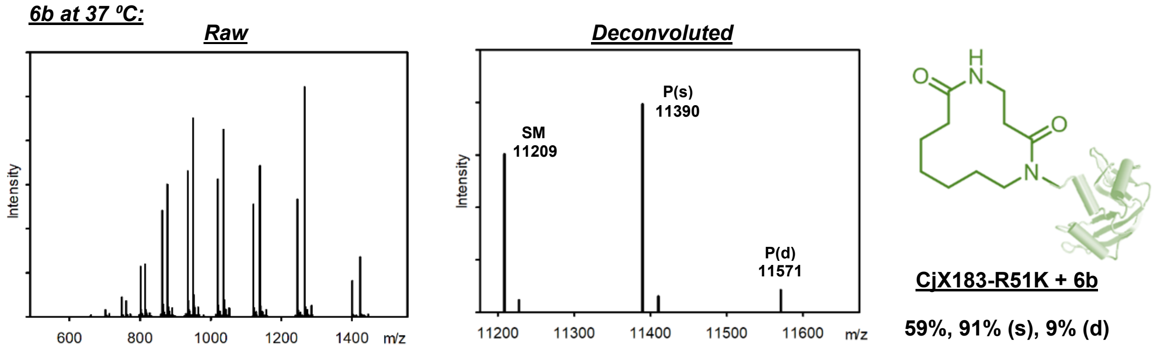

CjX183-R51K + 6b

59%, 91% (s), 9% (d)

MS (ESI+): [**SM**+H]+ found 11209, calculated 11198; [**SM**+H_2O+H]+ found 11228, calculated 11216; [**P(s)**+H]+ found 11390, calculated 11379; [**P(s)**+H_2O+H]+ found 11410, calculated 11397; [**P(d)**+H]+ found 11571, calculated 11560.

6b at rt:

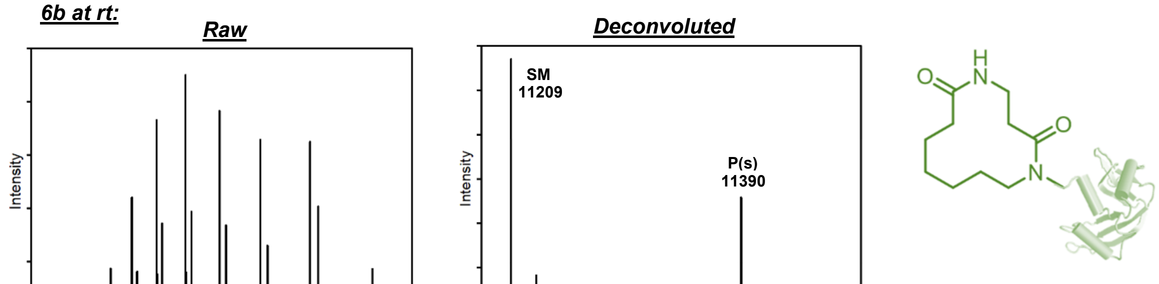

CjX183-R51K + 6b

42%, 100% (s),

MS (ESI+): [**SM**+H]+ found 11209, calculated 11198; [**SM**+H_2O+H]+ found 11228, calculated 11216; [**P(s)**+H]+ found 11390, calculated 11379.

RNase A: *was modified using General Procedure D1 at 37 ºC and rt.*

6b at 37 ºC:

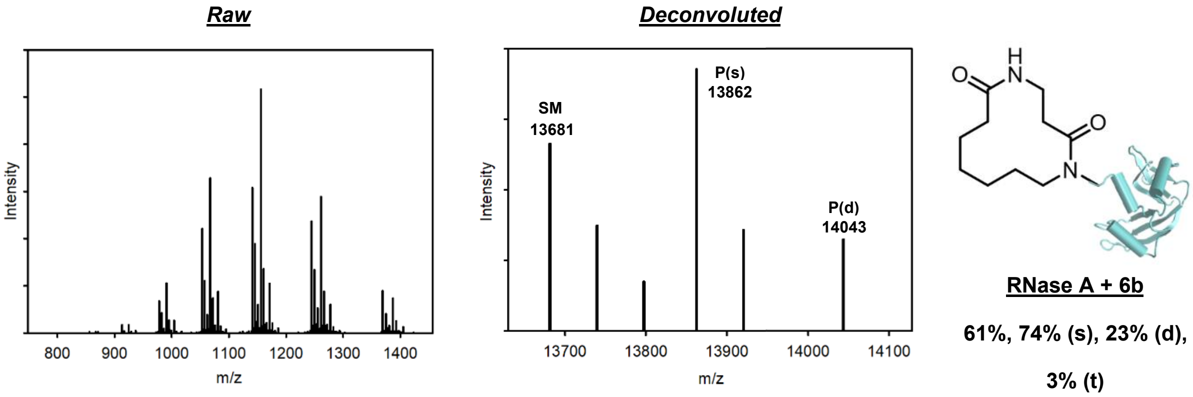

MS (ESI$^+$): [**SM**+H]$^+$ found 13681, calculated 13681; [**SM**+58]$^+$ found 13739; [**SM**+116]$^+$ found 13798; [**P(s)**+H]$^+$ found 13862, calculated 13862; [**P(s)**+58]$^+$ found 13920; [**P(d)**+H]$^+$ found 14043, calculated 14043.

6b at rt:

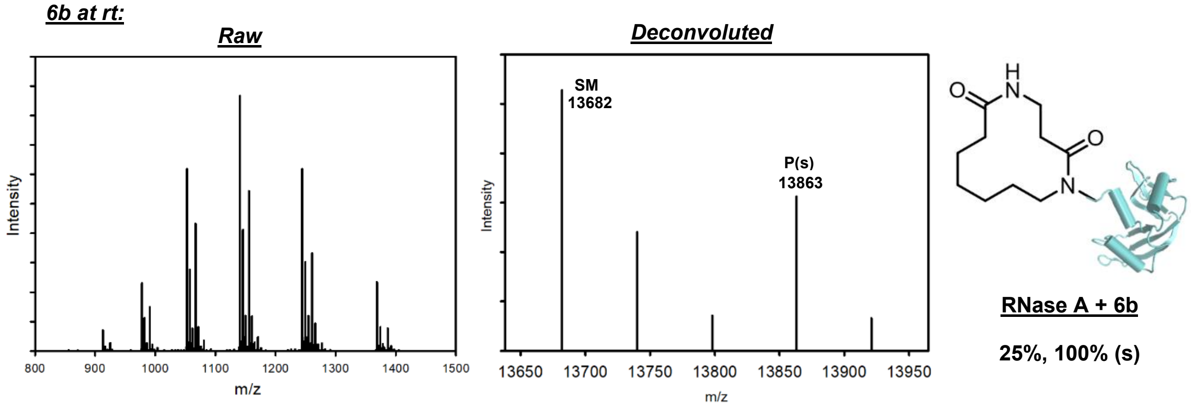

MS (ESI$^+$): [**SM**+H]$^+$ found 13682, calculated 13681; [**SM**+58]$^+$ found 13739; [**SM**+116]$^+$ found 13978; [**P(s)**+H]$^+$ found 13863, calculated 13862; [**P(s)**+58]$^+$ found 13920.

Table S1: Tabulated data for Figure 2D – Screening conditions for RNase A bioconjugation with imide **6b.**

Entry	pH	Equivalents of **6b**	Selectivity (%)	Conversion (%)	Symbol
1	6.0	5	0	0	green ▲
2	6.0	10	100	10	green ■
3	6.0	20	100	28	green ●
4	6.0	50	86	64	green ◆
5	6.5	5	100	11	orange ▲
6	6.5	10	100	20	orange ■
7	6.5	20	100	38	orange ●
8	6.5	50	82	79	orange ◆
9	7.4	5	100	25	red ▲
10	7.4	10	100	46	red ■
11	7.4	20	81	79	red ●
12	7.4	50	53	100	red ◆
13	8.0	5	100	39	blue ▲
14	8.0	10	80	66	blue ■
15	8.0	50	47	93	blue ◆

[a] Reactions were run at 50 µM RNase A at room temperature, 1000 rpm for 19 h before an aliquot was taken and analysed by LC-MS (for full experimental and LC-MS details, see SI, section S18).

6c at rt (pH 6.0, 20 eq.): *was modified using General Procedure D3 at rt.*

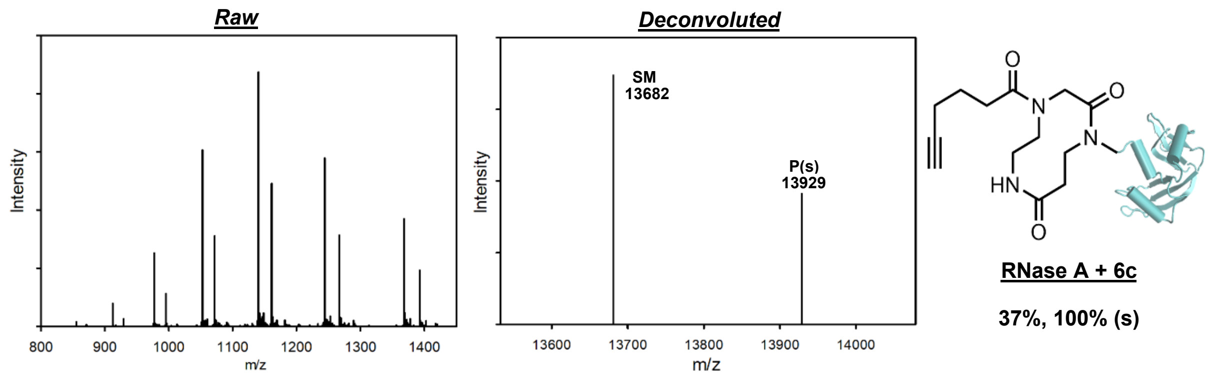

MS (ESI$^+$): [**SM**+H]$^+$ found 13682, calculated 13681; [**P(s)**+H]$^+$ found 13929, calculated 13929.

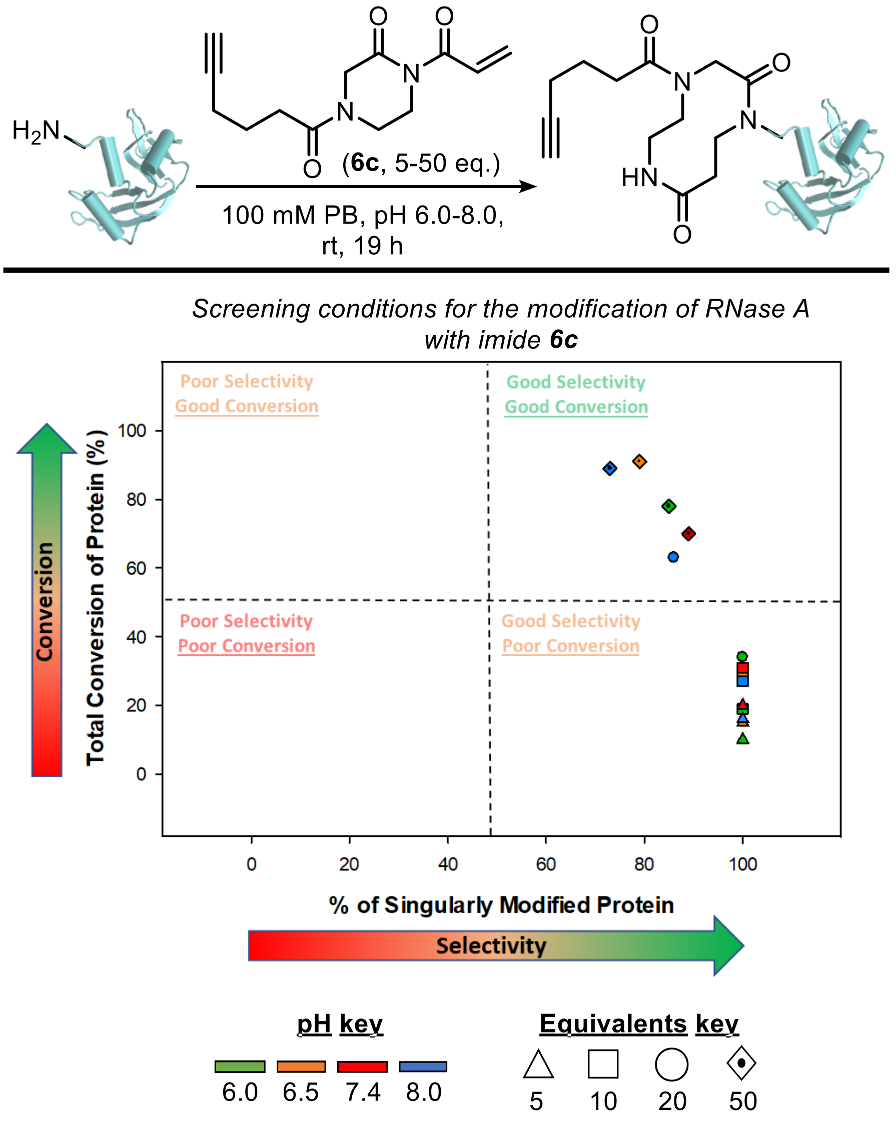

Figure S24: Screening conditions for RNase A bioconjugation using imide **6c**: Reactions were run at 50 µM of protein at pH 6.0, 6.5, 7.4 and 8.0 with 5, 10, 20 or 50 equivalents of imide **6c:** 6.7 µL of protein (~150 µM stock in 0.1 M PB, pH 7.4), 4 µL of PB (0.5 M stock, pH adjusted accordingly) and 7.3 µL of HPLC-grade water is added to 2 µL of imide **6c** (stock adjusted accordingly in DMSO) and incubated at room temperature, 1000 rpm for ~19 h before an aliquot is taken and analysed by LC-MS.

Table S2: Tabulated data for Figure S21 – Screening conditions for RNase A bioconjugation with imide **6c.**

Entry	pH	Equivalents of **6c**	Selectivity (%)	Conversion (%)	Symbol
1	6.0	5	100	10	▲
2	6.0	10	100	19	■
3	6.0	20	100	34	●
4	6.0	50	85	78	◆
5	6.5	5	100	15	▲
6	6.5	10	100	30	■
7	6.5	50	79	91	◆
8	7.4	5	100	20	▲
9	7.4	10	100	31	■
10	7.4	20	89	70	●
11	7.4	50	78	100	◆
12	8.0	5	100	16	▲
13	8.0	10	100	27	■
14	8.0	20	86	63	●
15	8.0	50	73	89	◆

[a] Reactions were run at 50 µM RNase A at room temperature, 1000 rpm for 19 h before an aliquot was taken and analysed by LC-MS (for full experimental and LC-MS details, see SI, section S18).

Myoglobin: *was modified using General Procedure D1 at 37 ºC and rt.*

6b at 37 ºC:

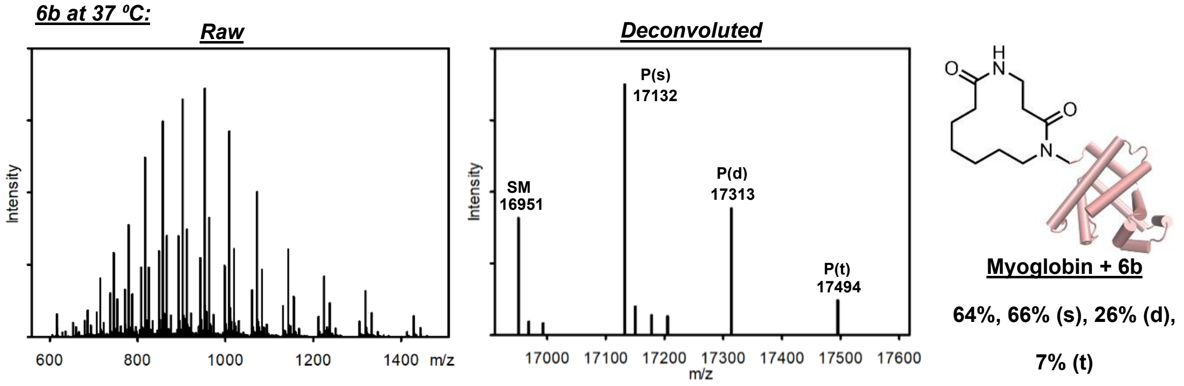

MS (ESI$^+$): [**SM**+H]$^+$ found 16951, calculated 16951; [**SM**+H_2O+H]$^+$ found 16969, calculated 16969; [**SM**+MeCN+H]$^+$ found 16993, calculated 16993; [**P(s)**+H]$^+$ found 17132, calculated 17132; [**P(s)**+H_2O+H]$^+$ found 17150, calculated 17150; [**P(s)**+MeCN+H]$^+$ found 17177, calculated 17177; [**P(s)**+DMSO+H]$^+$ found 17205, calculated 17205; [**P(d)**+H]$^+$ found 17313, calculated 17313; [**P(t)**+H]$^+$ found 17494, calculated 17494.

6b at rt:

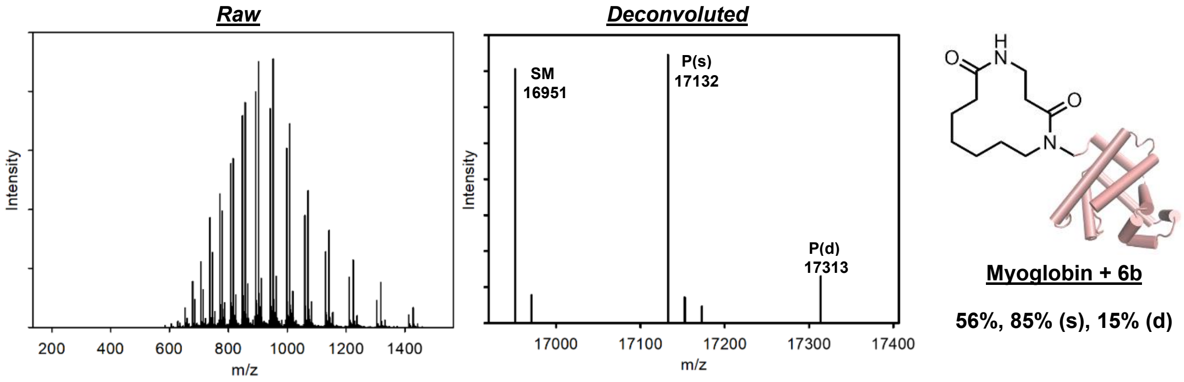

MS (ESI$^+$): [**SM**+H]$^+$ found 16951, calculated 16951; [**SM**+H_2O+H]$^+$ found 16970, calculated 16969; [**P(s)**+H]$^+$ found 17132, calculated 17132; [**P(s)**+H_2O+H]$^+$ found 17152, calculated 17150; [**P(s)**+MeCN+H]$^+$ found 17173, calculated 17174; [**P(d)**+H]$^+$ found 17313, calculated 17313.

Cytochrome C: *was modified using General Procedure D1 at 37 ºC and rt.*

6b at 37 ºC:

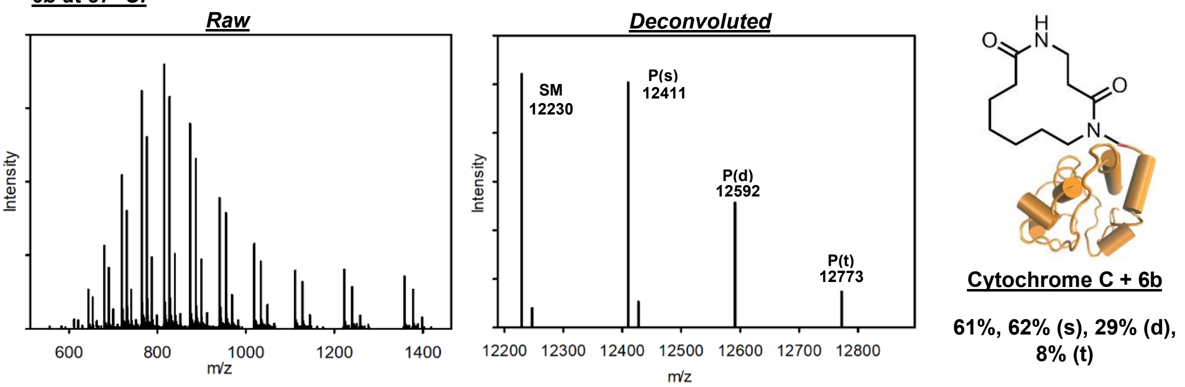

MS (ESI$^+$): [**SM**+H]$^+$ found 12230, calculated 12327; [**SM**+H_2O+H]$^+$ found 12247, calculated 12345; [**P(s)**+H]$^+$ found 12411, calculated 12508; [**P(s)**+H_2O+H]$^+$ found 12428, calculated 12526; [**P(d)**+H]$^+$ found 12592, calculated 12689; [**P(t)**+H]$^+$ found 12773, calculated 12870.

6b at rt:

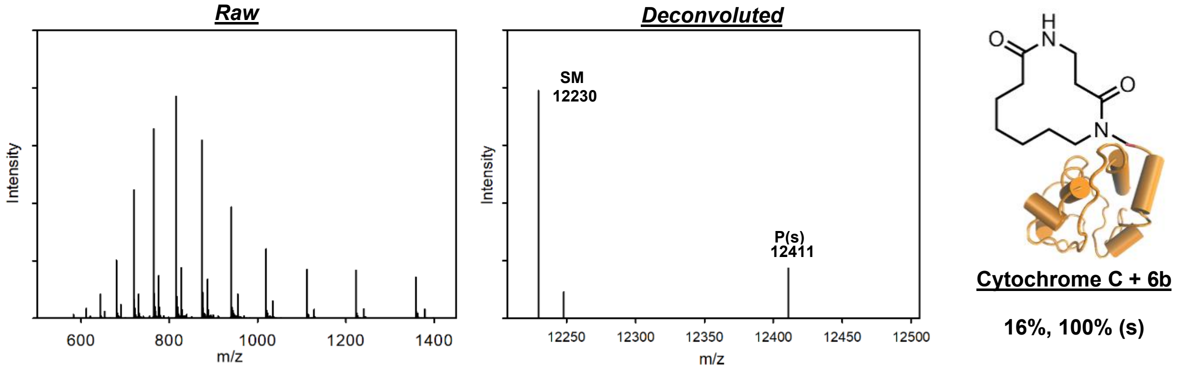

MS (ESI$^+$): [**SM**+H]$^+$ found 12230, calculated 12327; [**SM**+H_2O+H]$^+$ found 12247, calculated 12345; [**P(s)**+H]$^+$ found 12411, calculated 12508.

JVZ-007: *was modified using General Procedure D4 at rt.*

6c at rt (pH 6.0, 200 eq.):

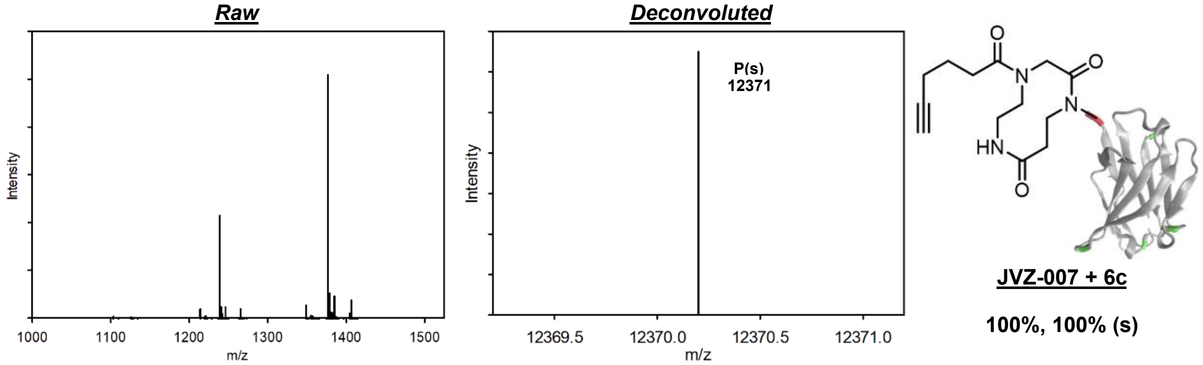

MS (ESI$^+$): [**P(s)**+H]$^+$ found 12371, calculated 12372.

Table S3: Tabulated data for Figure 3A – Screening conditions for JVZ-007 bioconjugation with imide **6c.**

Entry	pH	Equivalents of **6c**	Selectivity (%)	Conversion (%)	Symbol
1	6.0	5	0	0	▲
2	6.0	10	0	0	■
3	6.0	20	0	0	●
4	6.0	50	100	34	◆
5	6.0	100	100	85	★
6	6.0	200	100	100	▼
7	6.5	5	0	0	▲
8	6.5	10	0	0	■
9	6.5	20	0	0	●
10	6.5	50	100	65	◆
11	6.5	100	100	100	★
12	6.5	200	100	100	▼
13	7.4	5	0	0	▲
14	7.4	10	0	0	■
15	7.4	20	100	22	●
16	7.4	50	100	74	◆
17	7.4	100	100	100	★
18	7.4	200	100	100	▼
19	8.0	5	0	0	▲
20	8.0	10	0	0	■
21	8.0	20	100	12	●
22	8.0	50	100	47	◆
23	8.0	100	100	100	★
24	8.0	200	100	100	▼

[a] Reactions were run at 50 µM JVZ-007 at room temperature, 1000 rpm for 19 h before an aliquot was taken and analysed by LC-MS (for full experimental and LC-MS details, see SI, section S18).

Determination of conjugate stability

JVZ-007 was modified with reagent **6c** (100 equiv.) in accordance with **General Procedure D4**, on a 50 µM scale. The reaction outcome was determined by LC-MS after 19 h before the crude reaction mixture was placed directly into a 3.5 kDa MWCO dialysis membrane and incubated in LC-MS water (45 mL) at 37 °C with agitation (800 rpm) for 24 h. After this time, the sample was analysed by LC-MS to determine the stability of the conjugates.

<u>Before incubation:</u> *100% conversion, 100% single modification*

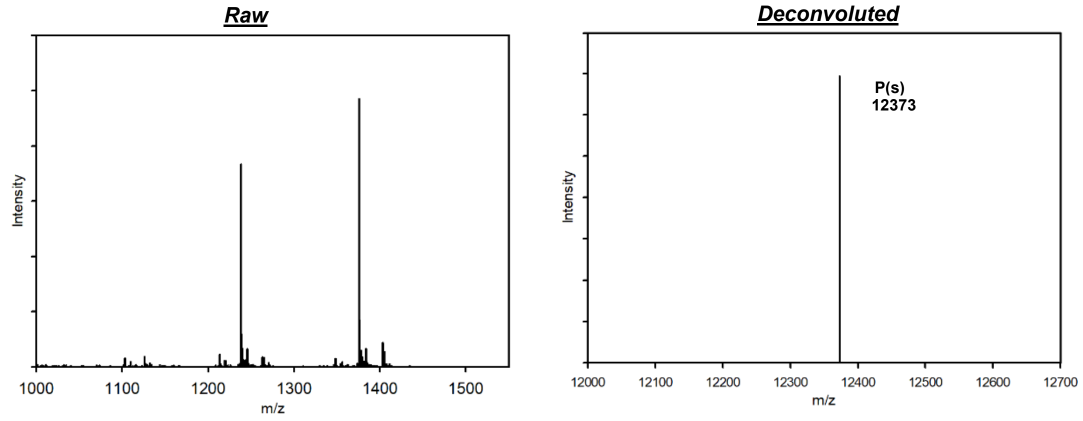

MS (ESI$^+$): [**P(s)**+H]$^+$ found 12373, calculated 12372.

<u>After incubation:</u> *100% conversion, 100% single modification*

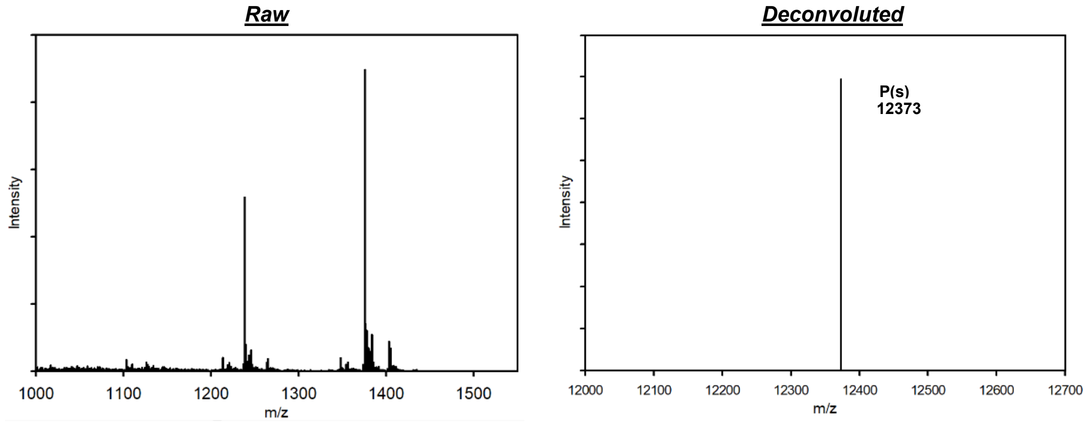

MS (ESI$^+$): [**P(s)**+H]$^+$ found 12373, calculated 12372.

CCL5: *was modified using General Procedure D5 at rt.*

6a at rt (pH 6.0, 50 eq.):

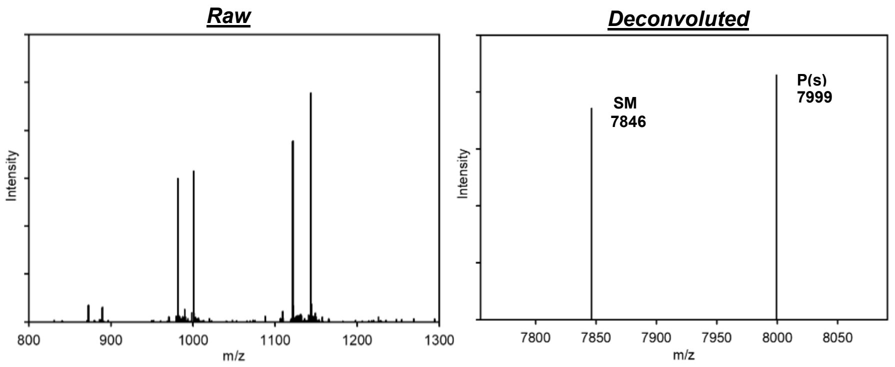

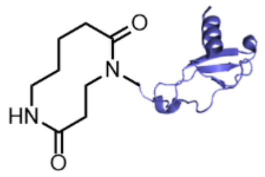

CCL5 + 6A

54%, 100% (s)

MS (ESI$^+$): [**SM**+H]$^+$ found 7846, calculated 7846; [**P(s)**+H]$^+$ found 7999, calculated 7999.

6a at rt (pH 6.5, 75 eq.):

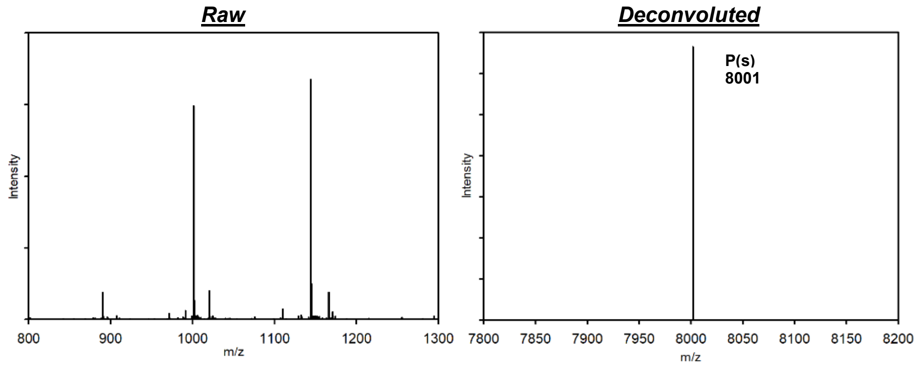

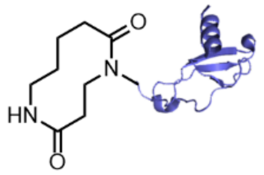

CCL5 + 6A

100%, 100% (s)

MS (ESI$^+$): [**P(s)**+H]$^+$ found 8002, calculated 7999.

6d at rt (pH 6.0, 50 eq.):

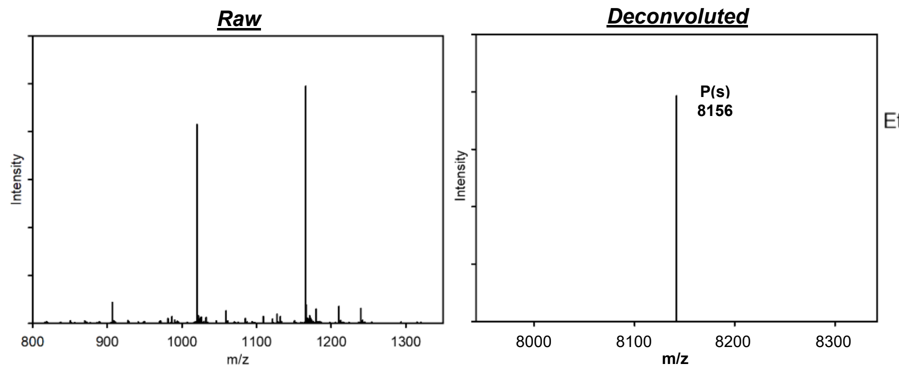

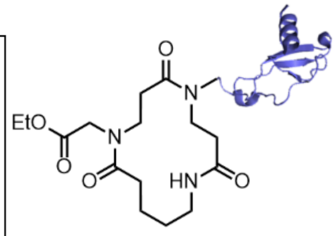

CCL5 + 6d

100%, 100% (s)

MS (ESI$^+$): [**P(s)**+H]$^+$ found 8156, calculated 8156.

6e at rt (pH 6.0, 50 eq.):

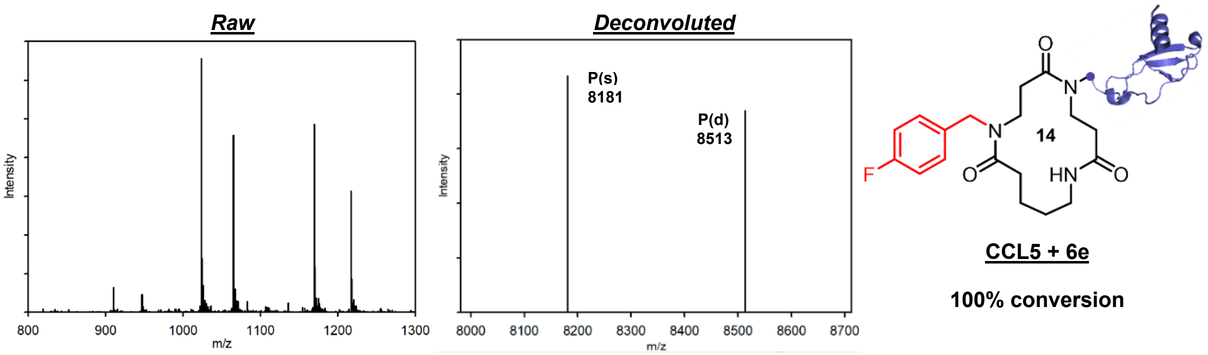

MS (ESI$^+$): [**P(s)**+H]$^+$ found 8181, calculated 8178.

SI10. Methods for functional testing of modified proteins

i) CCL5 and macrocycle derivatives activation and down-modulation of CCR5.

Endocytosis assay: CHO-CCR5 cells detached in PBS-10mM EDTA were resuspended at 2 × 106 cells/mL in in Binding Medium (BM: 1xRPMI w/o phenol red plus 1% BSA pH 7.0), pre-labelled in suspension with the anti-CCR5 specific monoclonal antibody MC-5 known to bind the N-terminus of the receptor without affecting chemokine binding[6] and used at 5µg/ml in BM before treating cells with 100 nM of chemokine tools (in-house produced wild-type CCL5[6] or the indicated macrocycle derivative) for up to 30 mins at 37°C, keeping one control of untreated cells (medium). Following washes in fresh BM, cells were fixed overnight with a 1% formaldehyde solution in PBS before detecting MC-5-bound CCR5 remaining at the surface using an Alexa Fluor 633 anti-Mouse IgG fluorescent secondary antibody (Invitrogen) in FACS Buffer (PBS, 1% FCS with 0.05% sodium azide). Cell-associated Mean Fluorescence Intensity was measured with a CytoFLEX S flow cytometer (Beckman Coulter) and results are expressed as percentage of signal from cells prior to treatment[6].

Recycling assay: CHO-CCR5 cells were detached, resuspended in BM and treated with chemokine tools as above to trigger downmodulation. After 30 mins, cells were washed and resuspended in fresh BM supplemented with 800 nM of the CCR5 antagonist TAK-779 for up to 60 mins further incubation at 37°C, keeping on ice a sample for reference of downmodulation. TAK-779 is used as a displacer of ligands remaining bound to CCR5, avoiding further cycles of receptor internalisation[7]. Collected cells kept on ice were then fixed in 1% formaldehyde before labelling 1 h at RT with Alexa Fluor 488-coupled MC-5 at 5µg/ml in FACS Buffer and samples analysis with a CytoFLEX S, as above. Results are expressed as percentage of fluorescence signal recovered compared to cells taken at the end of the downmodulation step.

Phosphorylation assay: CHO-CCR5 cells treated with chemokine tools as above for up to 30 mins, aliquots removed at the indicated time and fixed in equal volume of 6% formaldehyde solution in PBS for 20 min. Cells were washed 1x with FACs buffer and kept in ice-cold methanol −80 °C until staining. Cells removed from −80 °C were washed 3x with FACs buffer and incubated with the Anti-CCR5

E11/19-APC (IgG1) specific for CCR5 phosphor Ser349 purchased from Biolegends (as recommended by the manufacturer) for 1 h 4 °C.[6] Cells then washed 3 x in FACs buffer and resuspended in 200 µL FACs buffer and analysed using a CytoFLEX S. Data expressed as mean ± SEM of the stated number of experiments and were analysed with GraphPad Prism v9.4.1. The indicated statistical tests were used, where appropriate.

ii) Immunofluorescence staining and microscopy.

- **CCR5 cell distribution:** CHO-CCR5 cells were seeded onto coverslips at least 48 h before experiment. Cells were incubated in BM alone or supplemented with 100 nM of CCL5 or the indicated macrocycle derivative for 30 mins at 37 °C. Cells were fixed in PBS with 3% formaldehyde for 20 mins at RT and quenched in NH4Cl (50 mM) O/N at 4 °C. Samples were permeabilised with a solution of PBS containing 0.05% saponin and 1% FBS for 30 mins at RT, stained with MC-5 (5µg/ml) for 1 h at RT detected using an anti-mouse Alexa fluor 488 (4 µg/mL) 1 h at RT. After washes in PBS/saponin and PBS, coverslips were mounted using mowiol containing DAPI[6]. Samples were examined with Zeiss LSM980 confocal microscope (63x/1.4 oil), single confocal sections were acquired, and images analysed using Zeiss ZEN Lite software.

- **Using the modified JVZ nanobody:**

<u>Tissue microarrays (TMA)</u>. TMA slides were de-waxed, and antigen retrieval was carried out by pressure cooking in Tris-EDTA (pH 9.0). Slides were blocked using 1% BSA in TBS for 1 h. JVZ nanobody was added to the slides at a final concentration of 0.2 µM in TBS and incubated for 1 h at RT. Slides were mounted using mounting medium containing DAPI (Abcam, #ab104139) and imaged using a Leica fluorescence microscope. Images were processed using LASX software.

<u>Prostate cancer cell-line</u>: LNCaP cells were grown on coverslips. Cells were washed 2x with cold PBS, then blocked with 1% human serum in PBS (HS/PBS) for 30 min at 4°C. The blocking solution was removed and the JVZ conjugate was added at a 1:100 dilution in HS/PBS. Coverslips were incubated for 3 h at 4 °C. Cells were washed 3 x 5 min with PBS then fixed with 3% formaldehyde in PBS for 15 minutes at room temperature. After 3 x PBS washes, coverslips were mounted using Mowiol containing DAPI at a 1:5000 dilution. Coverslips were imaged using a Zeiss Axioplan fluorescence microscope and processed using image J. Images were taken at an exposure time of 3s.

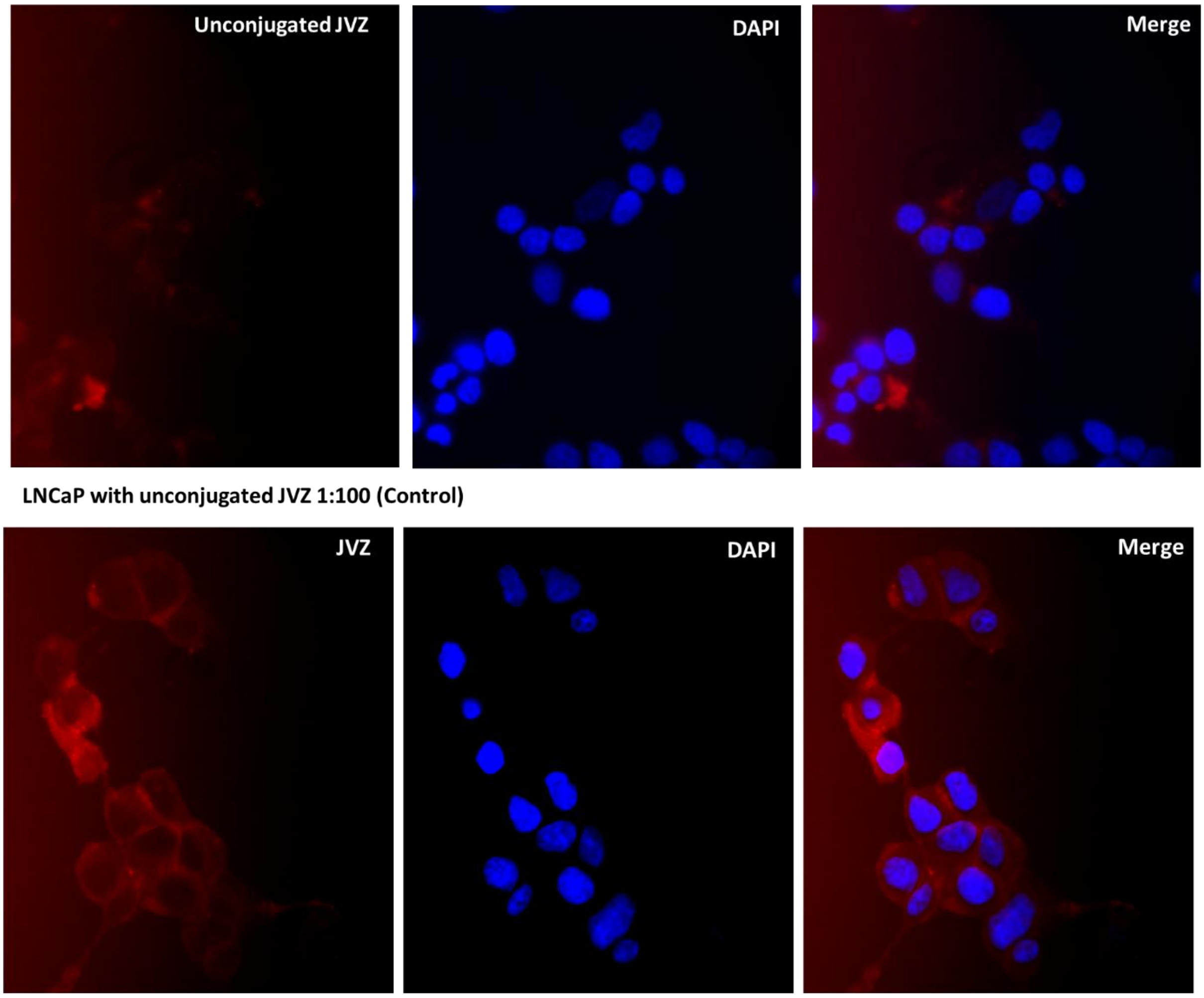

LNCaP with JVZ 1:100

Figure S25: Fluorescent microscopy images of PSMA on LNCaP prostate cancer cells visualised using fluorescently labelled JVZ-007.

SI11. NMR Spectra

Acryloyl-piperidin-2-one (6a)

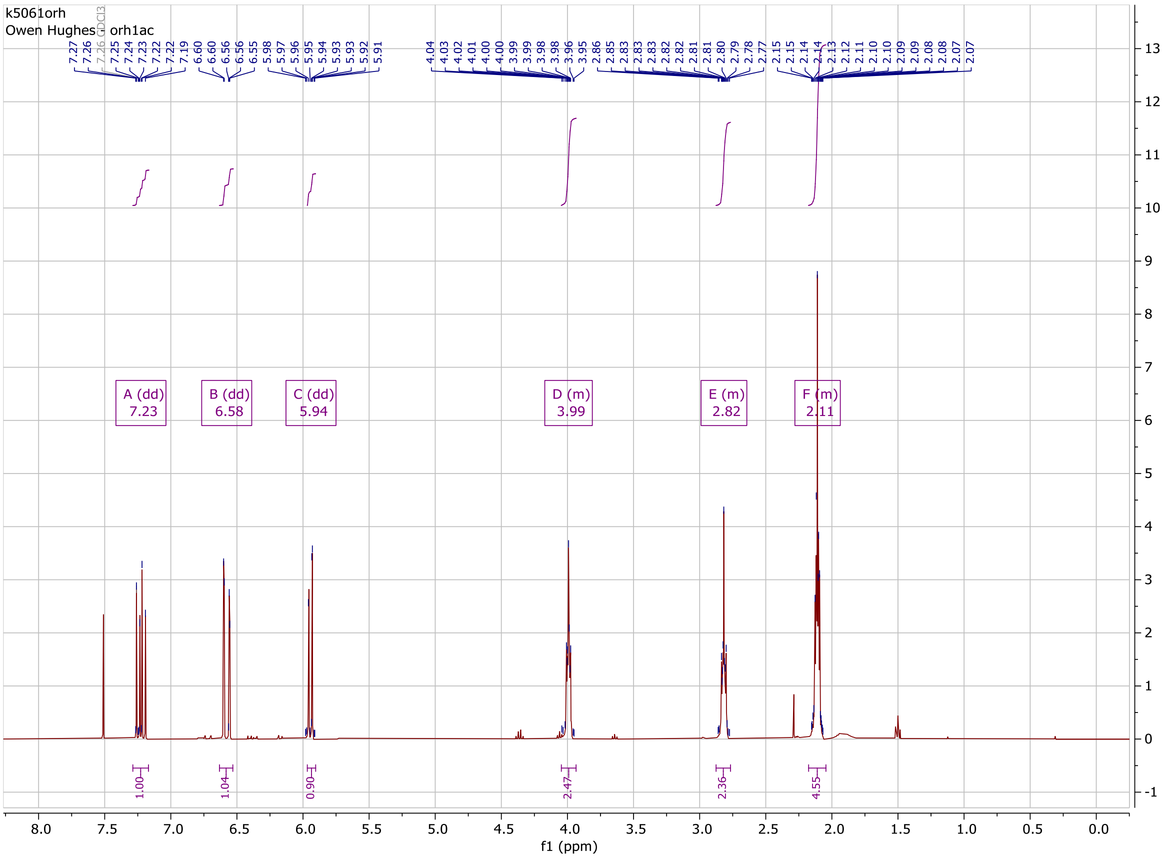

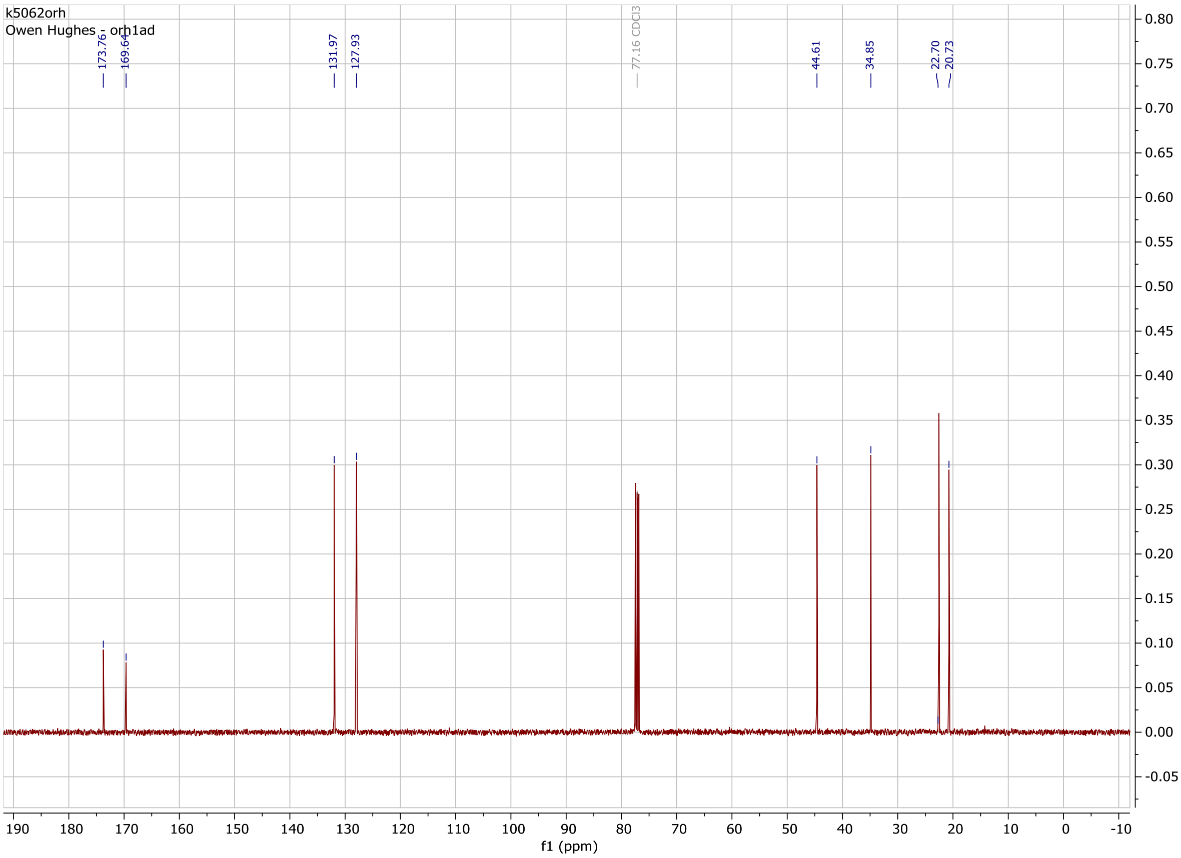

1-Acryloylazocan-2-one (6b)

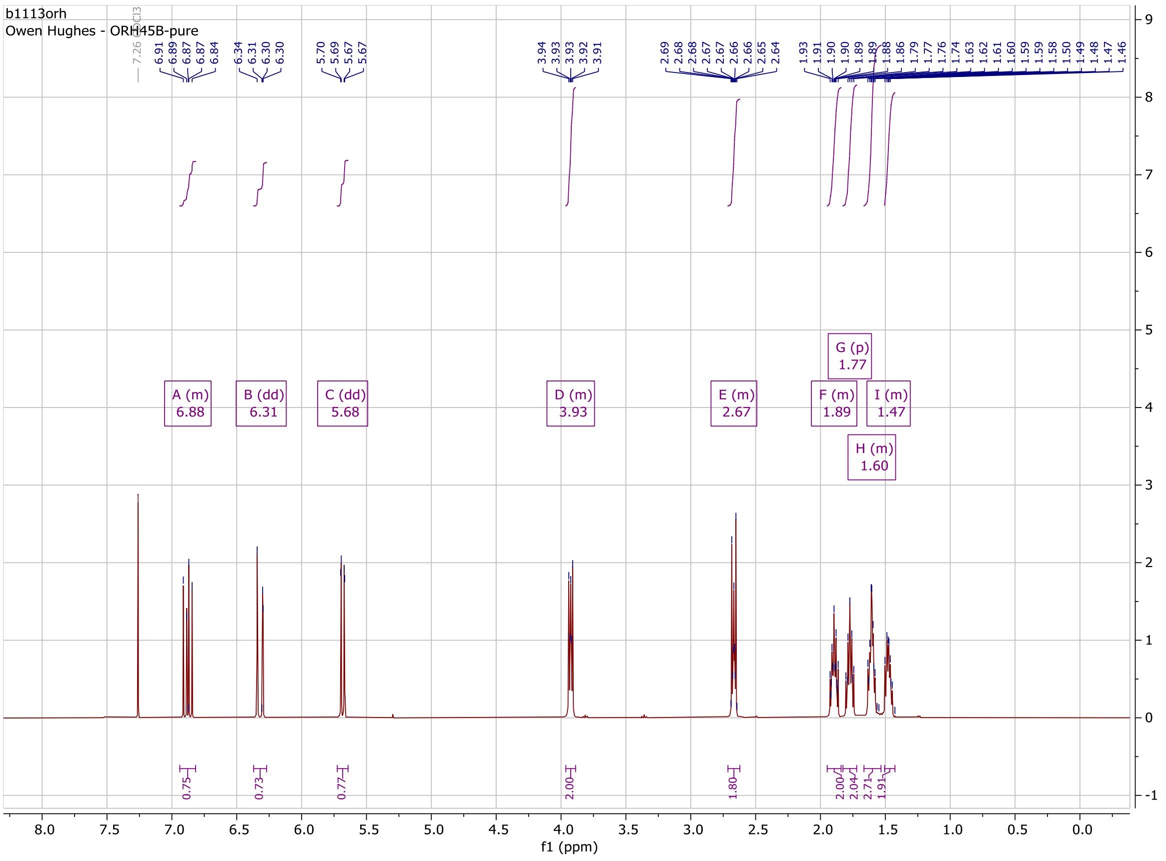

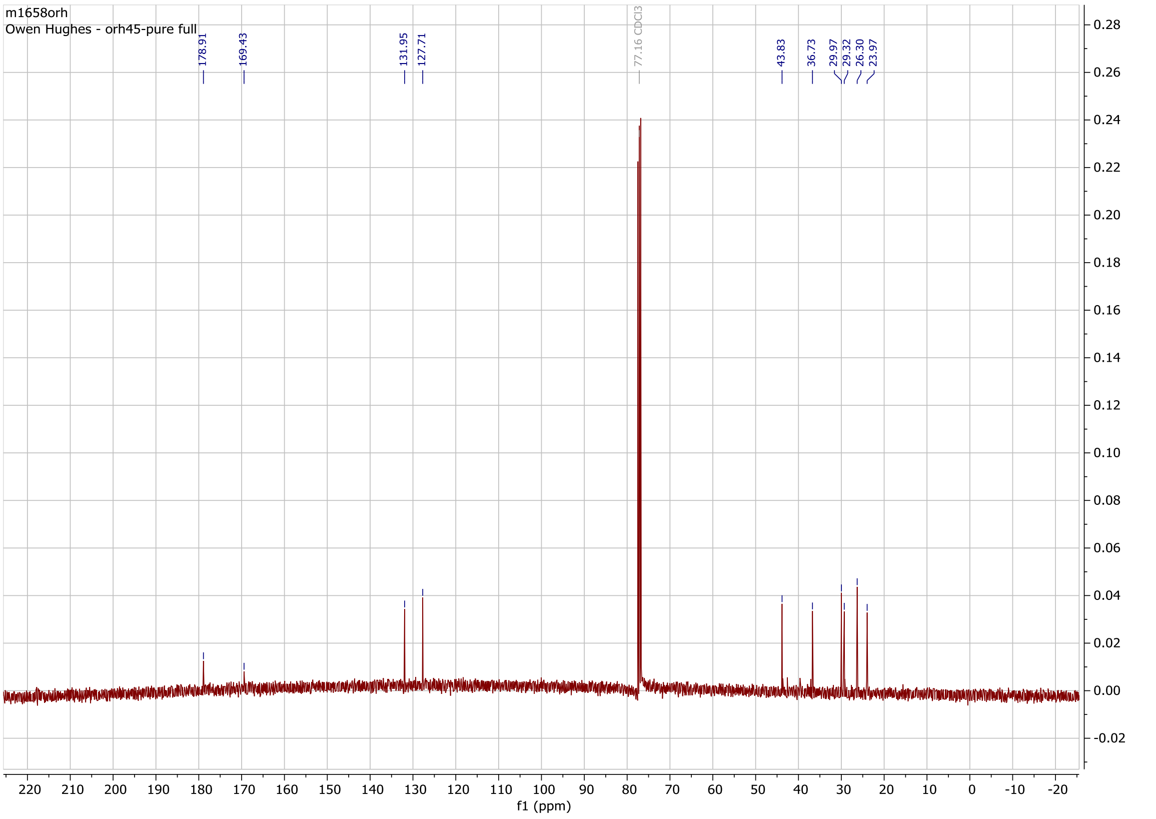

4-(Hex-5-ynoyl)piperazin-2-one (S1) - In solution in $CDCl_3$, the compound exists as a mixture of two rotamers in an approximately 5:4 (A:B) ratio:

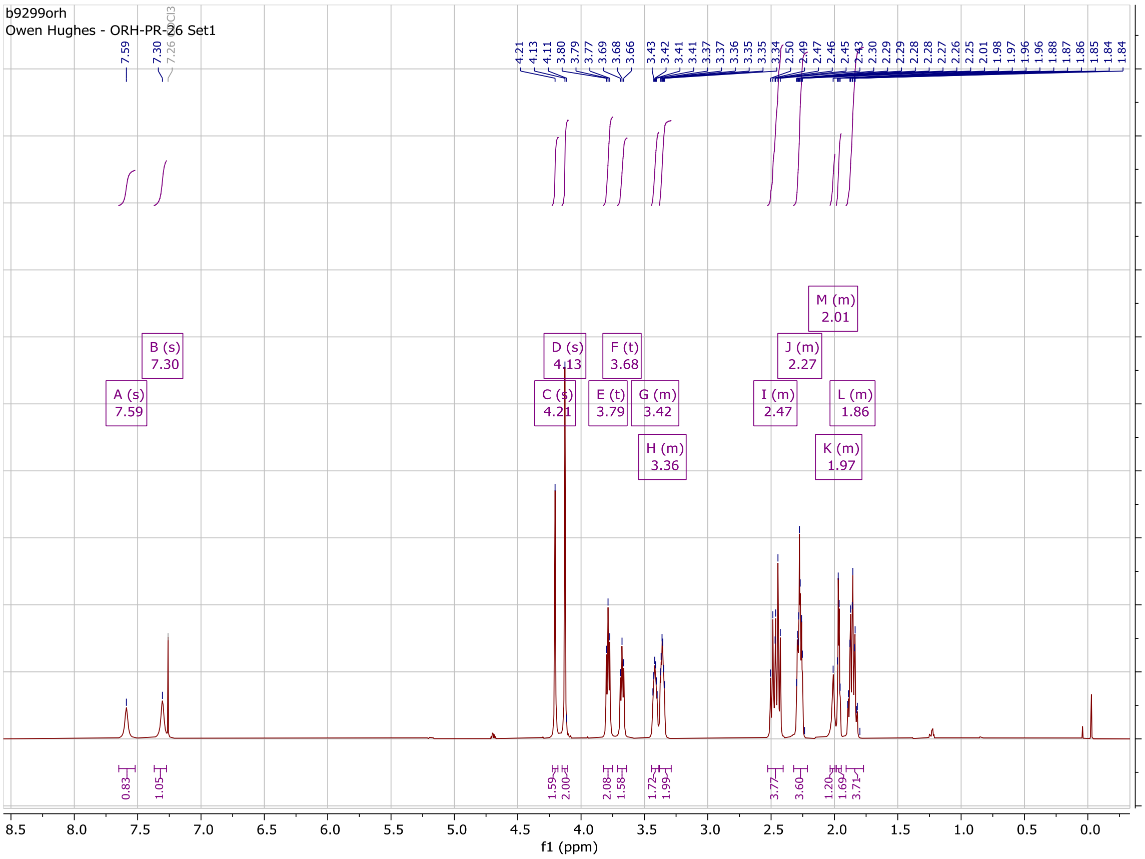

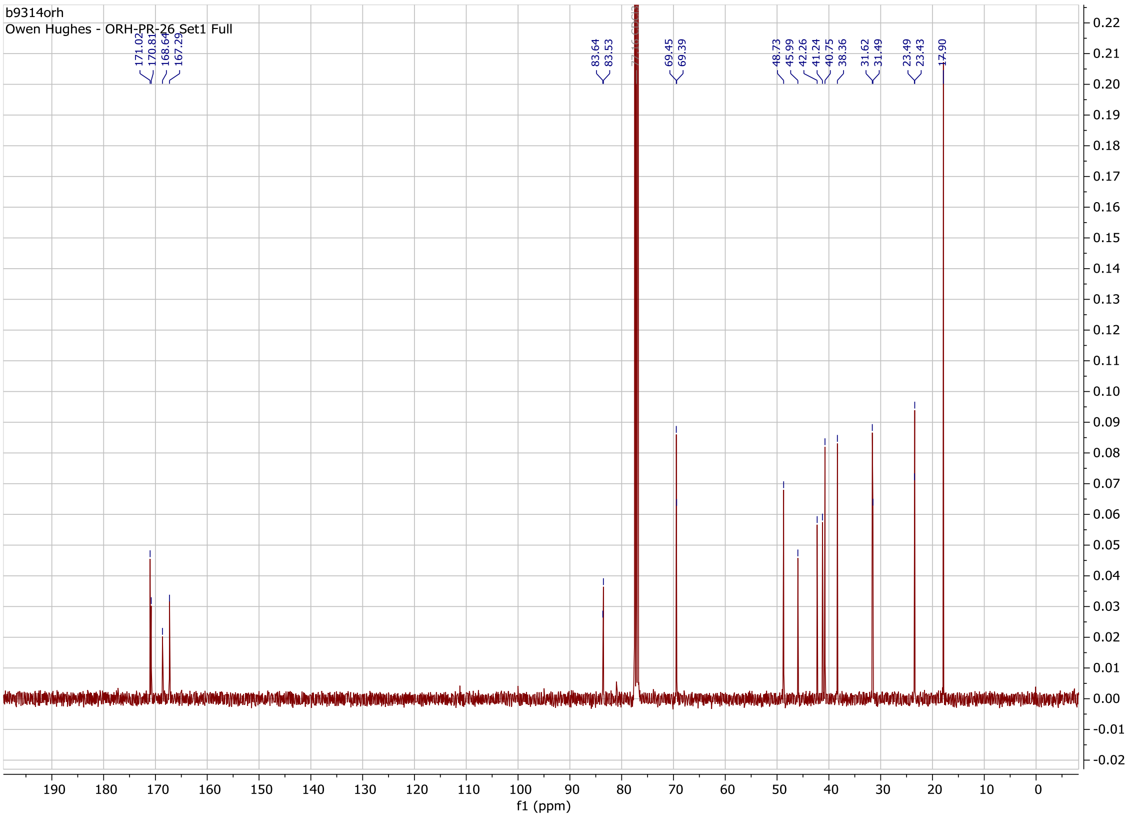

1-Acryloyl-4-(hex-5-ynoyl)piperazine-2-one (6c) - In solution in $CDCl_3$, the compound exists as a mixture of two rotamers in an approximately 4:3 (A:B) ratio:

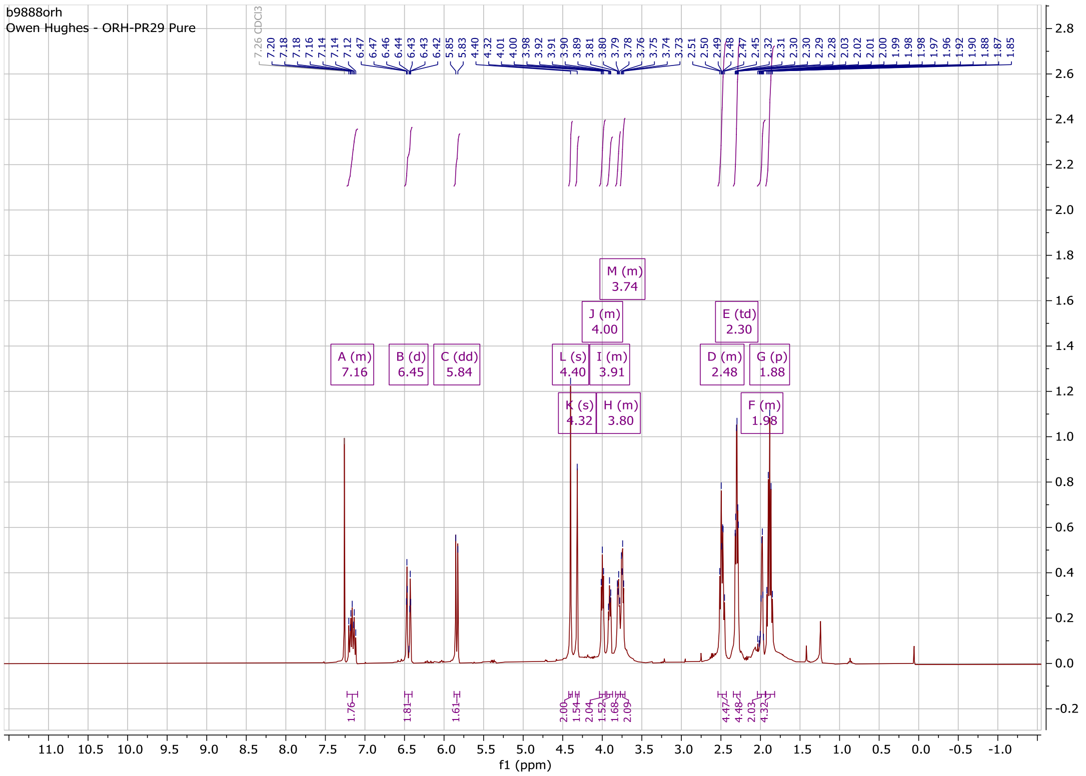

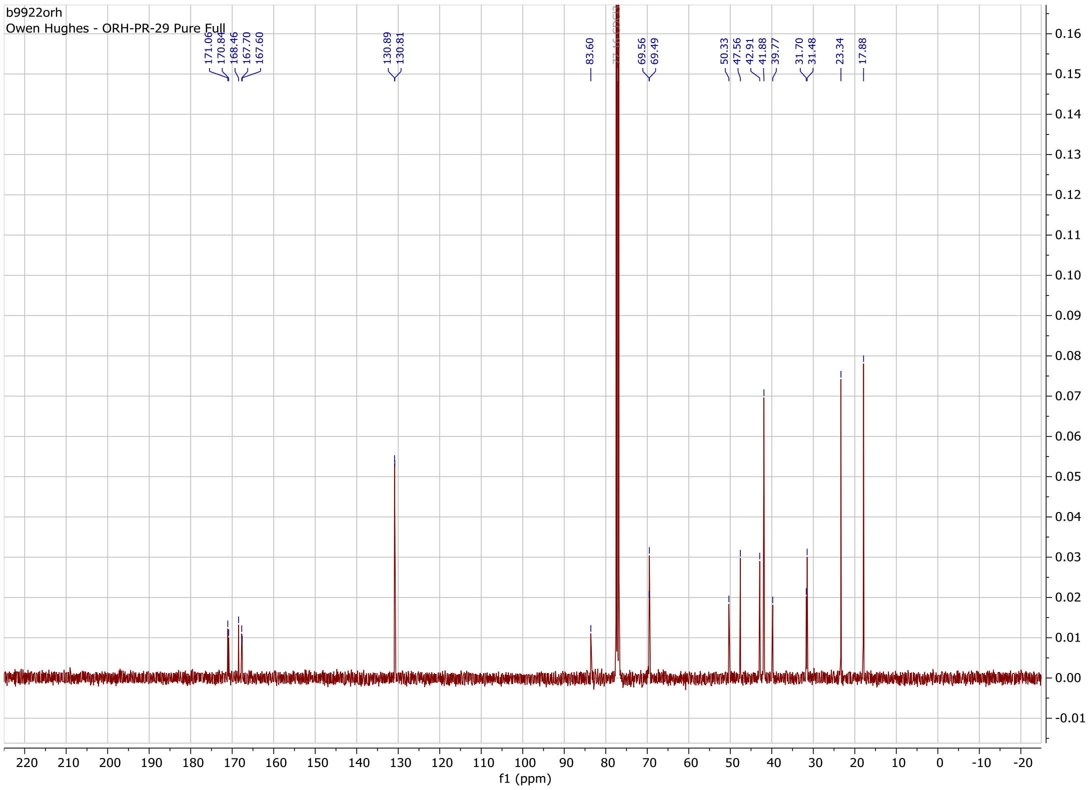

Ethyl 2-(4,10-dioxo-1,5-diazecan-1-yl) acetate (7a)

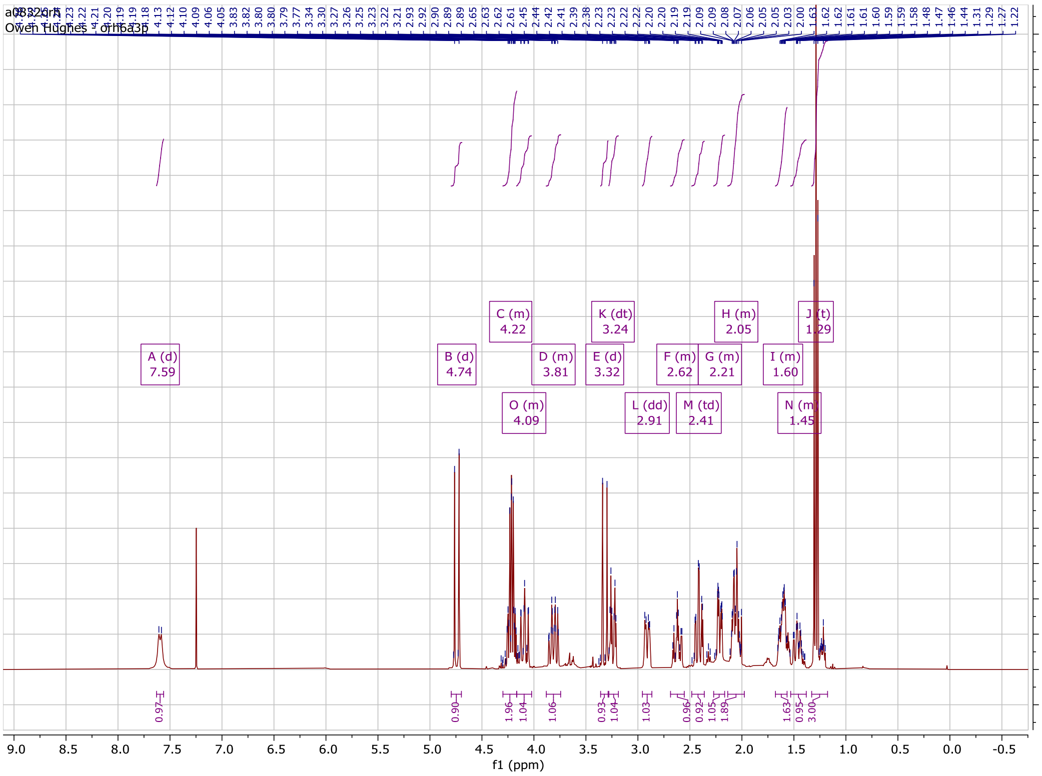

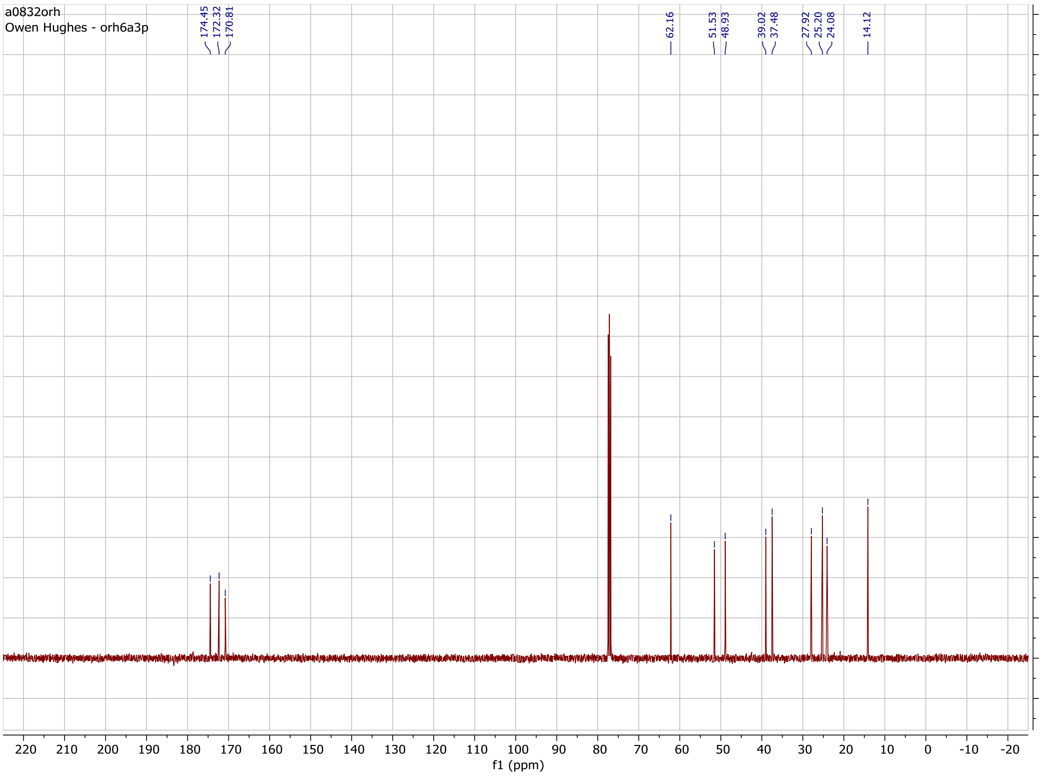

Ethyl 2-(5-acryloyl-4,10-dioxo-1,5-diazecan-1-yl)acetate (6d)

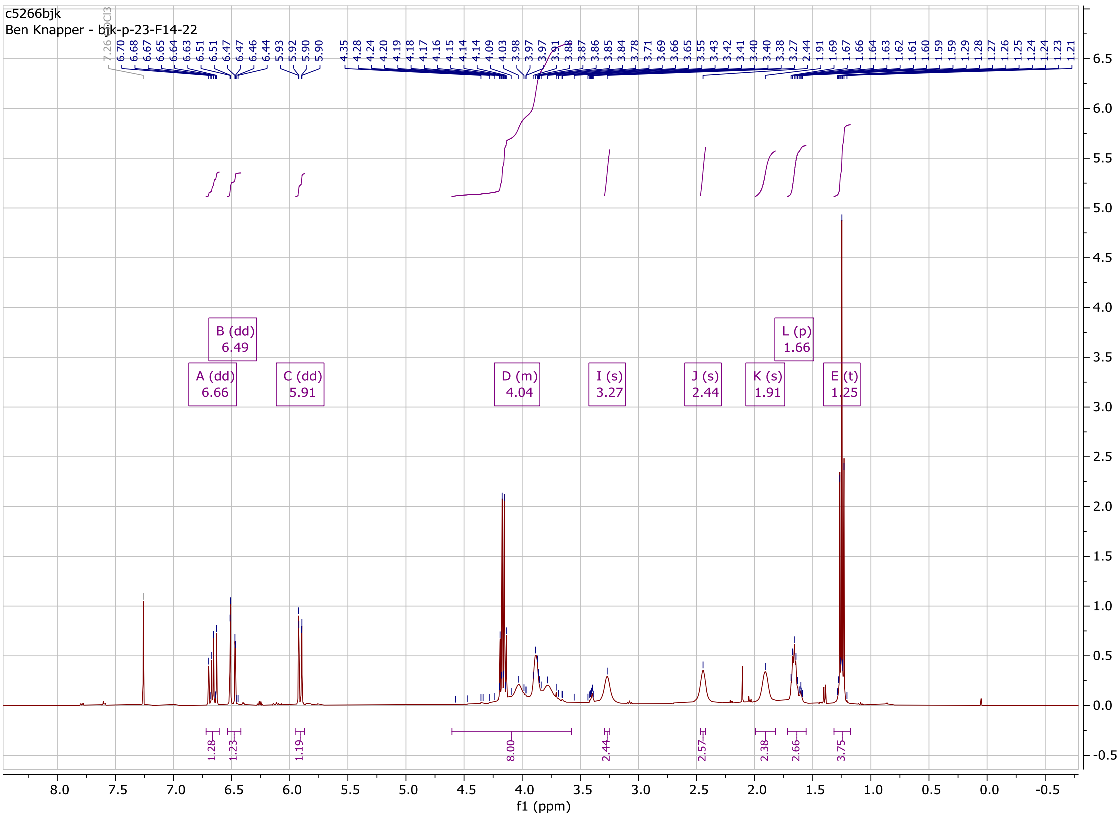

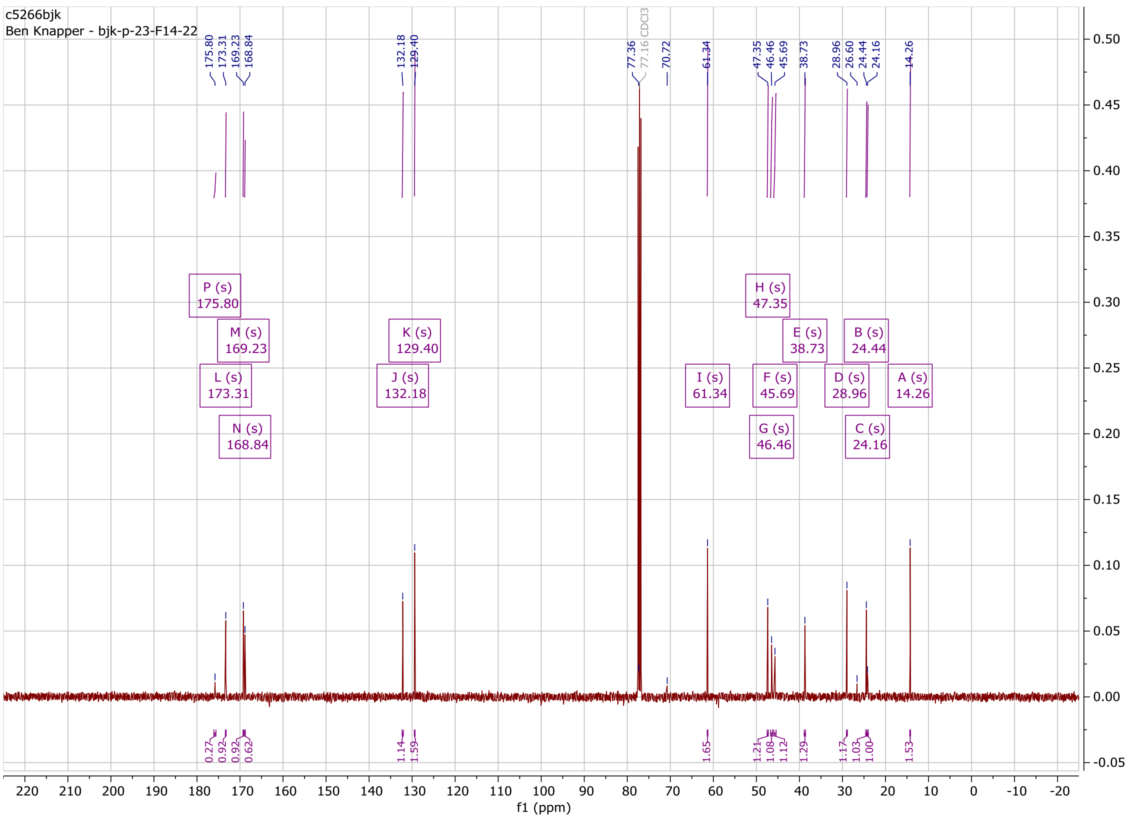

5-(4-Fluorobenzyl)-1,5-diazecane-2,6-dione (S2) - In solution in $CDCl_3$, the compound exists as a mixture of two rotamers in an approximately 10:1 (A:B) ratio:

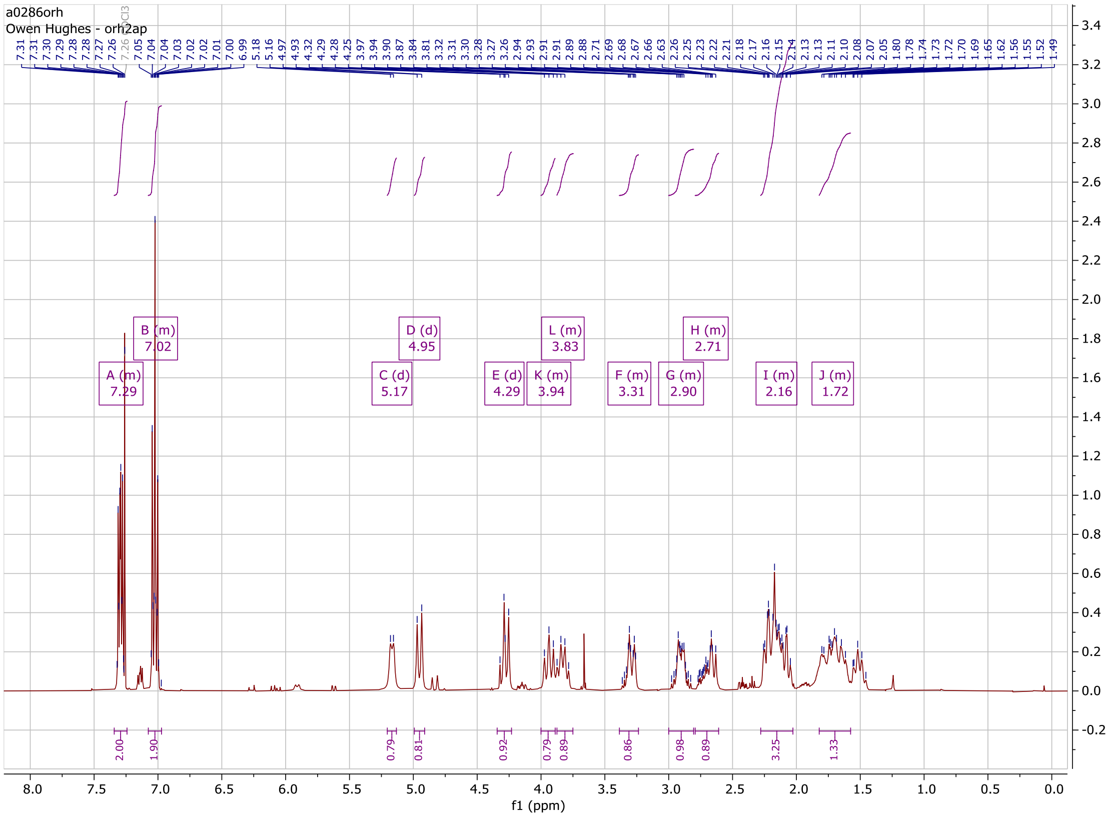

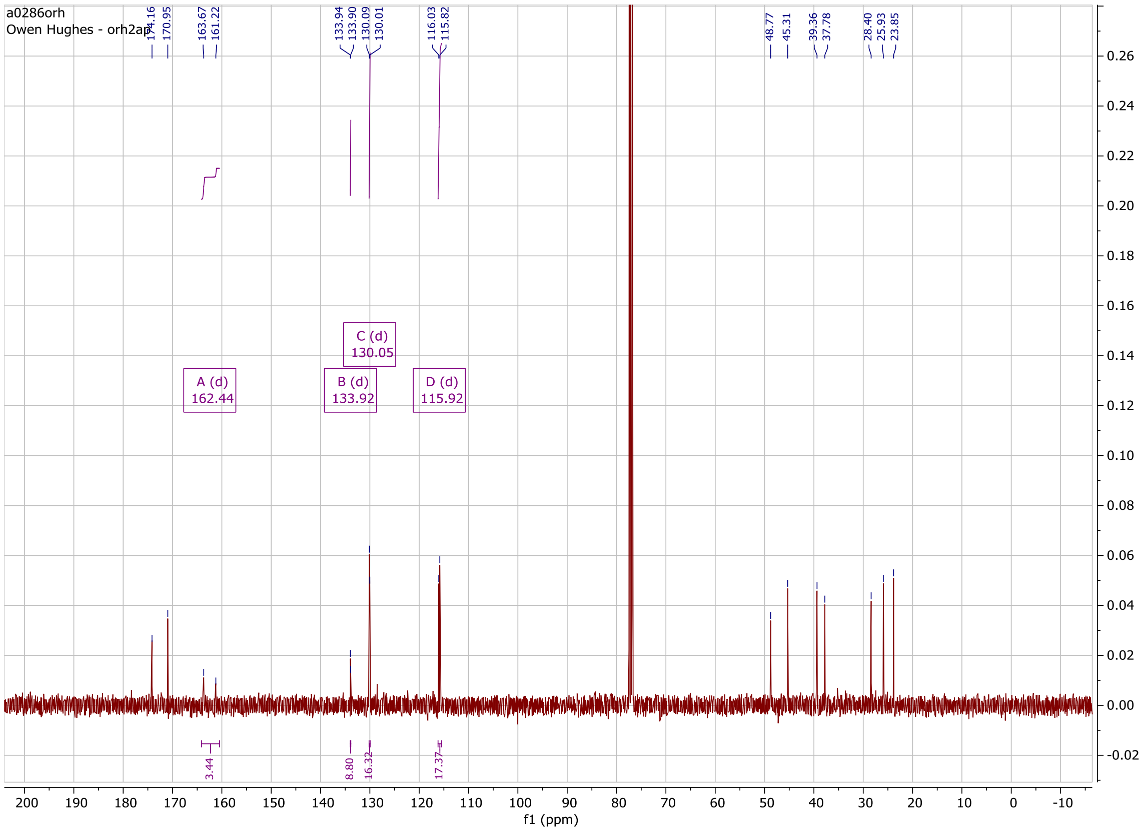

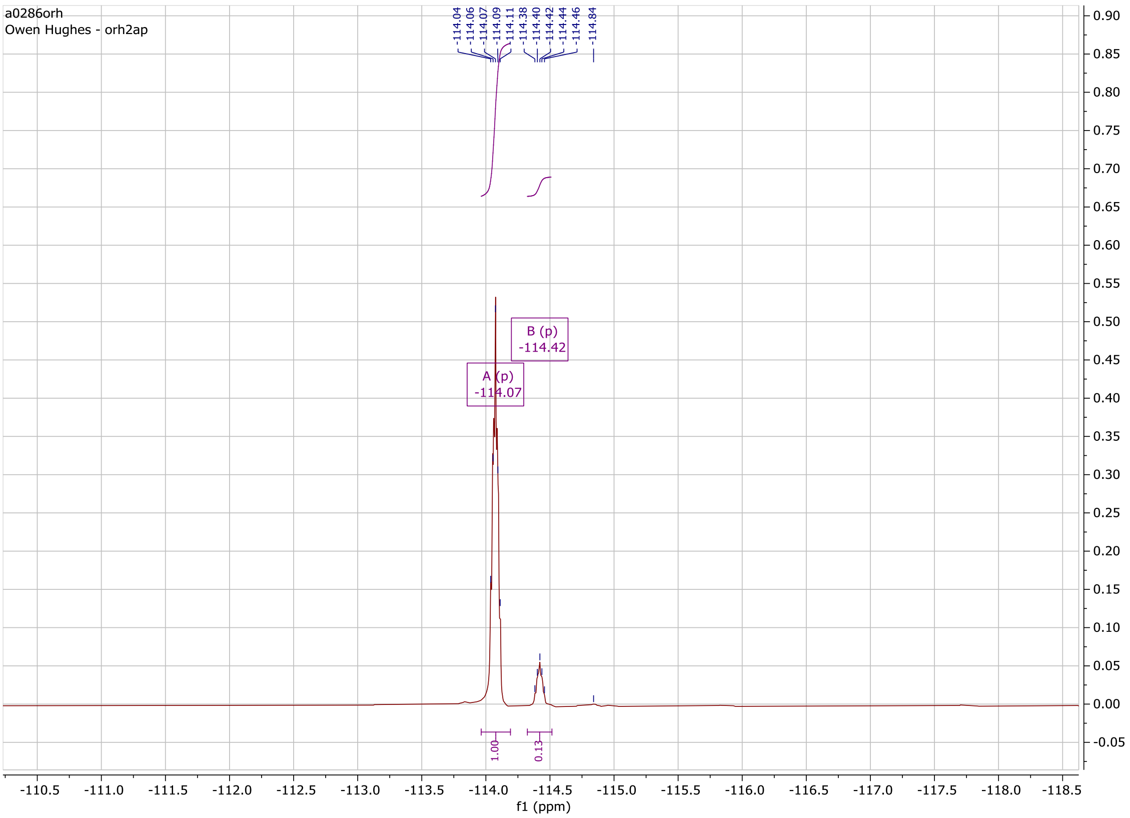

a0286orh
Owen Hughes - orh2ap
-114.04
-114.06
-114.07
-114.09
-114.11
-114.38
-114.40
-114.42
-114.44
-114.46
-114.84
B (p)
-114.42
A (p)
-114.07
1.00
0.13
f1 (ppm)

1-Acryloyl-5-(4-fluorobenzyl)-1,5-diazecane-2,6-dione (6e) - In solution in $CDCl_3$, the compound exists as a mixture of two rotamers in an approximately 10:1 (A:B) ratio:

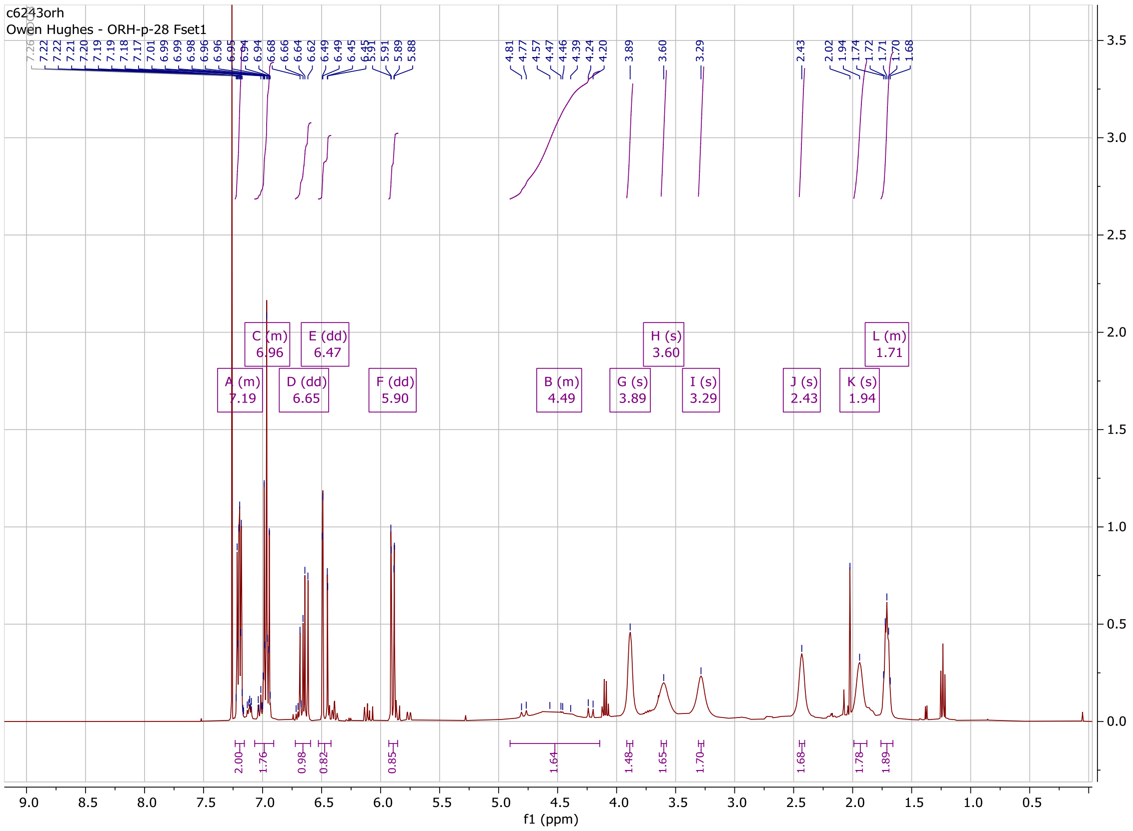

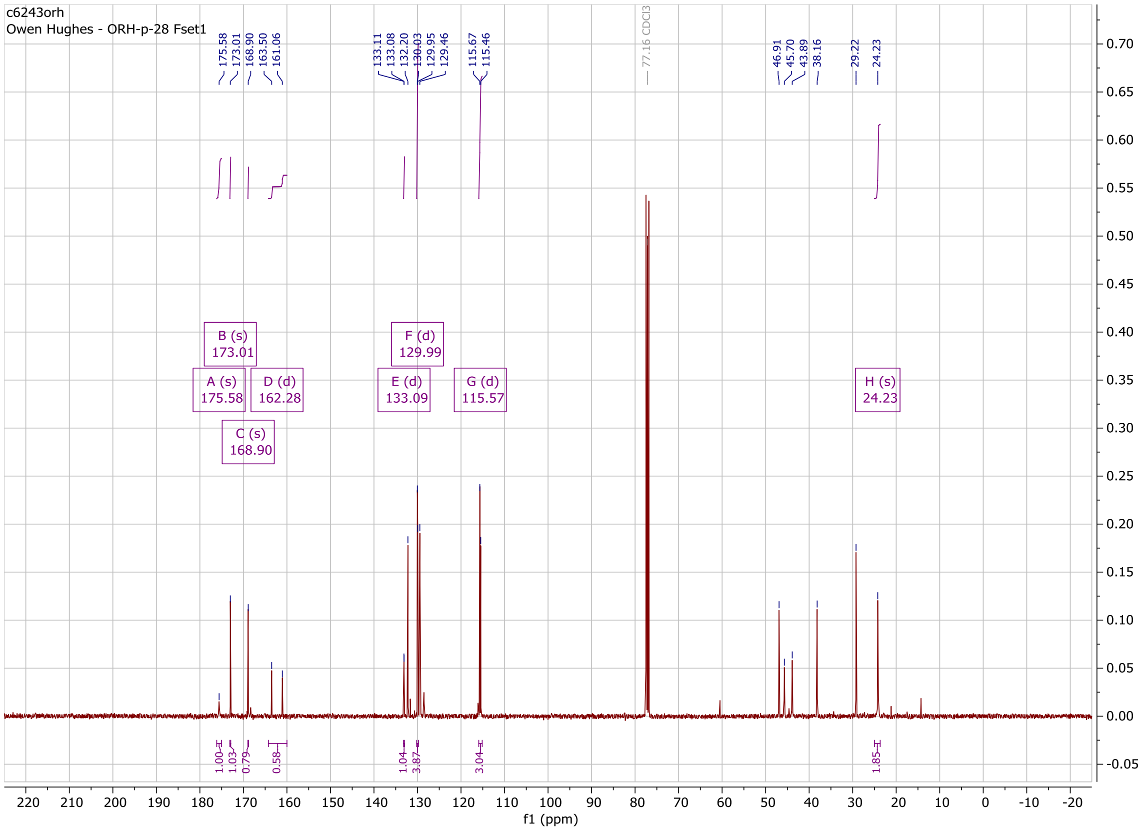

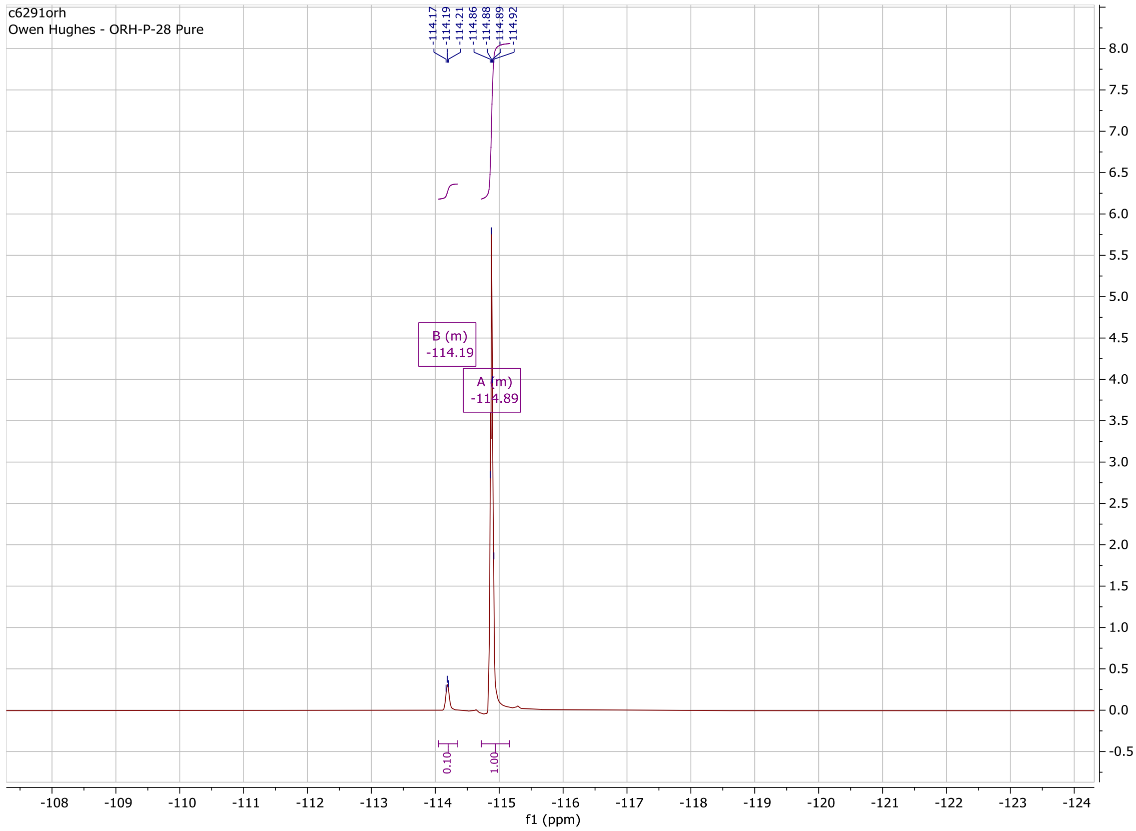
c6291orh
Owen Hughes - ORH-P-28 Pure
-114.17
-114.19
-114.21
-114.86
-114.88
-114.89
-114.92
B (m)
-114.19
A (m)
-114.89
0.10
1.00
f1 (ppm)

Ethyl 2-(4,10-dioxo-1,5-diazecan-1-yl) propanoate (7b) - In solution in $CDCl_3$, the compound exists as a mixture of two rotamers in an approximately 25:11 (A:B) ratio:

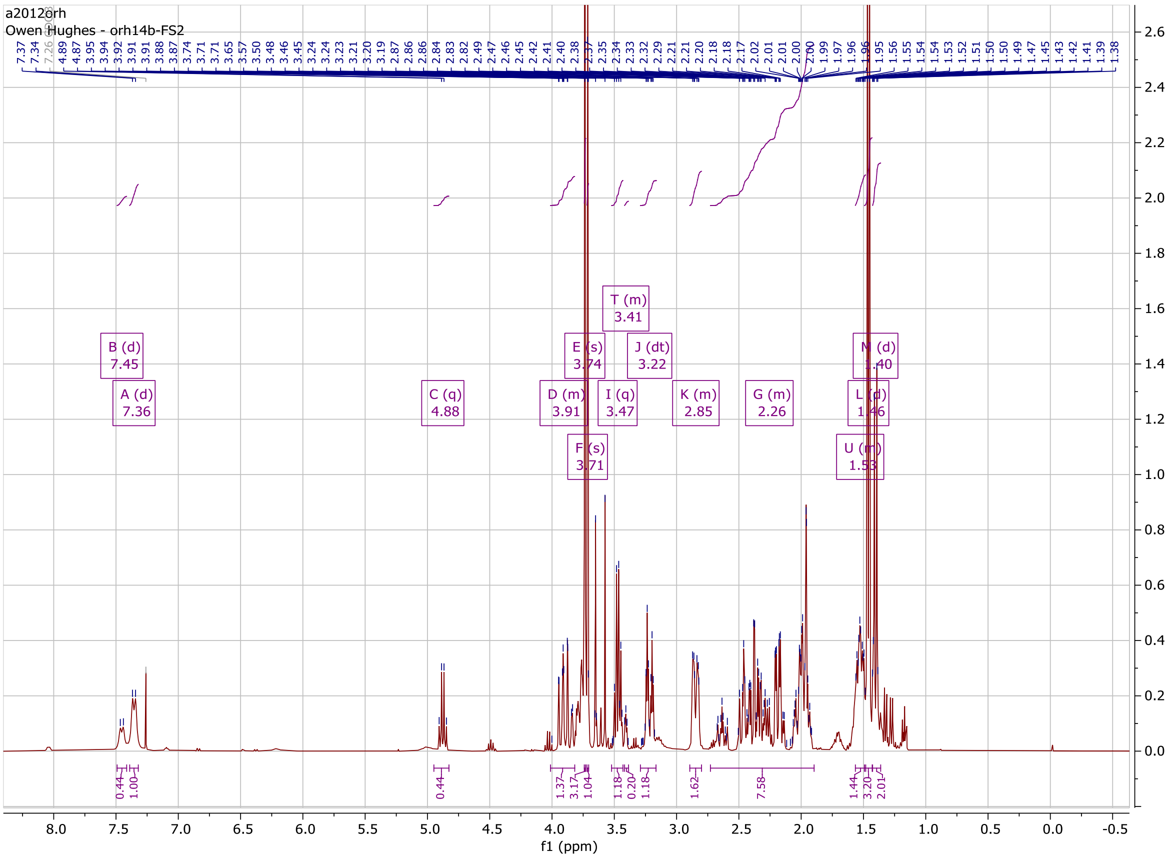

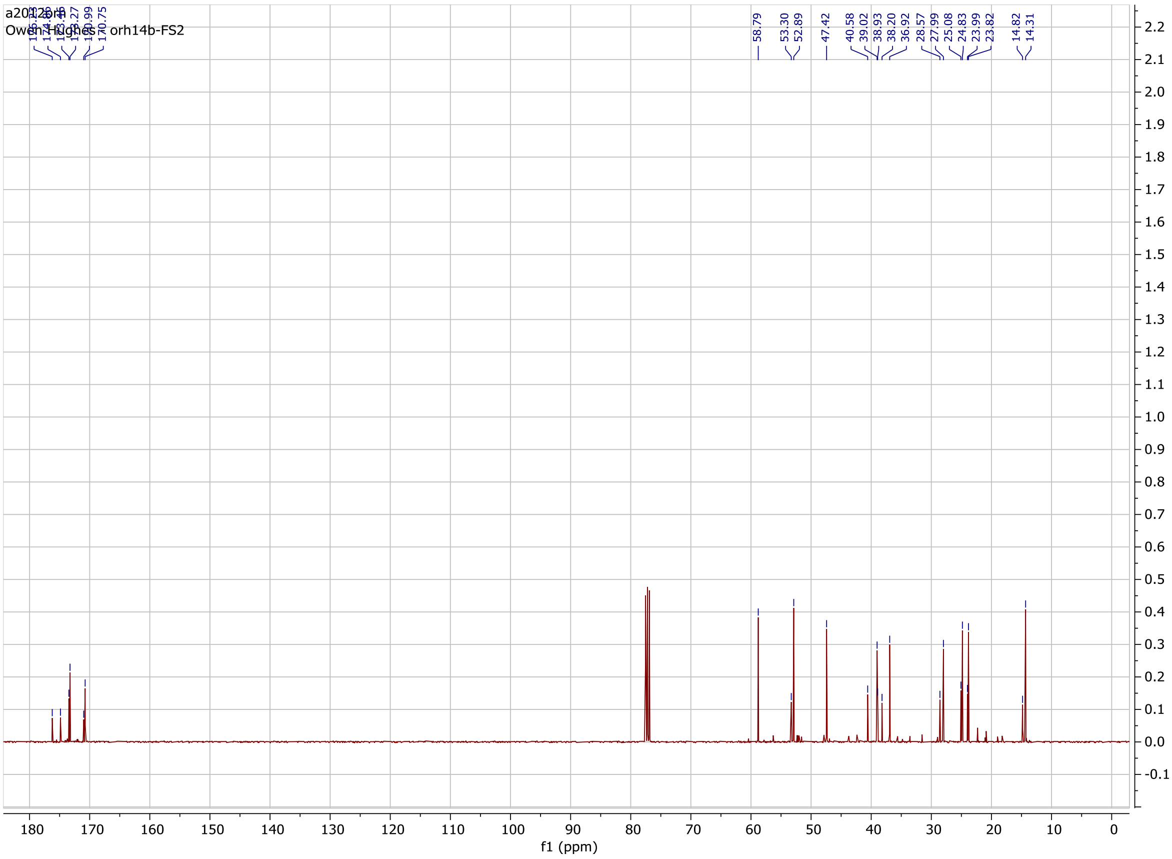

Methyl 2-(4,10-dioxo-1,5-diazecan-1-yl)-3-methylbutanoate (7c)

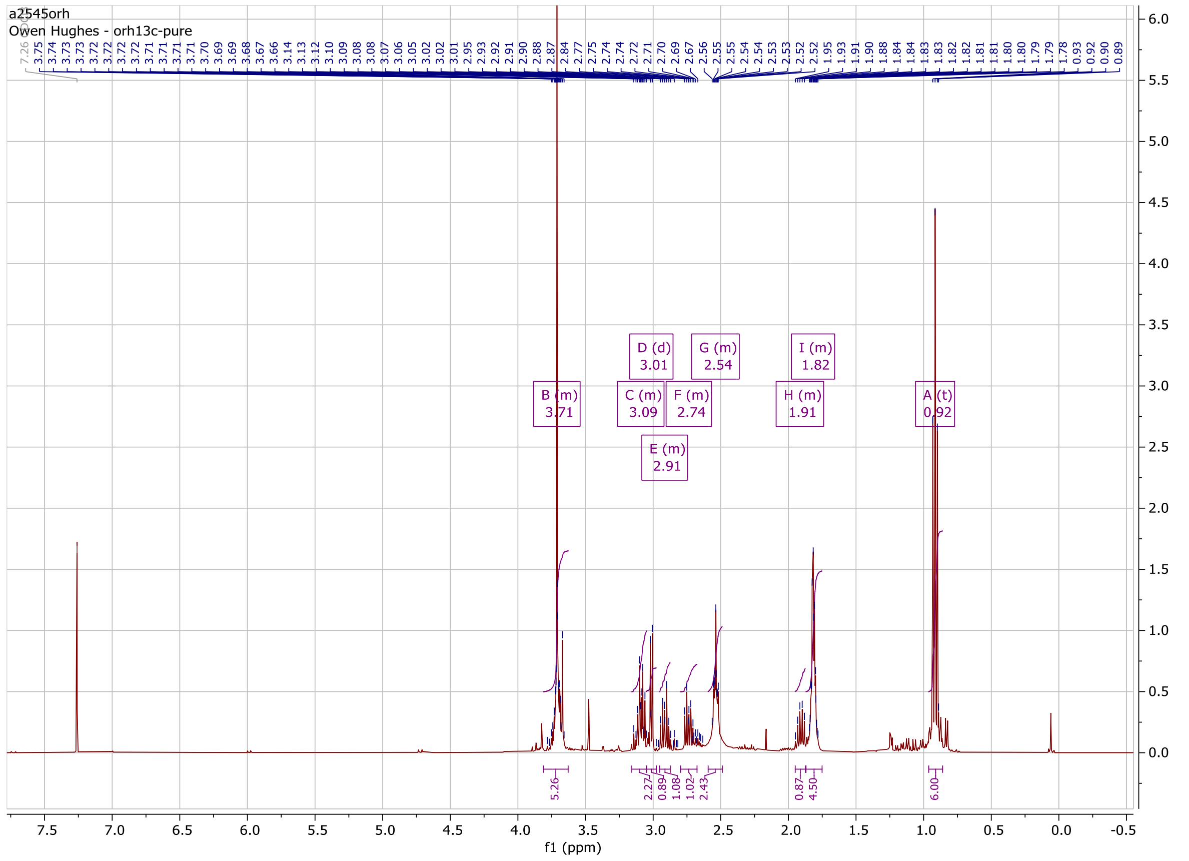

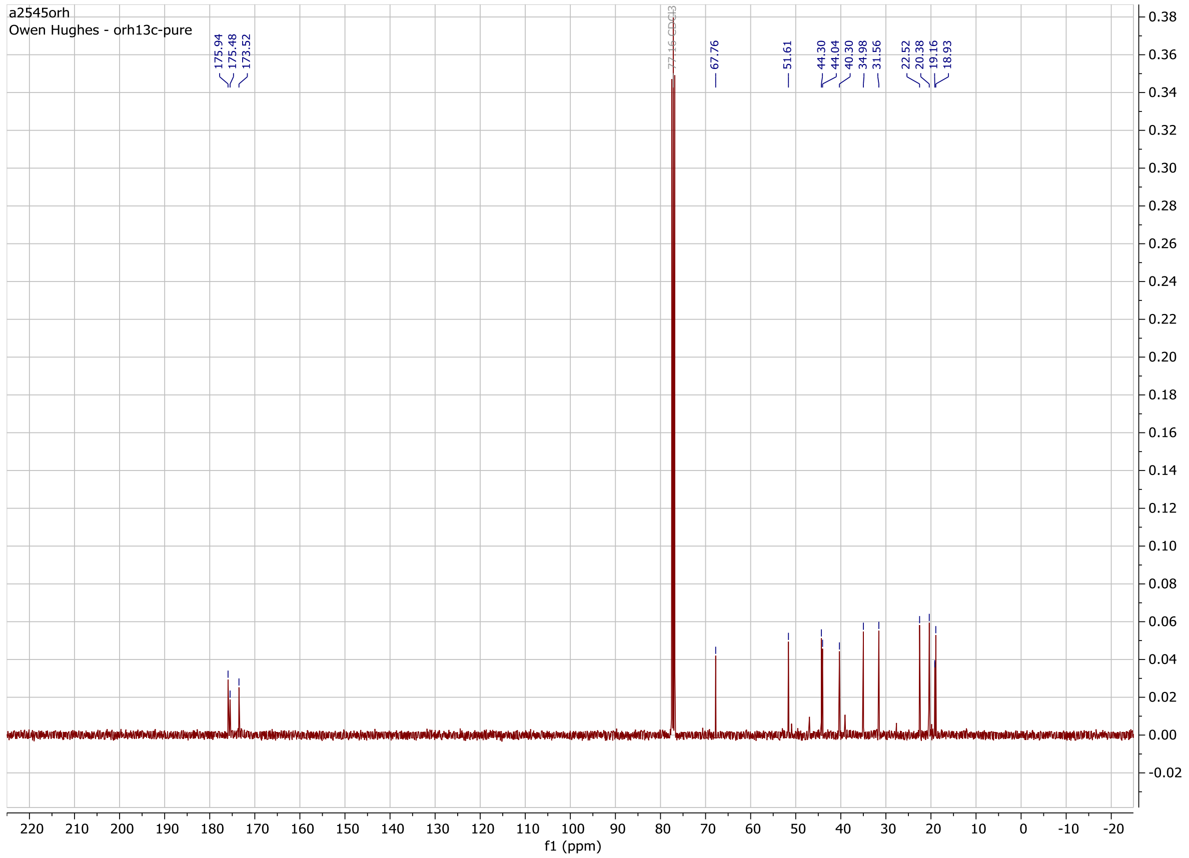

Ethyl 2-(4,10-dioxo-1,5-diazecan-1-yl)-3-methylpentanoate (7d)

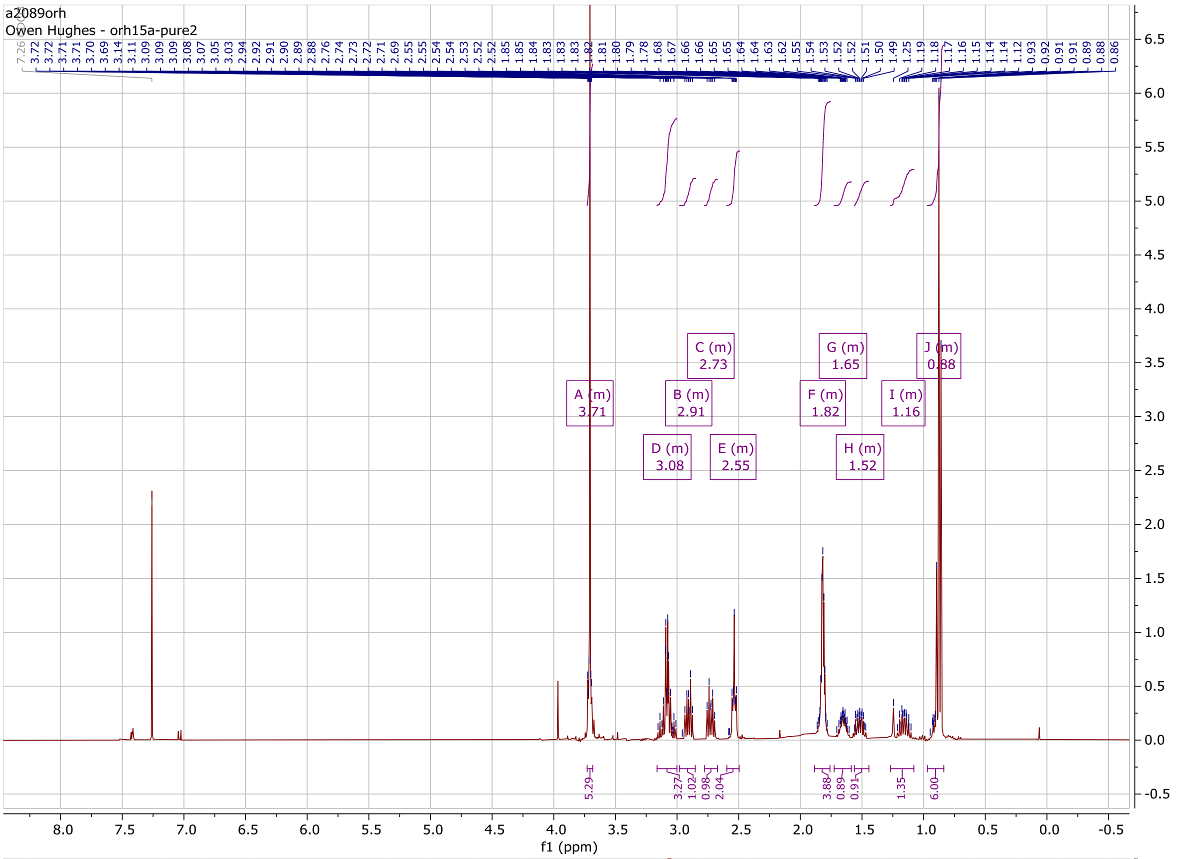

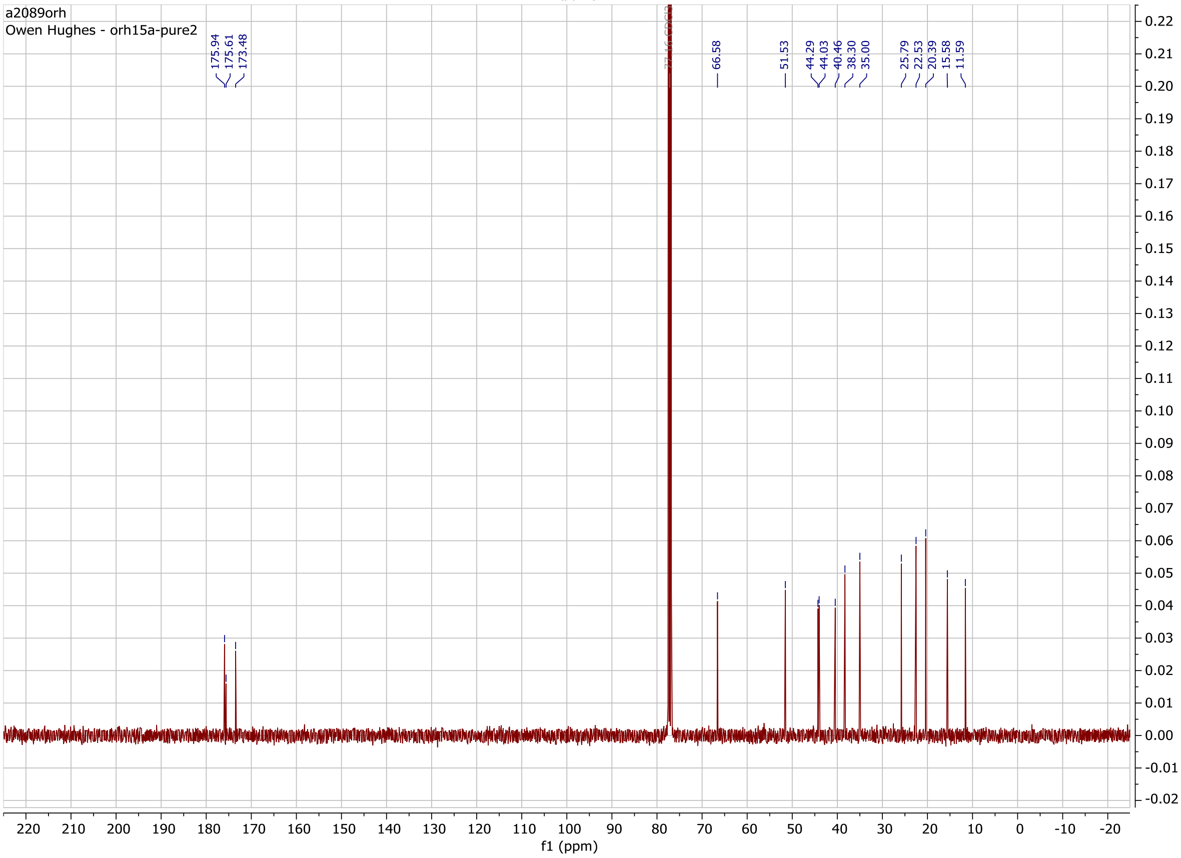

Methyl 2-(4,10-dioxo-1,5-diazecan-1-yl)-4-(methylthio)butanoate (7e) - In solution in $CDCl_3$, the compound exists as a mixture of two rotamers in an approximately 10:2:0.5 (A:B:C) ratio:

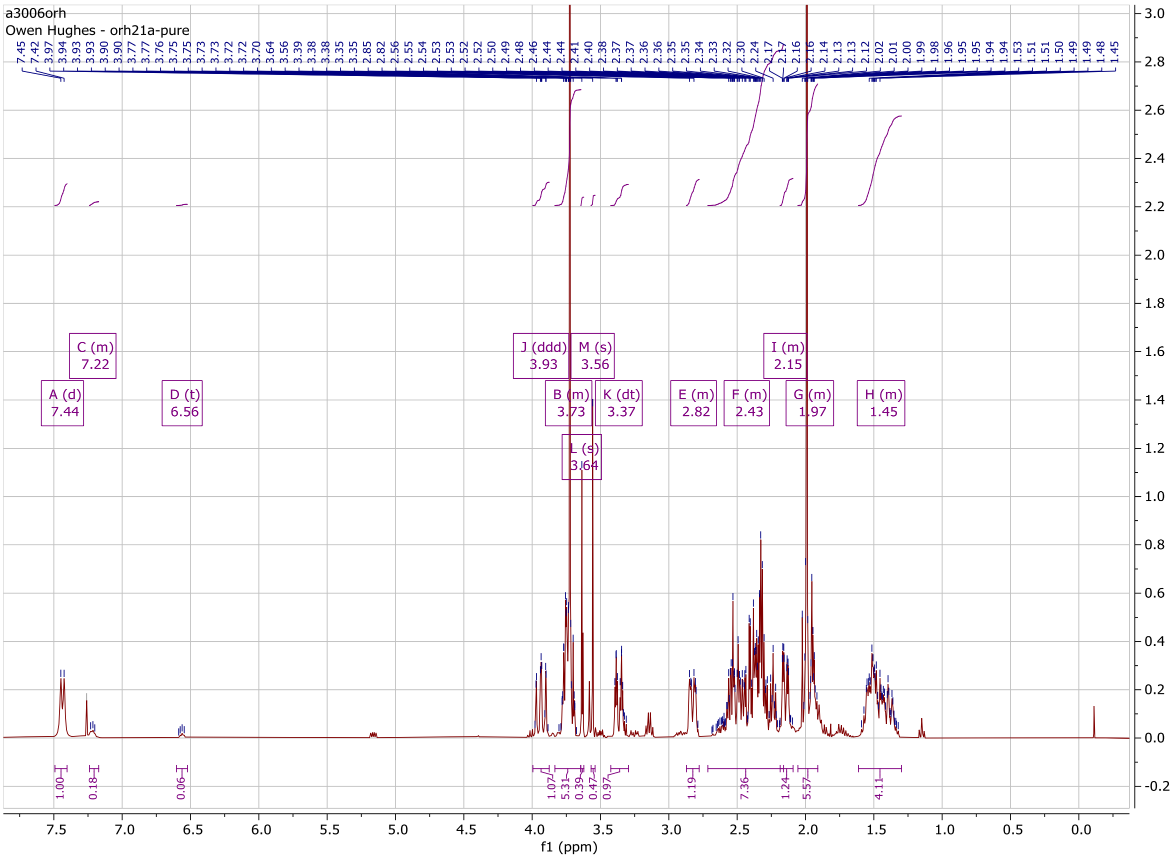

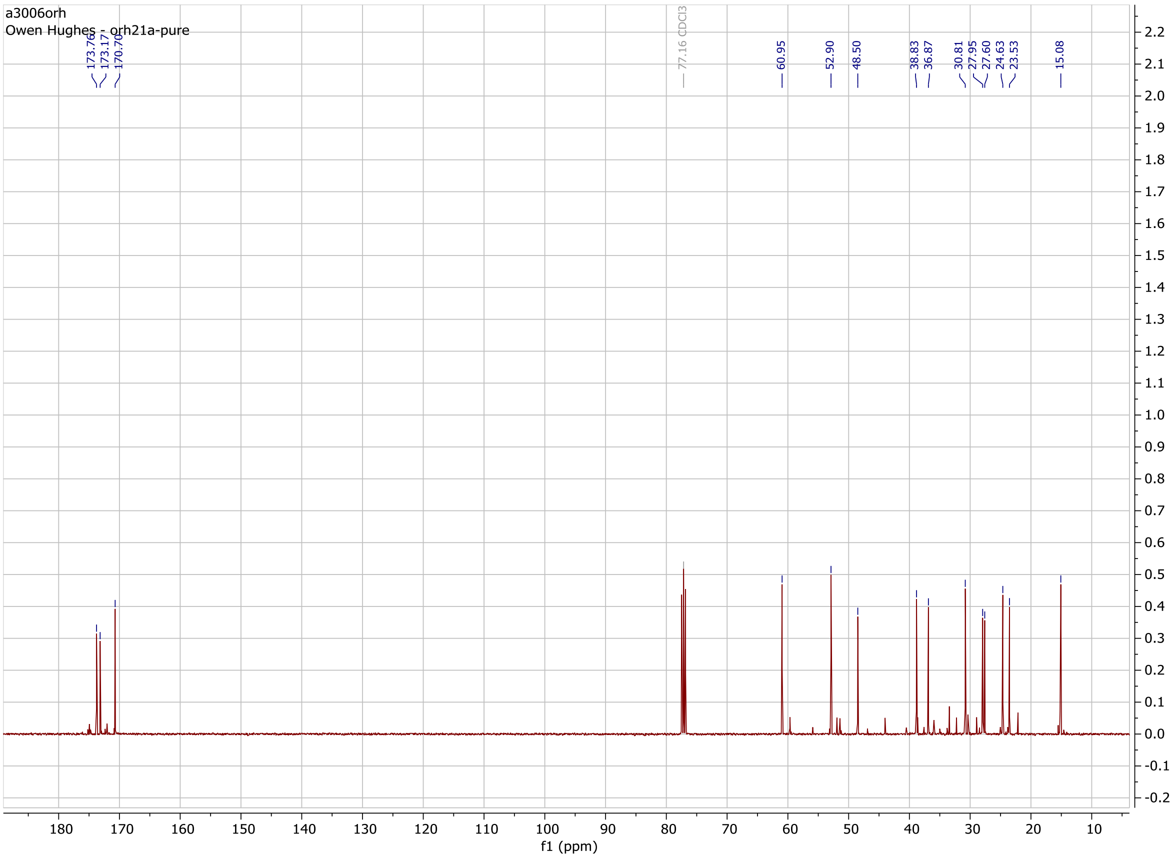

Ethyl 2-(4,10-dioxo-1,5-diazecan-1-yl)-3-(4-hydroxyphenyl)propanoate (7f)

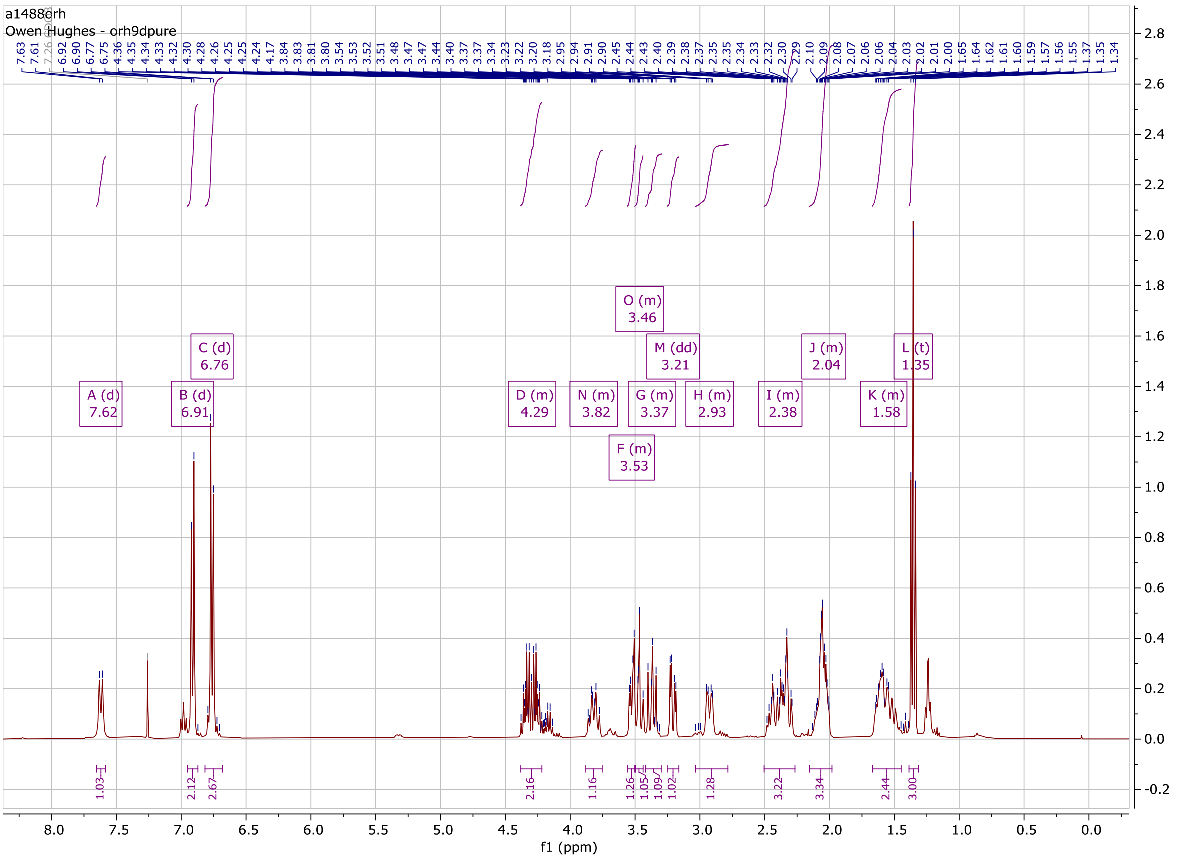

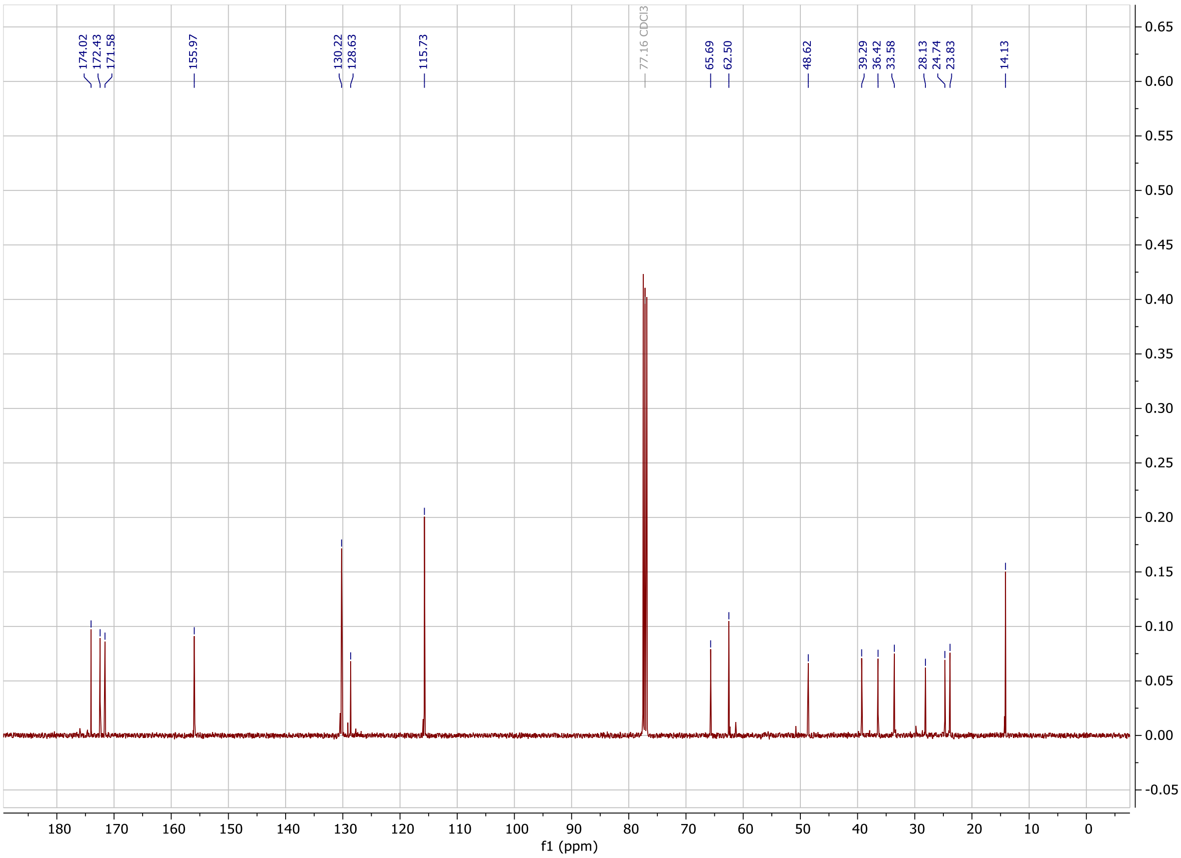

Methyl 2-(4,10-dioxo-1,5-diazecan-1-yl)-3-phenylpropanoate (7g) - In solution in $CDCl_3$, the compound exists as a mixture of two rotamers in an approximately 20:3 (A:B) ratio:

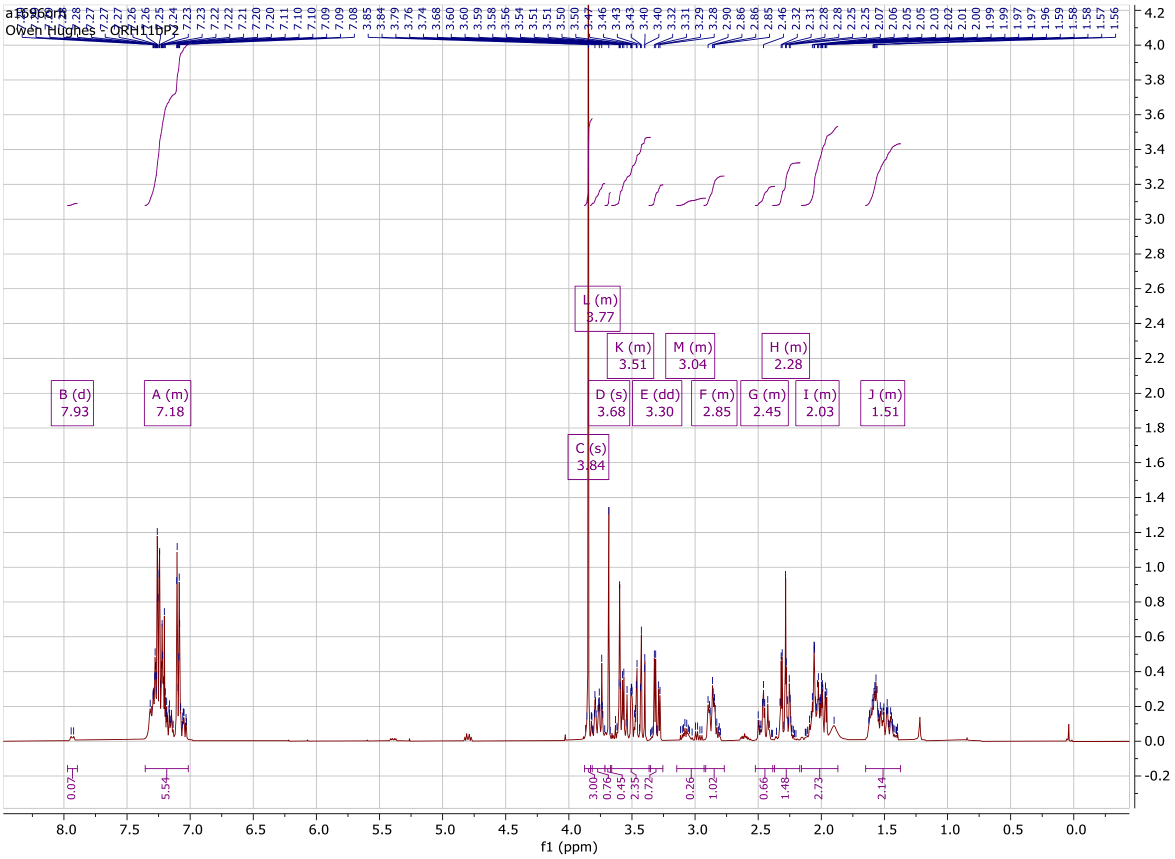

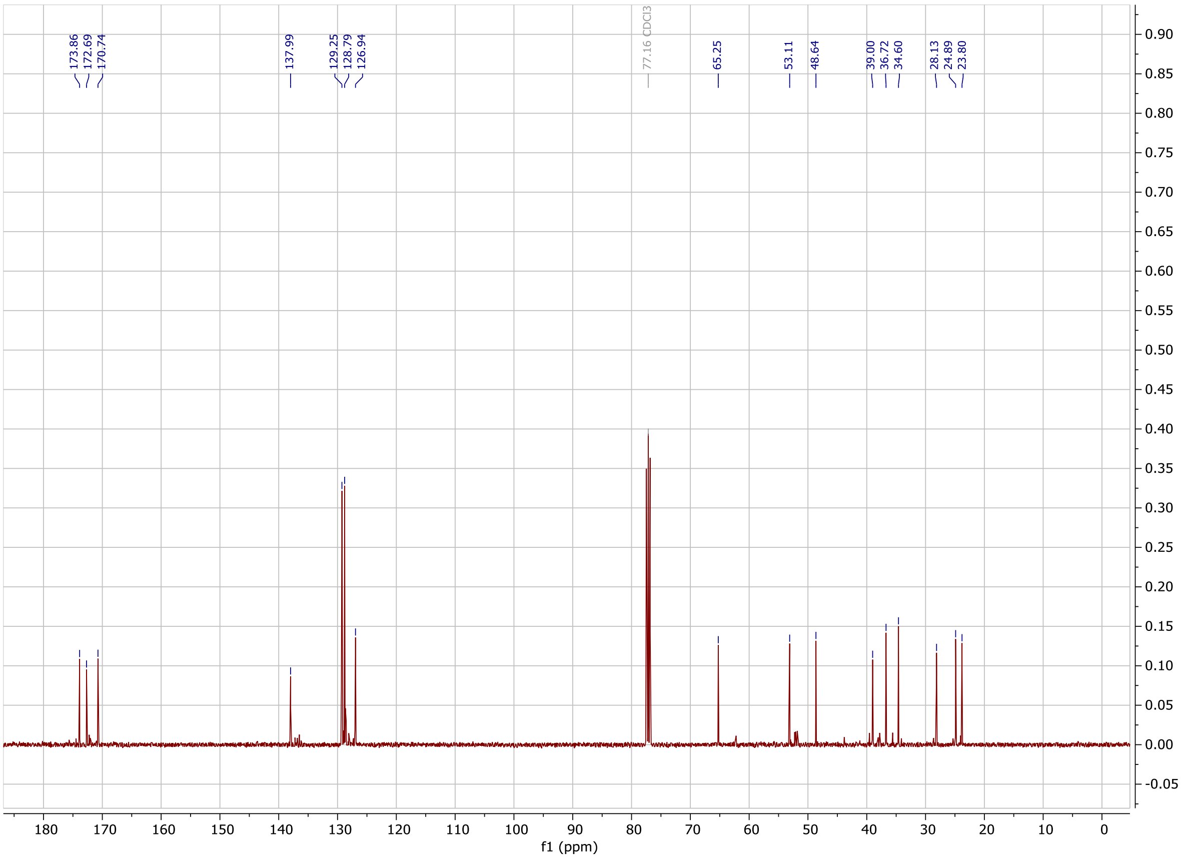

Methyl 2-(4,10-dioxo-1,5-diazecan-1-yl)-4-methylpentanoate (7h) - In solution in $CDCl_3$, the compound exists as a mixture of two rotamers in an approximately 20:7:4 (A:B:C) ratio:

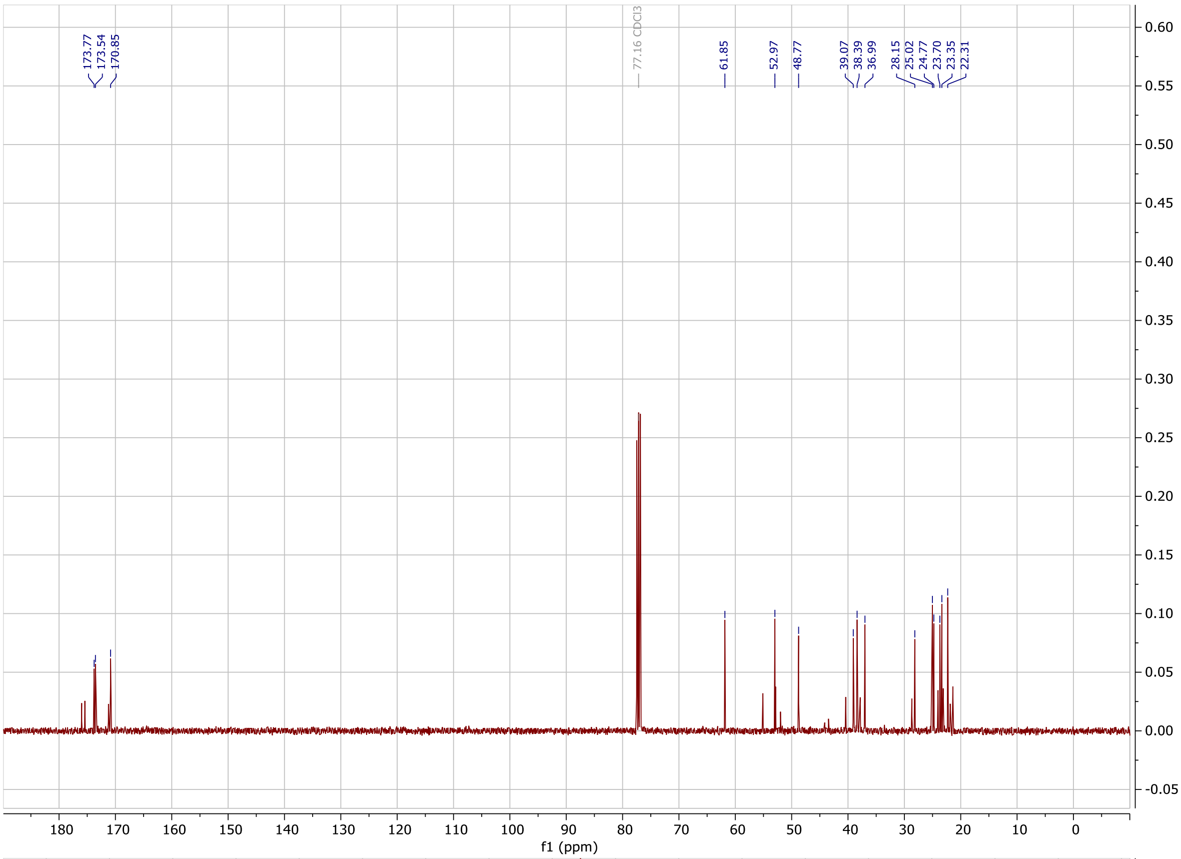

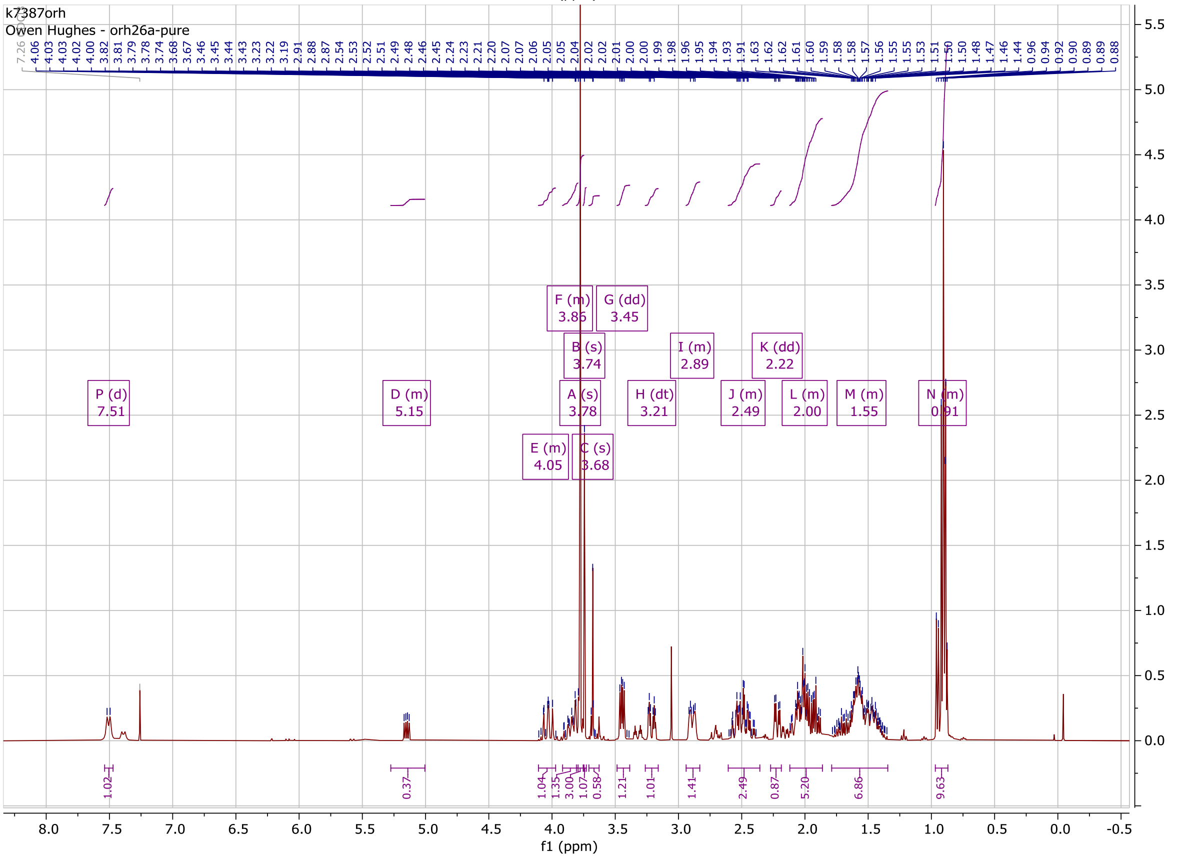

Methyl 2-(4,10-dioxo-1,5-diazecan-1-yl)-2-hydroxybutanoate (7i) - In solution in $CDCl_3$, the compound exists as a mixture of two rotamers in an approximately 9:1:1 (A:B:C) ratio:

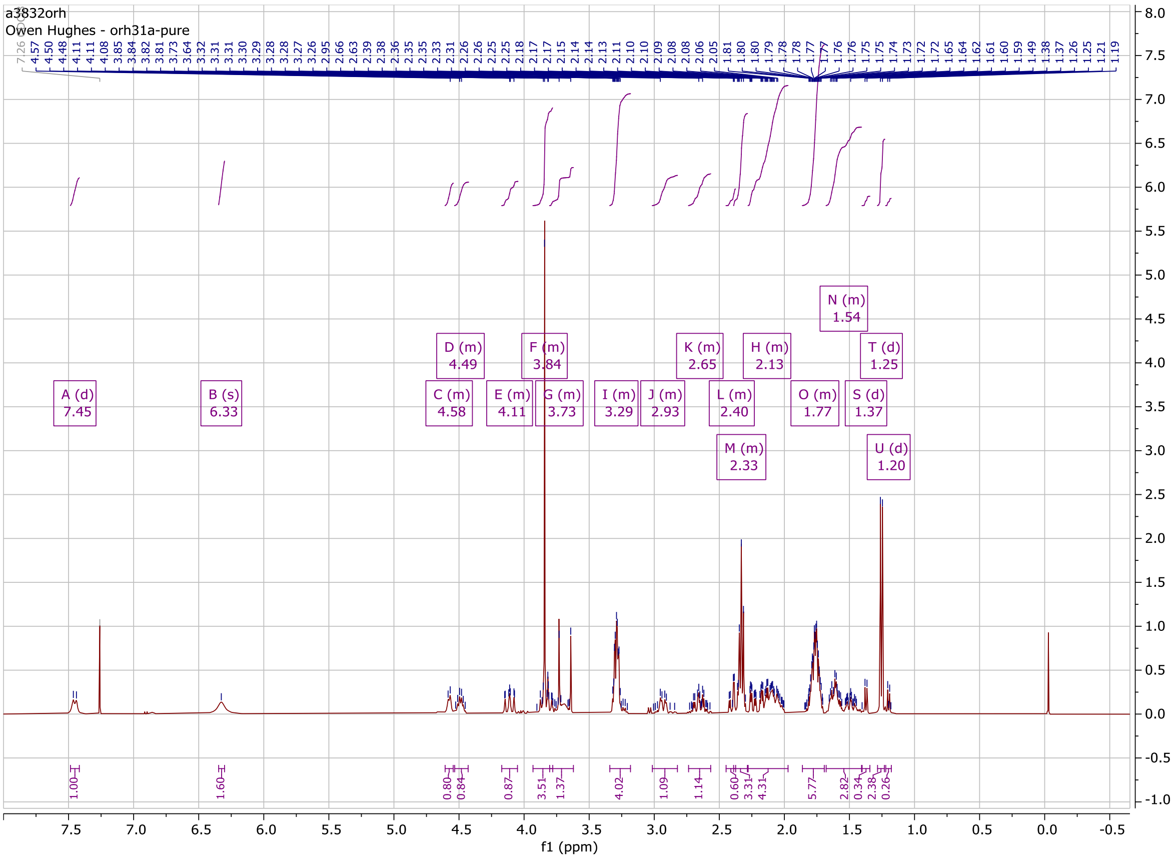

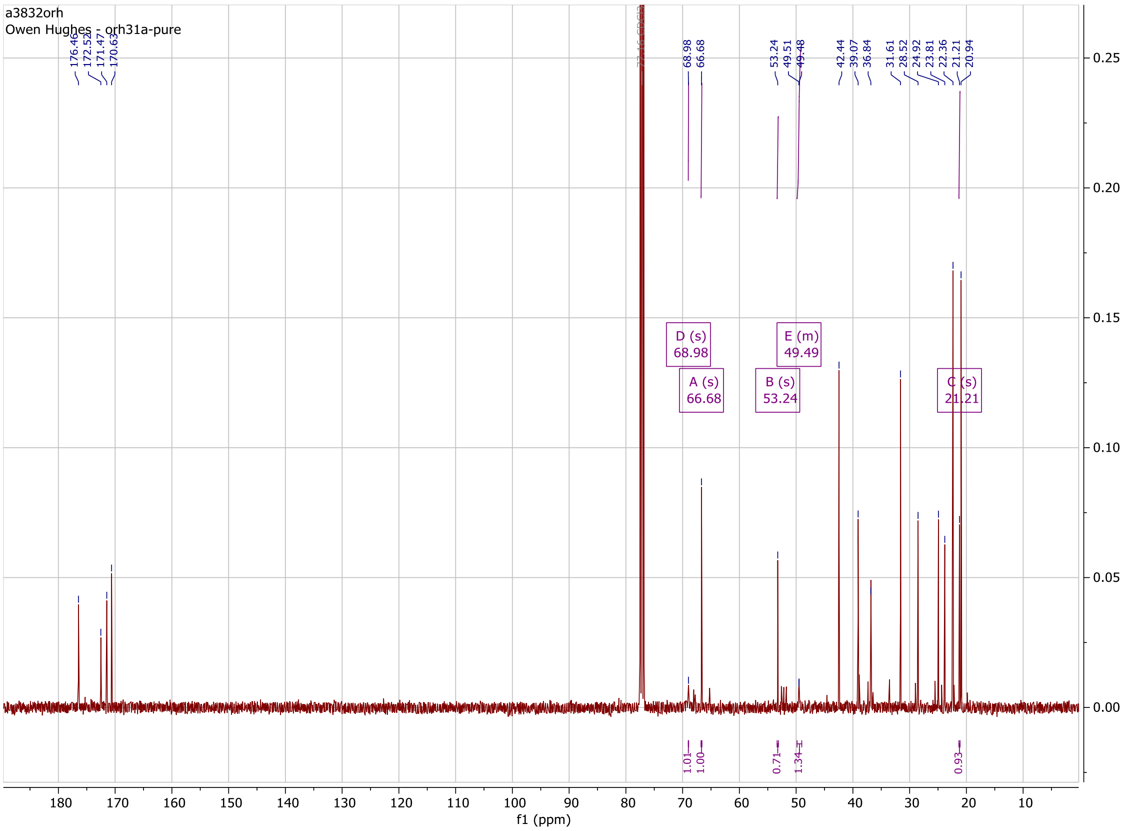

Methyl 2-(4,10-dioxo-1,5-diazecan-1-yl)-3-hydroxypropanoate (7j) - In solution in $CDCl_3$, the compound exists as a mixture of two rotamers in an approximately 2:1:0.15 (A:B:C) ratio:

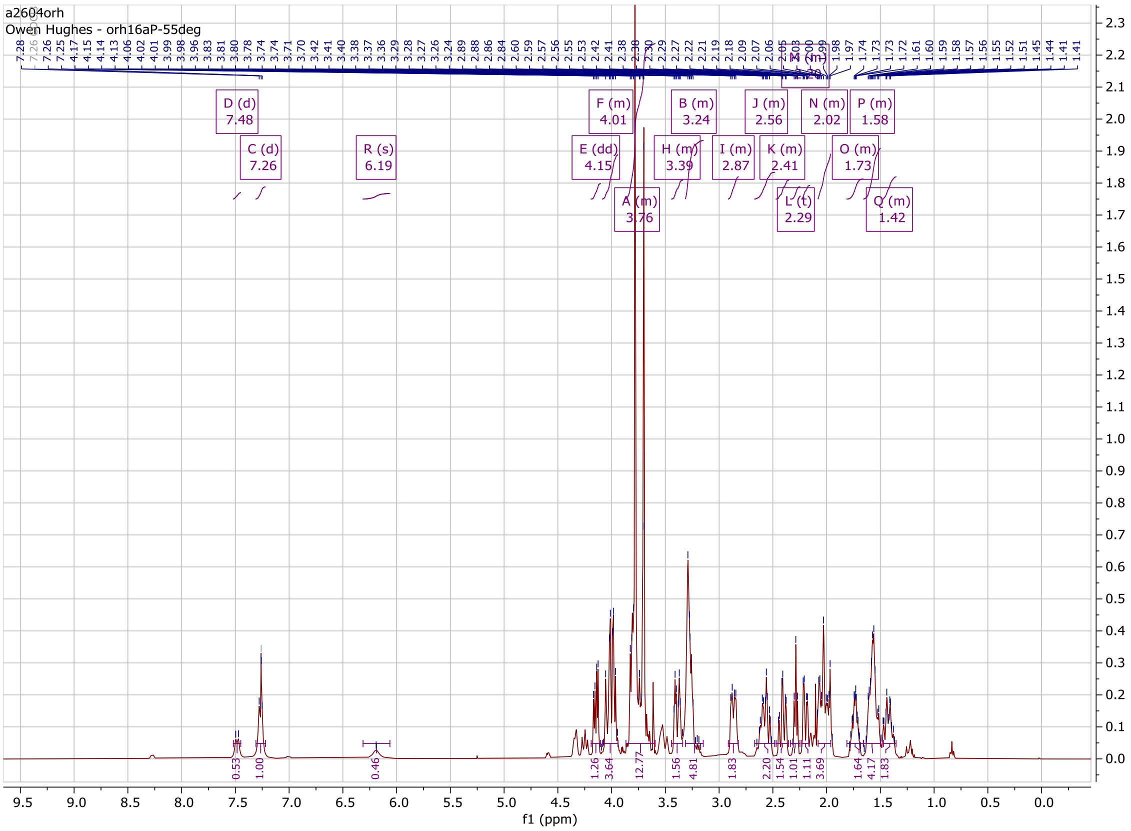

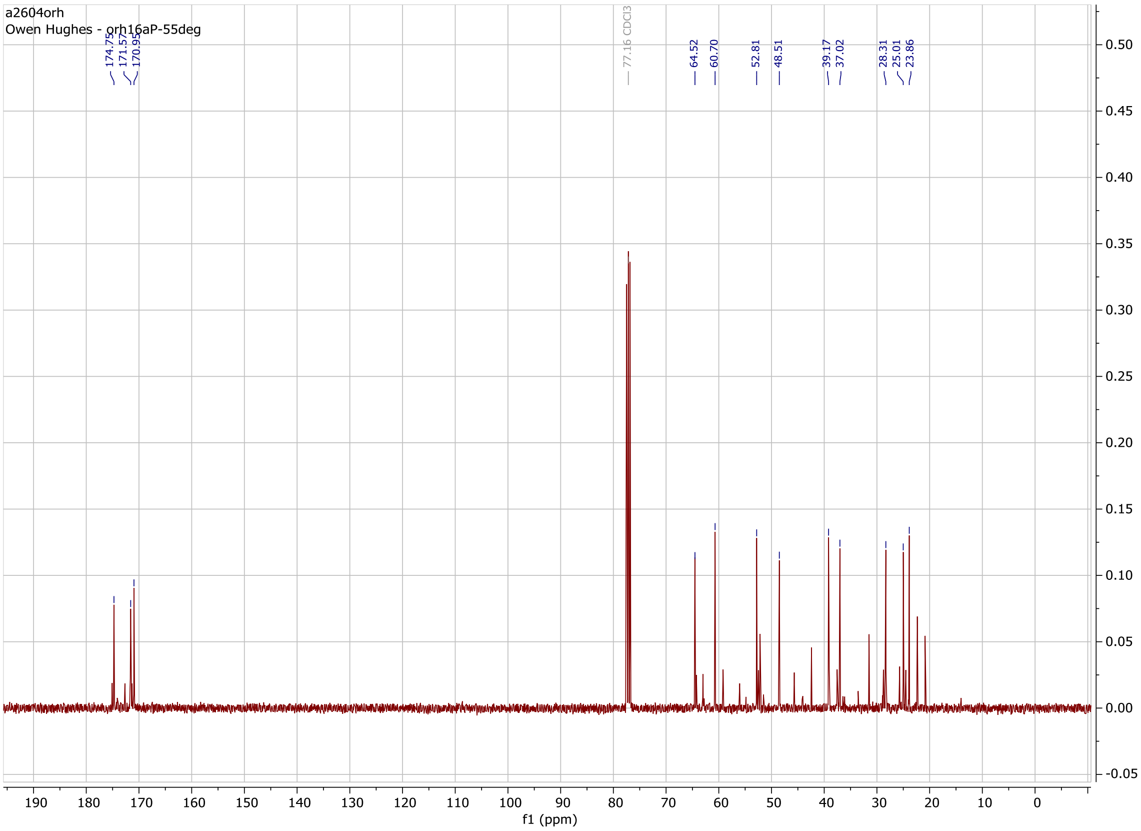

Methyl 2-(4,10-dioxo-1,5-diazecan-1-yl)-3-(1*H*-imidazol-4-yl) propanoate (7k)

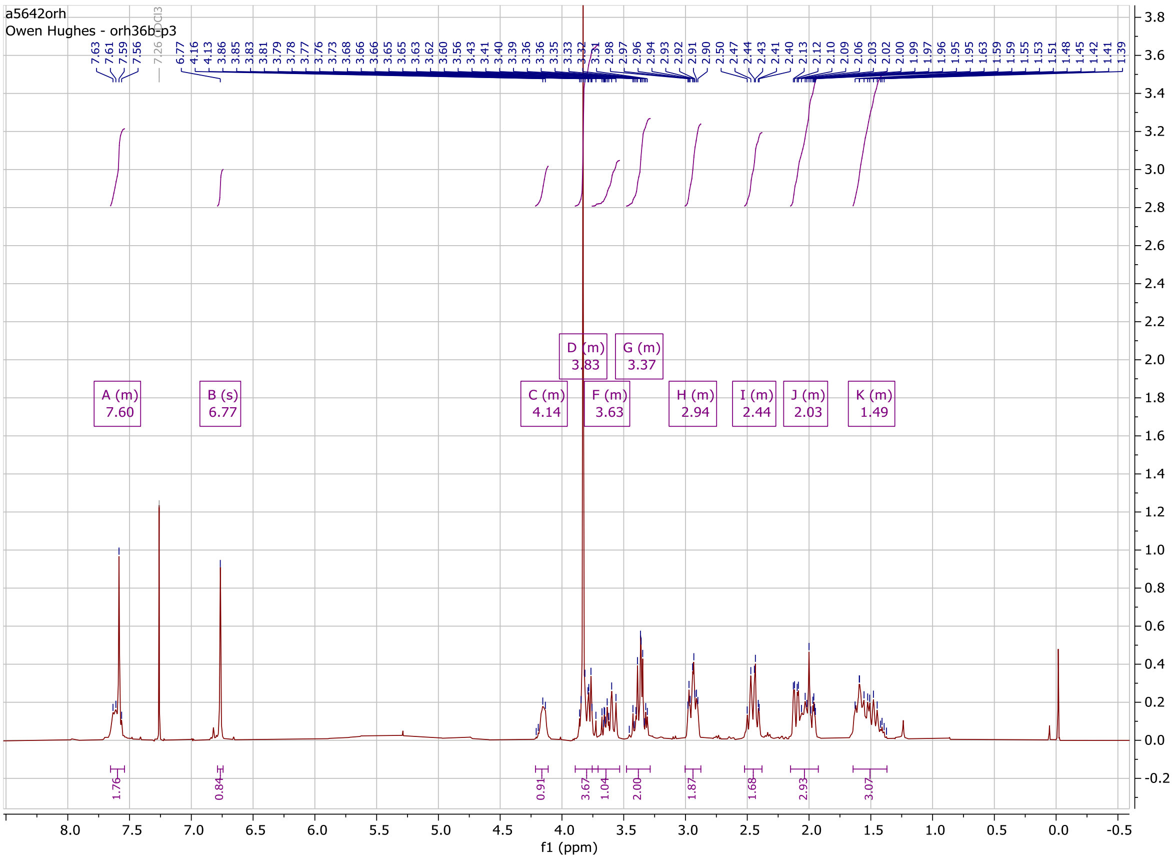

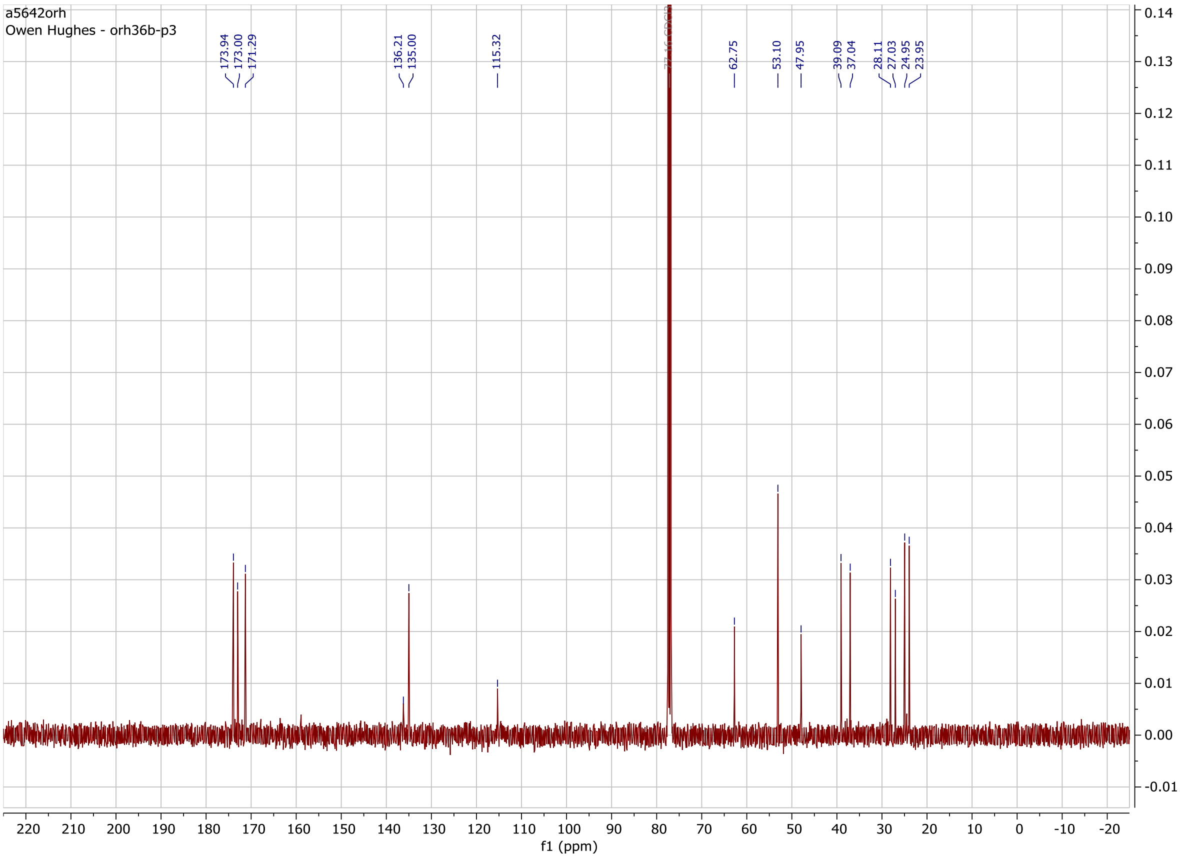

Methyl 5-amino-2-(4,10-dioxo-1,5-diazecan-1-yl)-5-oxopentanoate (7l) - In solution in $CDCl_3$, the compound exists as a mixture of two rotamers in an approximately 10:1 (A:B) ratio:

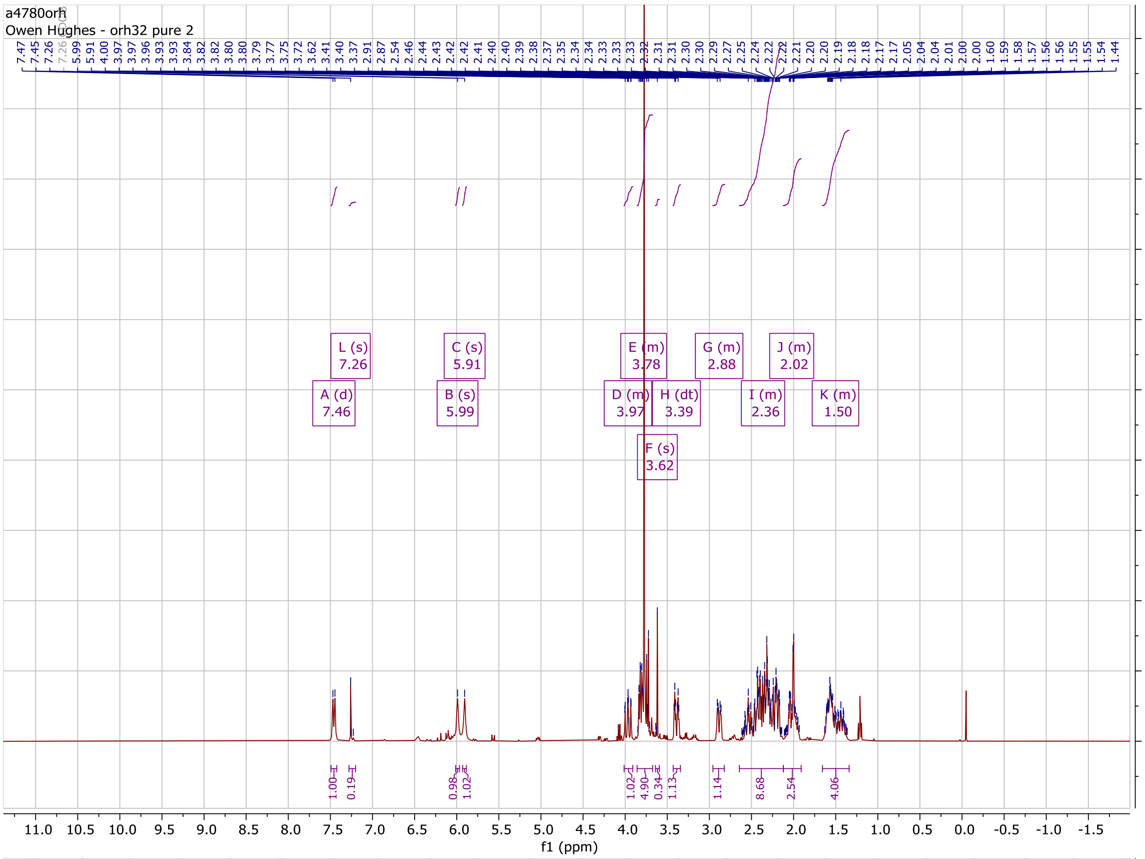

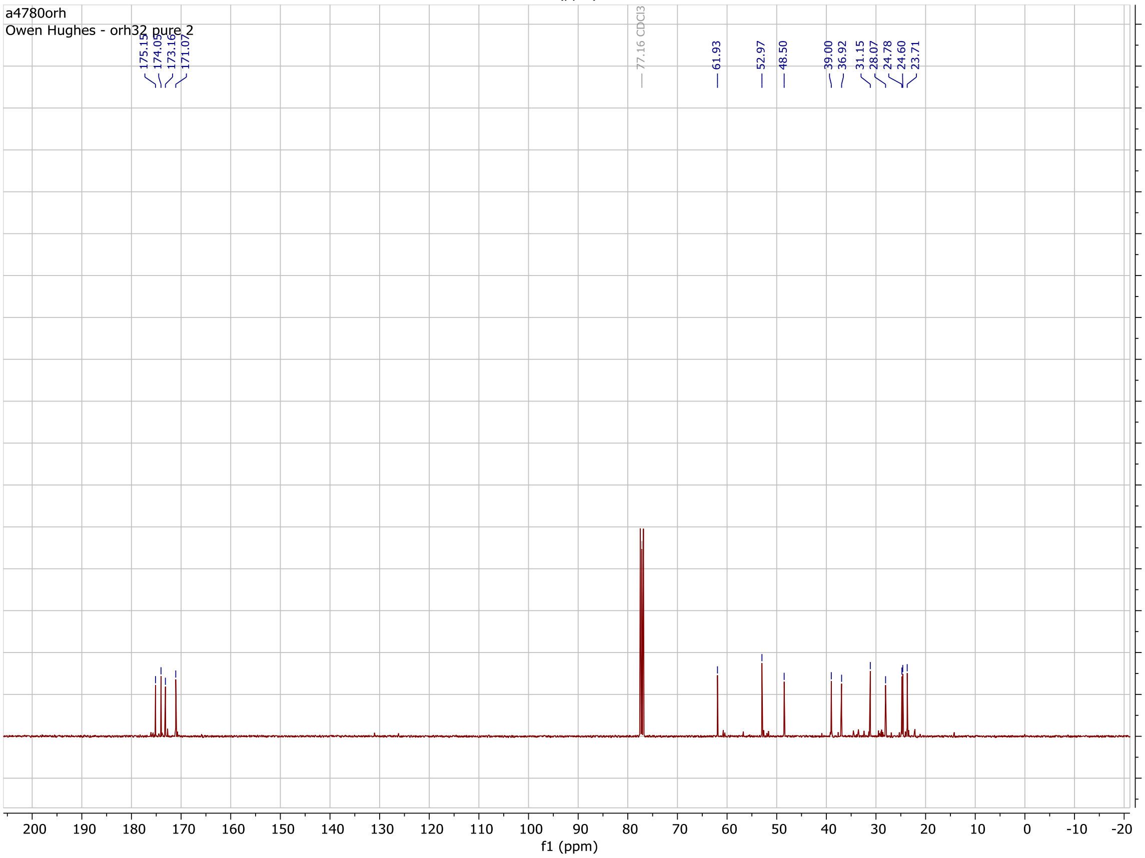

Diethyl 2-(4,10-dioxo-1,5-diazecan-1-yl)pentanedioate (7m) - In solution in $CDCl_3$, the compound exists as a complex mixture of rotamers, with the most distinguishable in an approximately 12:1 (A:B) ratio:

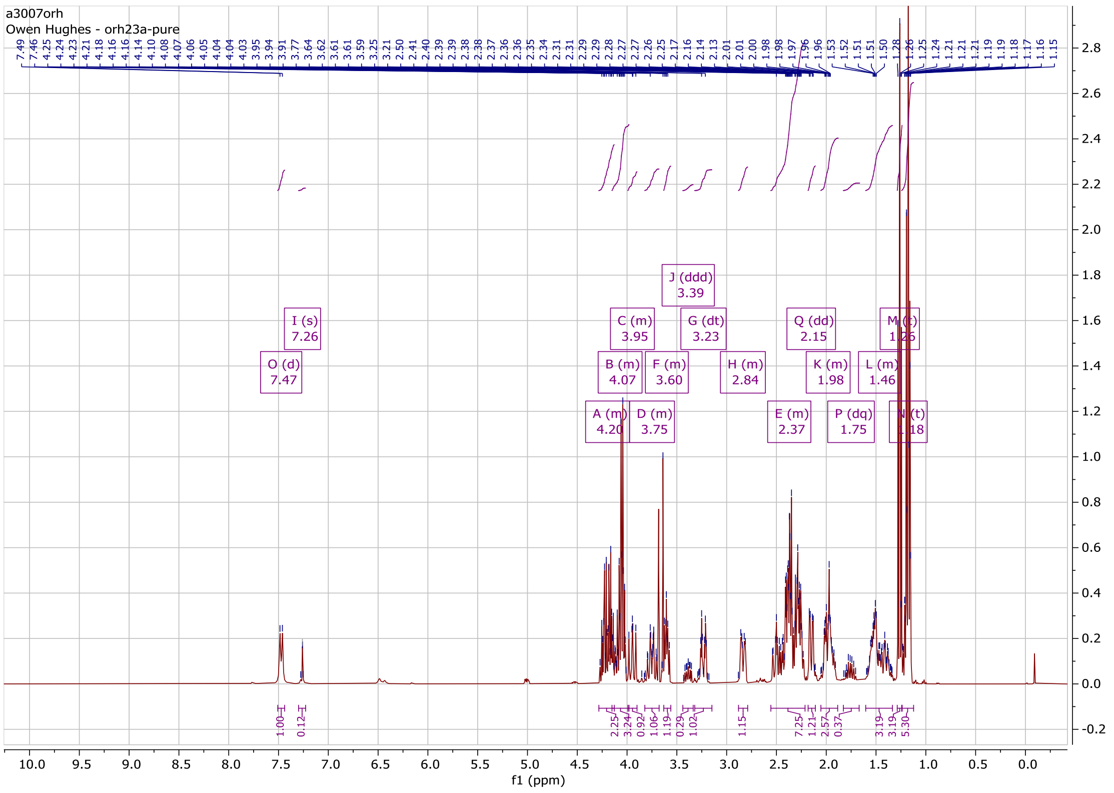

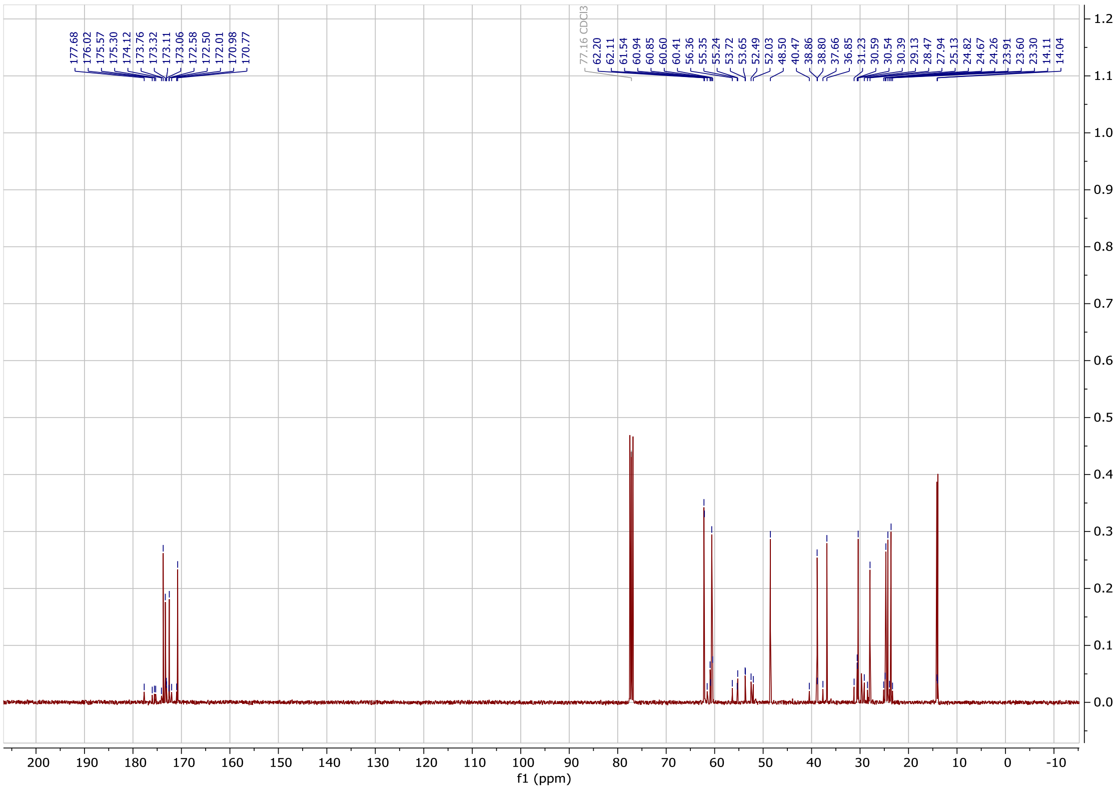

Methyl 2-(4,10-dioxo-1,5-diazecan-1-yl)-3-(1*H*-indol-3-yl)propanoate (7n)

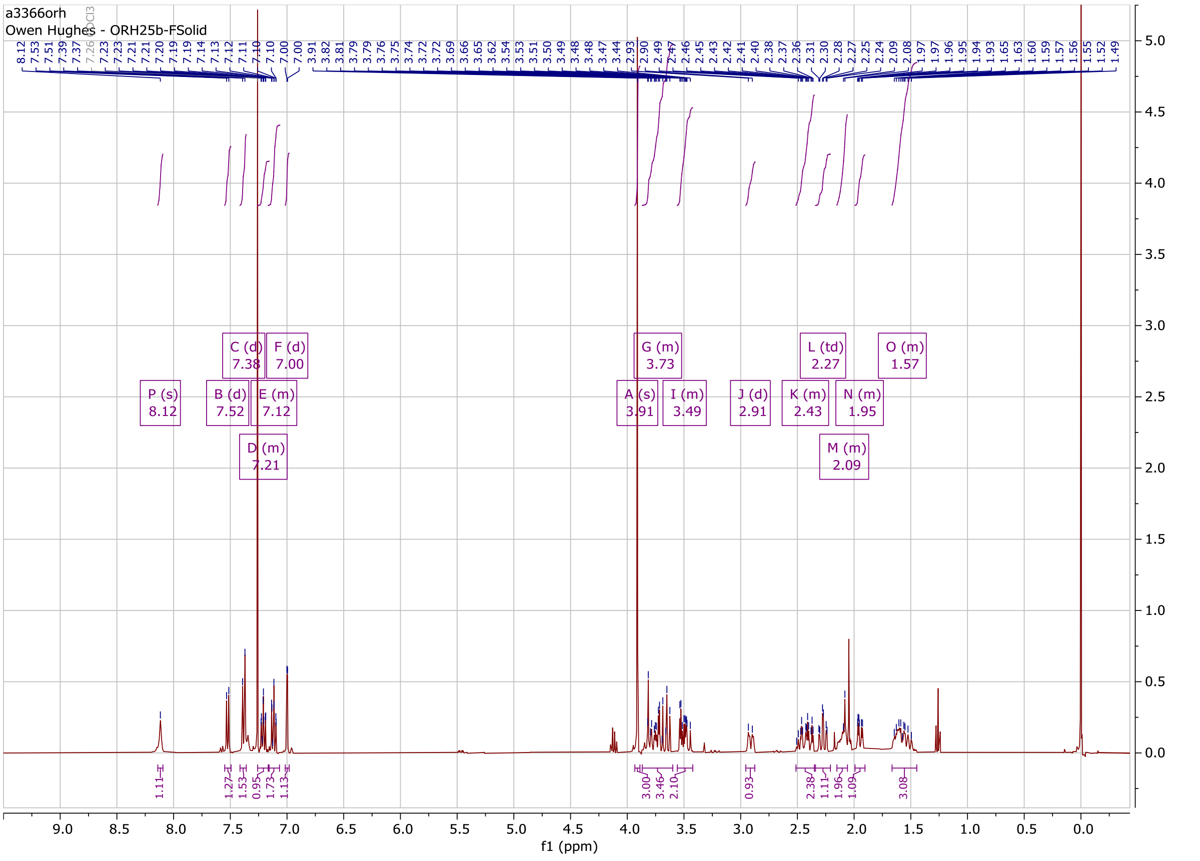

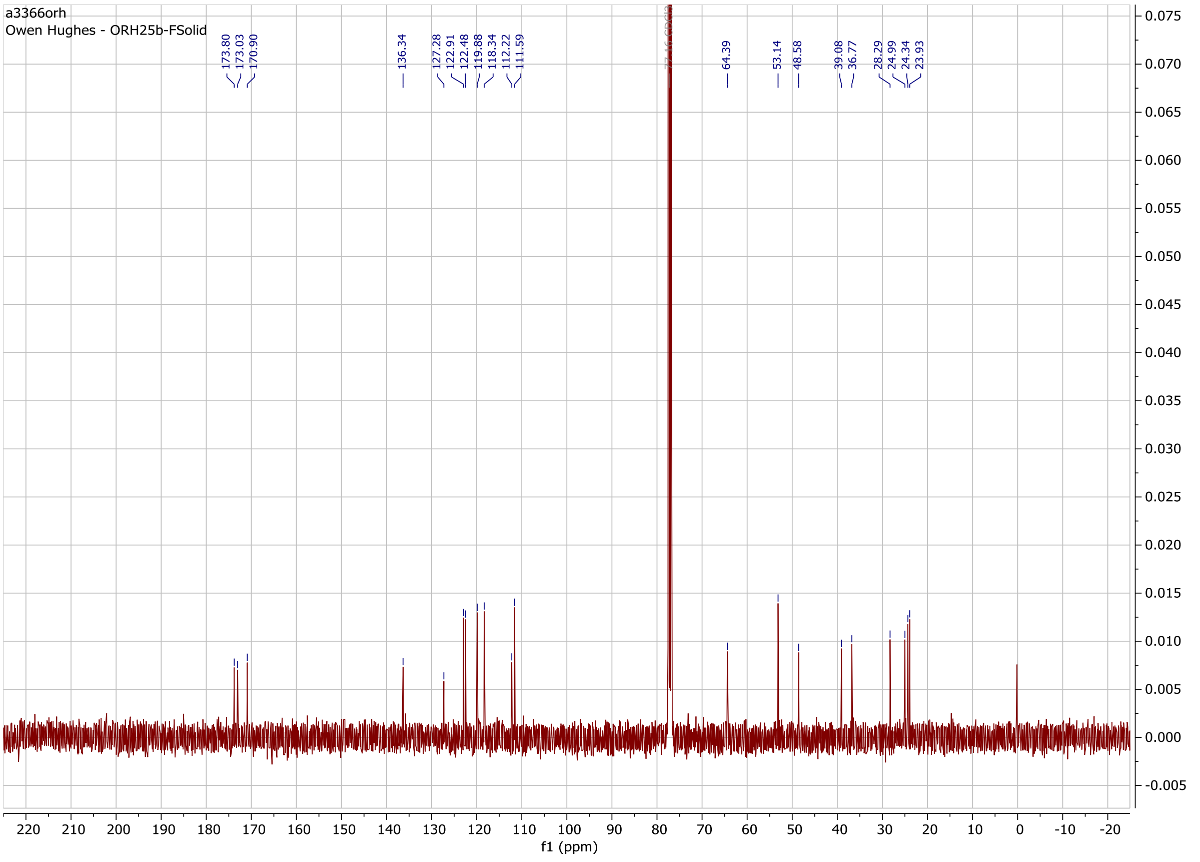

Methyl 5-(3-((2-amino-3-methoxy-3-oxopropyl)thio)propanamido)pentanoate (S4)

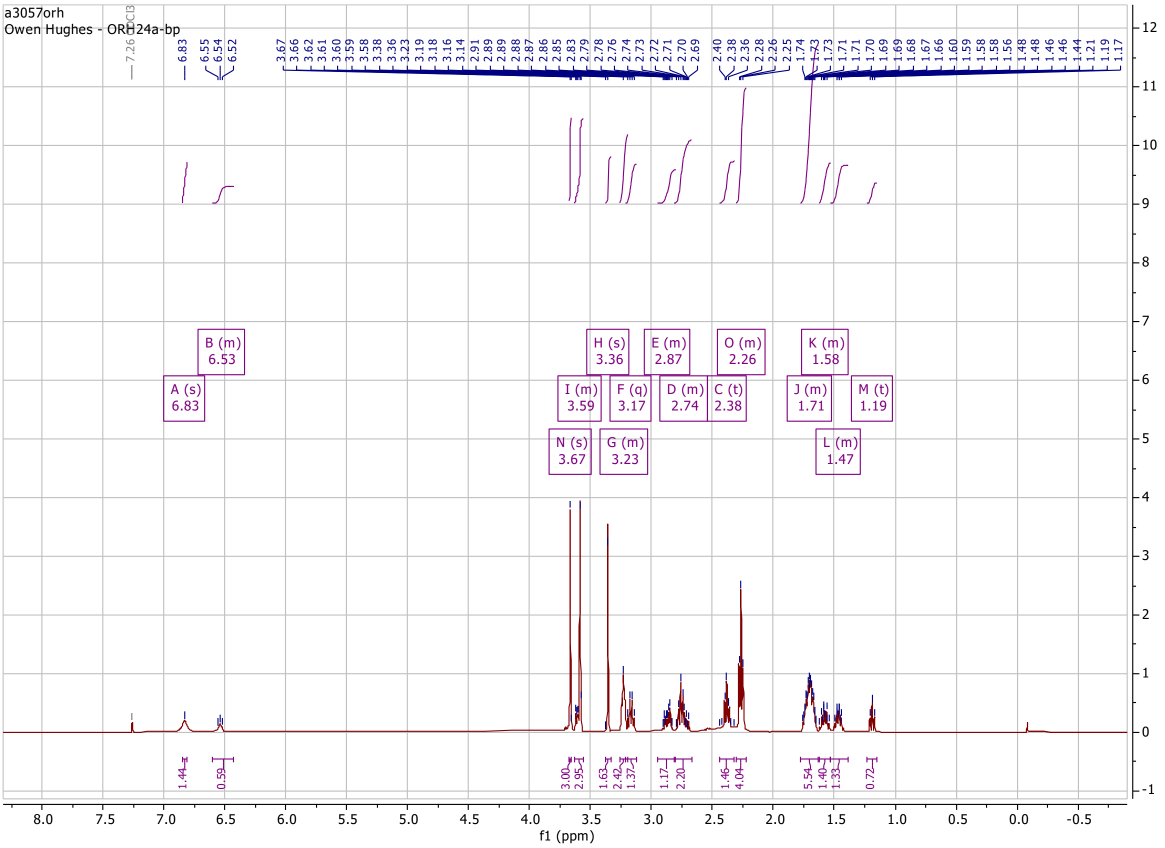

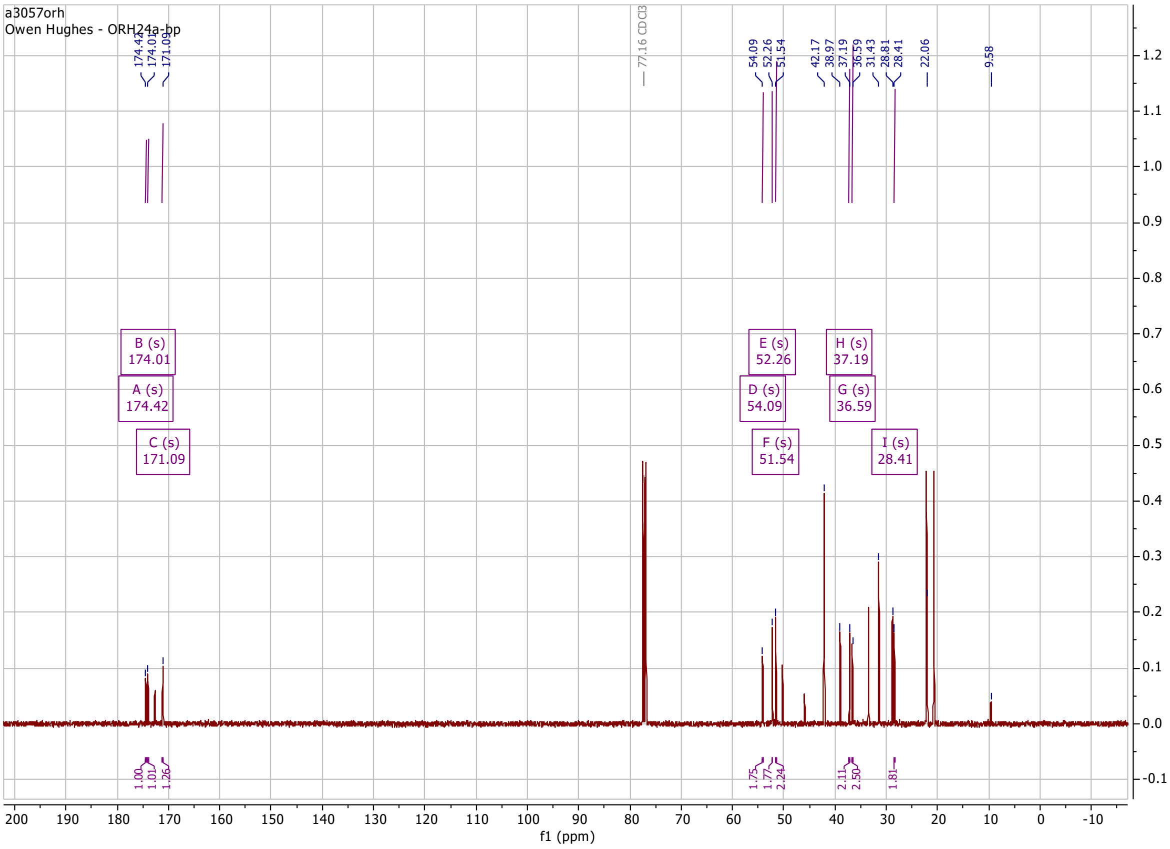

Methyl 5-acrylamidopentanoate (S6)

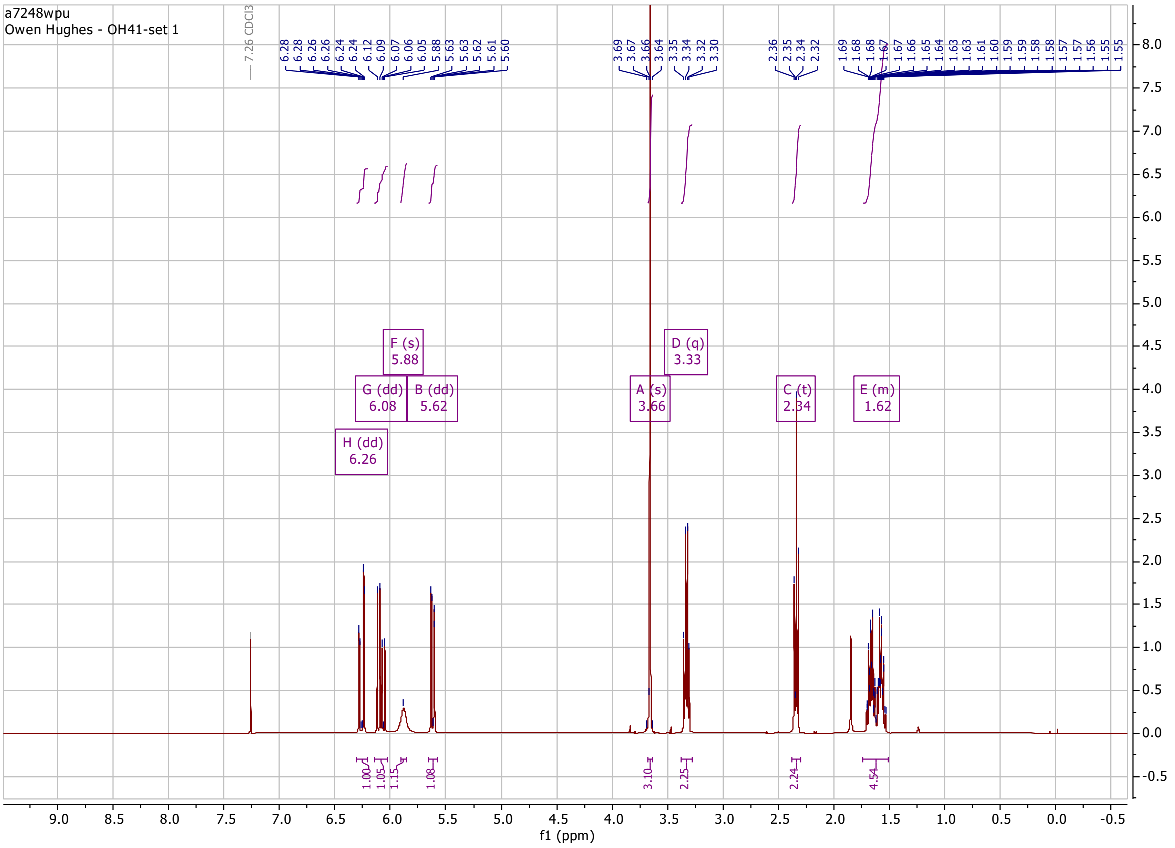

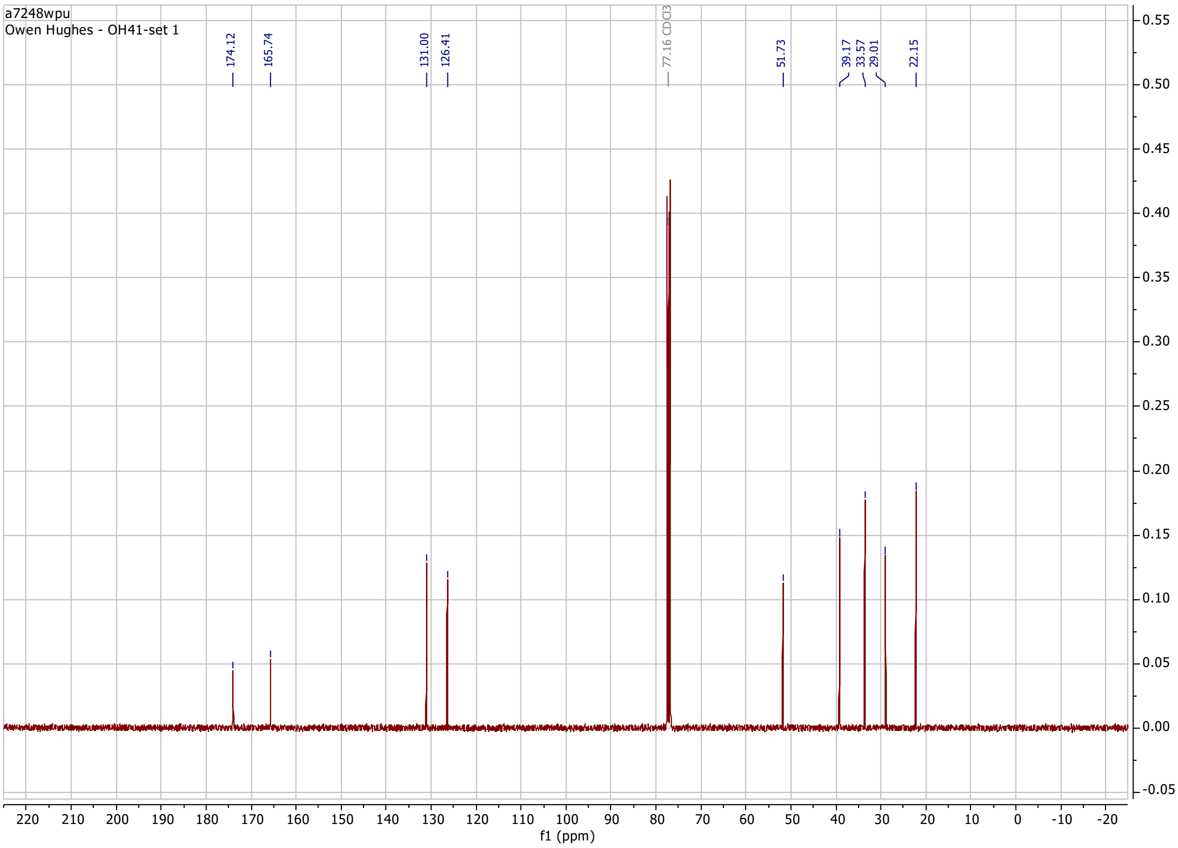

SI12. References

1. W. E. Orukotan, K. Y. Palate, B. Pogrányi, P. Bobinski, R. G. Epton, L. Duff, A. C. Whitwood, G. Grogan, J. M. Lynam, W. P. Unsworth, *Chem. Eur. J.*, 2023, **29**, e202303270.
2. K. Y. Palate, R. G. Epton, A. C. Whitwood, J. M. Lynam, W. P. Unsworth *Org. Biomol. Chem*., 2021, ***19***, 1404–1411.
3. H. Schägger, *Nat. Protoc.*, 2006, **1**, 16-22.
4. L. J. Barber, N. D. J. Yates, M. A. Fascione, A. Parkin, G. R. Hemsworth, P. G. Genever, C. D. Spicer, *RSC Chem. Biol.*, 2023, **4**, 56-64.
5. N. D. Yates, S. Akkad, A. R. Noble, T. Keenan, N. E. Hatton, N. Signoret, M. A. Fascione, *Green Chem.*., 2022, **24**, 8046-8053.
6. A. Tufail, S. Akkad, A. R. Noble, M. A. Fascione, N. Signoret, *Sci. Rep.,* 2024, **14**, 24188.
7. N. Signoret, T. Christoph, M. Oppermann, M. Marsh. *Traffic* 2004, **5**, 529-543.
8. K. Y. Palate, Z. Yang, A. C. Whitwood and W. P. Unsworth *RSC Chem. Biol*. 2022, **3**, 334
